# Supplementary material for: Decarboxylative thiolation of redox-active esters to free thiols and further diversification
Source: Nat Commun. 2020 Oct 21;11:5340. doi: 10.1038/s41467-020-19195-w (PMC7578659; doi:10.1038/s41467-020-19195-w)
Supplement: Supplementary file 1 — Supplementary Information [file 41467_2020_19195_MOESM1_ESM.pdf]

Supplementary Information

Decarboxylative thiolation of redox-active esters to free thiols and further diversification

Cao *et al.*

Supplementary Methods

General information

Commercially available reagents and solvents were used without further purification. Dry solvents were used for all photoreactions. All photocatalysts are commercial available. ^{1}H NMR spectra were recorded on a Bruker AVANCE III 400 MHz and are internally referenced to TMS (δ 0.00 ppm) when using $CDCl_3$ as solvent or residual protic $(CD_3)_2SO$ (δ 2.5 ppm) when using $(CD_3)_2SO$ as solvent. Data for ^{1}H NMR are reported as follows: chemical shift (δ ppm), multiplicity (s = singlet, d = doublet, t = triplet, q = quartet, m = multiplet, dd = doublet of doublets, dt = doublet of triplets, dq = doublet of quartets, br = broad), coupling constant (Hz), and integration. ^{13}C NMR spectra were recorded on a Bruker AVANCE III 400 MHz (100 MHz) and data are reported in terms of chemical shift relative to $CDCl_3$ (77.00 ppm). The proton-decoupled ^{19}F NMR spectra were recorded on a Bruker AVANCE III 400 MHz (376 MHz). High resolution mass spectra (HRMS) were recorded on a Thermo Fisher Scientific Exactive Plus by Electrospray Ionisation (ESI) or Atmospheric Pressure Photo-Ionization (APCI) or Electron Ionization (EI). GC-MS measurements were performed on a SHIMADZU GCMS-QP2010 SE. GC measurements were performed and investigated via integration of the signal obtained by using anisole as an internal standard. Flash column chromatography was carried out using Hai Lang silica gel (200-300 mesh). All reactions were followed by thin-layer chromatography (TLC) when practical, using JIAPENG ZF-7 fluorescent treated silica which was visualized under UV light (254 or 365 nm), by staining with an aqueous solution of $KMnO_4$ followed by heating.

The reactions were conducted in photo-reactors, which comprise a fan for cooling (approximately room temperature) and six 1W blue LED beads for each place. The average power output of the photo-reactor was recorded at 30 mW/cm^2. The emission spectra of the blue LEDs were recorded on an Ocean Optics HR4000CG-UVNIR spectrometer. The spectra was normalised to 1.0 at the maximum (450 nm).

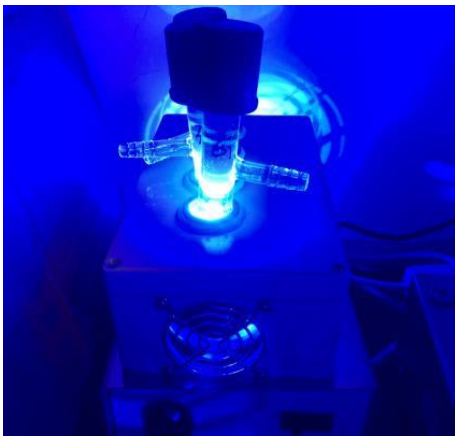

Supplementary Figure 1. Photo-reactor and reaction setup. With blue LEDs.

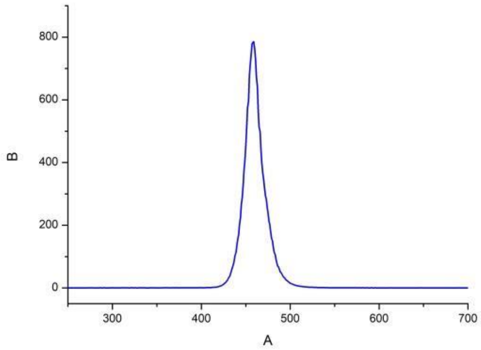

Supplementary Figure 2. The light emission spectrum of photo-reactor. Blue LEDs.

General procedure for synthesis of NHPI redox-active esters

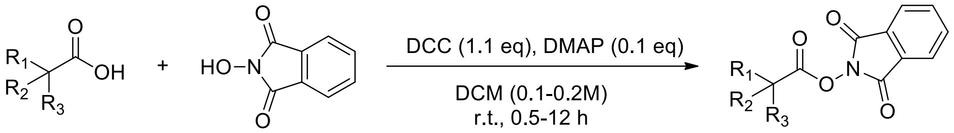

NHPI esters were prepared according to the previously reported procedure.[1-7] To an oven-dried round-bottom flask with a magnetic stir bar was added (8.00 mmol, 1.0 equiv.), *N*-hydroxyphthalimide (1.43 g, 8.80 mmol, 1.1 equiv.), Dicyclohexylcarbodiimide (DCC) (1.98 g, 9.60 mmol, 1.2 equiv.) and 4-dimethylaminopyridine (DMAP) (0.98 g, 0.80 mmol, 0.1 equiv.). Dry dichloromethane (40 mL) was added and the mixture was allowed to stir until the acid was consumed (determined by TLC) at rt. Typical reaction times were between 0.5 h and 12 h. The white precipitate was filtered off and the solution was concentrated under reduced pressure and purified by flash column chromatography to give the desired NHPI redox-active ester.

Synthesis of Thioamides

Most of thiourea and thioamides are commercially available and purchased from Energy, Adamas, TCI, Aldrich, and used as accepted without further purification. Other thioamide (**2f-h**) were prepared according to the reported procedures.[8-11] The detailed procedure for the synthesis of thioamide **2f** was described as bellow:

4-Methoxybenzothioamide (2f): [8]

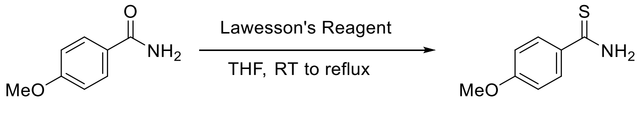

Commercially available 4-methoxybenzamide (1.81 g, 12.0 mmol) in THF (80 mL) was added Lawesson's reagent (2.43 g, 6.0 mmol). The solution was stirred overnight at rt and then heated to reflux for 60 h. Upon completion, solvent was evaporated using rotary evaporator and the crude residue was purified by flash column chromatography on silica gel (Petroleum ether / EtOAc = 3:1) to afford thioamide **2f** (1.60 g, 80%) as a pale yellow solid. The product can be recrystallized from EtOAc for further purification.

^{1}H NMR (400 MHz, $(CD_3)_2SO$): δ (ppm) 9.64 (br, 1H, N-H^a), 9.32 (br, 1H, N-H^b), 7.96 (d, *J* = 7.3 Hz, 2H, Ar-H), 6.95 (d, *J* = 7.4 Hz, 2H, Ar-H), 3.80 (s, 3H, OCH_3).

^{13}C NMR (100 MHz, $(CD_3)_2SO$): δ (ppm) 199.10, 162.38, 131.84, 129.94, 113.52, 55.92.

General procedure for the decarboxylative Thiolation

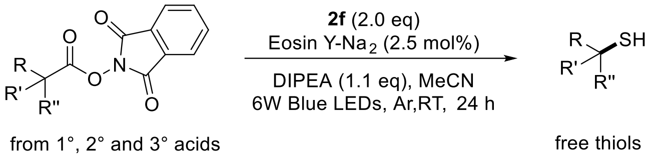

To an oven-dried 10-ml Schlenk tube equipped with a magnetic stir bar and a Teflon coated septum screwcap was added the NHPI redox-active ester (0.2 mmol, 1.0 equiv.), 4-methoxythiobenzamide (**2f**, 0.4 mmol, 2.0 equiv.), eosin Y-Na_2 (2.5 mol%). The tube was evacuated and back-filled with argon for three cycles. The DIPEA (0.22 mmol, 1.1 equiv.) and dry CH_3CN (2.0 ml) was added via a gastight syringe under argon atmosphere. Make sure the screwcap was closed, the solvent was frozen by liquid nitrogen. Then the screwcap was opened and the tube was evacuated for about 3 mins. The screwcap was closed and let the solvent melts in a tepid water bath. Repeat above Freeze-Pump-Thaw procedures for 3-5 times until you no longer see the evolution of gas as the solution thaws. The tube was filled with argon and sealed, irradiated with 6W blue LED reactor and stirred at ambient temperature for 24 hours.

General Procedure for the Synthesis of Disulfides

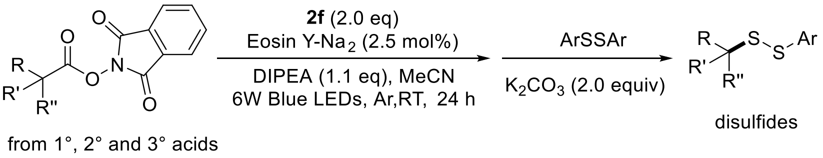

To an oven-dried 10-ml Schlenk tube equipped with a magnetic stir bar and a Teflon coated septum screwcap was added the NHPI redox-active ester (0.2 mmol, 1.0 equiv.), 4-methoxythiobenzamide (**2f**, 0.4 mmol, 2.0 equiv.), eosin Y-Na_2 (2.5 mol%). The tube was evacuated and back-filled with argon for three cycles. The DIPEA (0.22 mmol, 1.1 equiv.) and dry CH_3CN (2.0 ml) was added via a gastight syringe under argon atmosphere. Make sure the

screwcap was closed, the solvent was frozen by liquid nitrogen. Then the screwcap was opened and the tube was evacuated for about 3 mins. The screwcap was closed and let the solvent melts in a tepid water bath. Repeat above Freeze-Pump-Thaw procedures for 3-5 times until you no longer see the evolution of gas as the solution thaws. The tube was filled with argon and sealed, irradiated with 6W blue LED reactor and stirred at ambient temperature for 24 hours. Then the K_2CO_3 (0.4 mmol, 2.0 equiv.) and diaryl disulfide (0.4 mmol, 2.0 equiv.) was added under argon atmosphere. The tube stirred at ambient temperature for 6 hours in the dark. Upon completion, the reaction mixture was carefully concentrated and the residue was further purified by flash chromatography to give the desired disulfide products.

General Procedure for the Diversification via in-situ Trapping

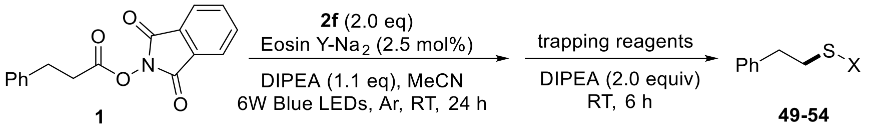

To an oven-dried 10-ml Schlenk tube equipped with a magnetic stir bar and a Teflon coated septum screwcap was added the NHPI redox-active ester **1** (0.2 mmol, 1.0 equiv.), 4-methoxythiobenzamide (**2f**, 0.4 mmol, 2.0 equiv.), eosin Y (2.5 mol%). The tube was evacuated and back-filled with argon for three cycles. The DIPEA (0.22 mmol, 1.1 equiv.) and dry CH_3CN (2.0 ml) was added via a gastight syringe under argon atmosphere. Make sure the screwcap was closed, the solvent was frozen by liquid nitrogen. Then the screwcap was opened and the tube was evacuated for about 3 mins. The screwcap was closed and let the solvent melts in a tepid water bath. Repeat above Freeze-Pump-Thaw procedures for 3-5 times until you no longer see the evolution of gas as the solution thaws. The tube was filled with argon and sealed, irradiated with 6W blue LED reactor and stirred at ambient temperature for 24 hours. Then the DIPEA (0.4 mmol, 2.0 equiv.) and the corresponding trapping reagent (1.0 -4.0 equiv.) was added under argon atmosphere. The tube stirred at ambient temperature for 6 hours in the dark. Upon completion, the reaction mixture was carefully concentrated and the residue was further purified by flash chromatography.

Characterizations of products

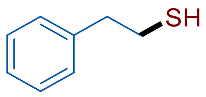

3

Corresponding NHPI ester (0.2 mmol) was thiolated according to the general procedure. The crude residue was purified by flash column chromatography (SiO_2; pentane) to afford thiol **3** (19.3 mg, 70%) as a colorless oil. (88% GC yield using Anisole as internal standard)

^{1}H NMR (400 MHz, $CDCl_3$): δ (ppm) 7.33-7.19 (m, 5H, Ar-H), 2.92 (t, *J* = 7.1 Hz, 2H, Ph-CH_2), 2.82-2.76 (m, 2H, CH_2S), 1.38 (t, *J* = 7.5 Hz, 1H, SH).

^{13}C NMR (100 MHz, $CDCl_3$): δ (ppm) 139.8, 128.6, 128.5, 126.5, 40.2, 26.0.

HRMS (APCI): m/z calculated for $C_8H_{11}S[M+H]^+$: 139.0576, found 139.0577.

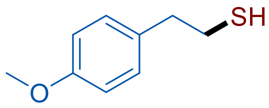

4

Corresponding NHPI ester (0.2 mmol) was thiolated according to the general procedure. The crude residue was purified by flash column chromatography (SiO_2; pentane) to afford thiol **4** (17.5 mg, 52%) as a colorless oil.

^{1}H NMR (400 MHz, $CDCl_3$): δ (ppm) 7.11 (d, *J* = 7.2 Hz, 2H, Ar-H), 6.85 (d, *J* = 7.0 Hz, 2H, Ar-H), 3.79 (s, 3H, ArOCH_3), 2.87 (t, *J* = 7.3 Hz, 2H, Ar-CH_2), 2.78-2.73 (m, 2H, ArCH_2<u>CH_2</u>SH), 1.36 (t, *J* = 7.9 Hz, 1H, SH).

^{13}C NMR (100 MHz, $CDCl_3$): δ (ppm) 158.3, 131.9, 129.6, 113.9, 55.2, 39.3, 26.3.

HRMS (APCI): m/z calculated for $C_9H_{12}OS[M+H]^+$: 169.0682, found 169.0682.

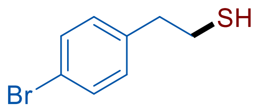

5

Corresponding NHPI ester (0.2 mmol) was thiolated according to the general procedure. The crude residue was purified by flash column chromatography (SiO_2; pentane) to afford thiol **5**

(25.5 mg, 59%) as a colorless oil.

^{1}H NMR (400 MHz, CDCl$_3$): δ (ppm) 7.43 (d, *J* = 7.9 Hz, 2H, Ar-H), 7.07 (d, *J* = 7.6 Hz, 2H, Ar-H), 2.88 (t, *J* = 6.3 Hz, 2H, Ar-CH$_2$), 2.79-2.74 (m, 2H, CH$_2$S), 1.36 (t, *J* = 7.4 Hz, 1H, SH).

^{13}C NMR (100 MHz, CDCl$_3$): δ (ppm) 138.7, 131.6, 130.4, 120.4, 39.5, 25.8.

HRMS (ESI): m/z calculated for $C_8H_8BrS[M-H]^-$: 214.9536, found 214.9536.

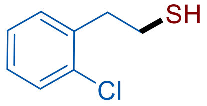

6

Corresponding NHPI ester (0.2 mmol) was thiolated according to the general procedure. The crude residue was purified by flash column chromatography (SiO$_2$; pentane) to afford thiol **6** (18.9 mg, 55%) as a colorless oil.

^{1}H NMR (400 MHz, CDCl$_3$): δ (ppm) 7.35 (d, *J* = 7.3 Hz, 1H, Ar-H), 7.25-7.16 (m, 3H, Ar-H), 3.04 (t, *J* = 7.4 Hz, 2H, ArCH$_2$), 2.83-2.78 (m, 2H, SCH$_2$), 1.41 (t, *J* = 8.0 Hz, 1H, SH).

^{13}C NMR (100 MHz, CDCl$_3$): δ (ppm) 137.4, 134.0, 131.0, 129.6, 128.0, 126.8, 38.2, 24.1.

HRMS (APCI): m/z calculated for $C_8H_{10}ClS[M+H]^+$: 173.0186, found 173.0186.

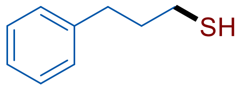

7

Corresponding NHPI ester (0.2 mmol) was thiolated according to the general procedure. The crude residue was purified by flash column chromatography (SiO$_2$; pentane) to afford thiol **7** (22.8 mg, 75%) as a colorless oil.

^{1}H NMR (400 MHz, CDCl$_3$): δ (ppm) 7.30-7.25 (m, 2H, Ar-H), 7.21-7.17 (m, 3H, Ar-H), 2.73 (t, *J* = 7.5 Hz, 2H, ArCH$_2$), 2.56-2.61 (m, *J* = 7.4 Hz, 2H, $\underline{CH_2}$SH),

1.97-1.90 (m, 2H, $ArCH_2\underline{CH_2}CH_2SH$), 1.35 (t, J = 7.8 Hz, 1H, SH).

^{13}C NMR (100 MHz, $CDCl_3$): δ (ppm) 141.3, 128.4, 128.4,126.0, 35.5, 34.4, 24.0.

HRMS (ESI): m/z calculated for $C_9H_{13}S$ $(M+H)^+$: 153.0732, found 153.0732.

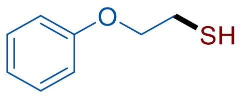

8

Corresponding NHPI ester (0.2 mmol) was thiolated according to the general procedure. The crude residue was purified by flash column chromatography (SiO_2; pentane) to afford thiol **8** (10.8 mg, 35%) as a colorless oil.

^{1}H NMR (400 MHz, $CDCl_3$): δ (ppm) 7.28 (m, 2H, ArH), 6.98-6.90 (m, 3H, Ar-H), 4.25 (t, J = 6.2 Hz, 2H, OCH_2), 3.09 (t, J = 6.4 Hz, 2H, CH_2S).

^{13}C NMR (100 MHz, $CDCl_3$): δ (ppm) 158.3, 129.5, 121.1, 114.7, 66.0, 37.9.

HRMS (APCI): m/z calculated for $C_8H_{11}OS[M+H]^+$: 155.0525, found 155.0525.

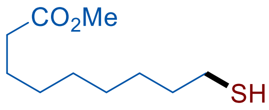

9

Corresponding NHPI ester (0.2 mmol) was thiolated according to the general procedure. The crude residue was purified by flash column chromatography (SiO_2; petroleum ether / EtOAc = 20:1) to afford thiol **9** (33.1 mg, 81%) as a colorless oil.

^{1}H NMR (400 MHz, $CDCl_3$): δ (ppm) 3.67 (s, 3H, OCH_3), 2.55-2.49 (m, 2H, $\underline{CH_2}S$), 2.30 (t, J = 7.2 Hz, 2H, $CH_2C{=}O$), 1.64-1.57 (m, 4H, $\underline{CH_2}CH_2C{=}O$, $\underline{CH_2}$ CH_2S), 1.38-1.31 (m, 9H, $4{\times}CH_2$ + SH).

^{13}C NMR (100 MHz, $CDCl_3$): δ (ppm) 174.2, 51.4, 34.0, 33.9, 29.1, 29.0, 28.8, 28.2, 24.9, 24.6.

HRMS (APCI): m/z calculated for $C_{10}H_{21}O_2S[M+H]^+$: 205.1257, found 205.1257.

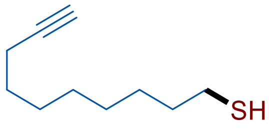

10

Corresponding NHPI ester (0.2 mmol) was thiolated according to the general procedure. The crude residue was purified by flash column chromatography (SiO_2; petroleum ether) to afford thiol **10** (10.9 mg, 32%) as a colorless oil.

^{1}H NMR (400 MHz, $CDCl_3$): δ (ppm) 2.55-2.50 (m, 2H, CH_2S), 2.18 (t, *J* = 6.9 Hz, 2H, $CH_2C≡C$), 1.94 (s, 1H, C≡CH), 1.64-1.59 (m, 2H, $SCH_2\underline{CH_2}$), 1.54-1.49 (m, 2H, $\underline{CH_2}CH_2C≡C$), 1.39-1.31 (m, 9H, 4×CH_2 + SH).

^{13}C NMR (100 MHz, $CDCl_3$): δ (ppm) 84.7, 68.1, 34.0, 28.9, 28.9, 28.6, 28.4, 28.3, 24.6, 18.4.

HRMS (APCI): m/z calculated for $C_{10}H_{19}S[M+H]^+$: 171.1201, found 171.1201.

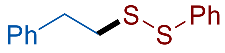

11

Corresponding NHPI ester (0.2 mmol) was sulfurized according to the general procedure. The crude residue was purified by flash column chromatography (SiO_2; petroleum ether) to afford disulfide **11** (35.4 mg, 72%) as a colorless oil.

^{1}H NMR (400 MHz, $CDCl_3$): δ (ppm) 7.54 (d, *J* = 7.7 Hz, 2H, ArH), 7.33-7.20 (m, 6H, ArH), 7.15 (d, *J* = 7.3 Hz, 2H, ArH), 2.98 (s, 4H, CH_2CH_2).

^{13}C NMR (100 MHz, $CDCl_3$): δ (ppm) 139.8, 137.4, 129.0, 128.6, 128.5, 127.6, 126.8, 126.4, 40.0, 35.3.

HRMS (APCI): m/z calculated for $C_{14}H_{15}S_2[M+H]^+$: 247.0610, found 247.0610.

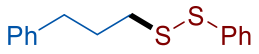

12

Corresponding NHPI ester (0.2 mmol) was sulfurized according to the general procedure. The crude residue was purified by flash column chromatography (SiO_2; petroleum ether) to afford disulfide **12** (16.9 mg, 65%) as a colorless oil.

^{1}H NMR (400 MHz, $CDCl_3$): δ (ppm) 7.53-7.51 (m, 2H, Ar-H), 7.33-7.10 (m, 8H, Ar-H), 2.75-2.67 (m , 4H, $PhCH_2$, SCH_2), 2.04-1.97 (m, 2H,$CH_2\underline{CH_2}CH_2$).

^{13}C NMR (100 MHz, $CDCl_3$): δ (ppm) 141.2, 137.5, 128.9, 128.4, 128.4, 127.7, 126.8, 125.9, 38.0, 34.3, 30.1.

HRMS (APCI): m/z calculated for $C_9H_{11}S[M]^{\cdot+}$: 260.0688, found 260.0688.

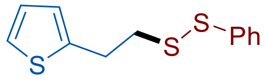

13

Corresponding NHPI ester (0.2 mmol) was sulfurized according to the general procedure. The crude residue was purified by flash column chromatography (SiO_2; petroleum ether) to afford disulfide **13** (20.7 mg, 41%) as a colorless oil.

^{1}H NMR (400 MHz, $CDCl_3$): δ (ppm) 7.54 (d, J = 7.2 Hz, 2H, Ar-H), 7.32 (t, J = 7.1 Hz, 2H, Ar-H), 7.22 (t, J = 7.1 Hz, 1H, Ar-H), 7.13 (s, 1H, Thiophene Ar-H), 6.91 (s, 1H, Thiophene Ar-H), 6.79 (s, 1H, Thiophene Ar-H), 3.20 (t, J = 6.9 Hz, 2H, Thiophene-CH_2), 3.00 (t, J = 6.8 Hz, 2H, CH_2S).

^{13}C NMR (100 MHz, $CDCl_3$): δ (ppm) 142.2, 137.2, 129.0, 127.8, 126.9, 126.8, 125.1, 123.7, 40.0, 29.4.

HRMS (APCI): m/z calculated for $C_{12}H_{13}S_3[M+H]^+$: 253.0174, found 253.0174.

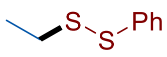

14

Corresponding NHPI ester (0.2 mmol) was sulfurized according to the general procedure. The crude residue was purified by flash column chromatography (SiO_2; petroleum ether) to afford disulfide **14** (13.6 mg, 40%) as a colorless oil.

^{1}H NMR (400 MHz, $CDCl_3$): δ (ppm) 7.54 (d, *J* = 7.0 Hz, 2H, Ar-H), 7.32 (t, *J* = 7.3 Hz, 2H, Ar-H), 7.23 (t, *J* = 7.2 Hz, 1H, Ar-H), 2.75 (q, *J* = 7.1 Hz, 2H, S$\underline{CH_2}$$CH_3$), 1.31 (t, *J* = 6.8 Hz, 3H, $SCH_2$$\underline{CH_3}$).

^{13}C NMR (100 MHz, $CDCl_3$): δ (ppm) 137.7, 128.9, 127.4, 126.6, 32.7, 14.1.

HRMS (ESI): m/z calculated for $C_8H_{11}S_2[M+H]^+$: 171.0297, found 171.0297.

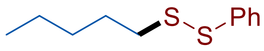

15

Corresponding NHPI ester (0.2 mmol) was sulfurized according to the general procedure. The crude residue was purified by flash column chromatography (SiO_2; petroleum ether) to afford disulfide **15** (33.9 mg, 80%) as a colorless oil.

^{1}H NMR (400 MHz, $CDCl_3$): δ (ppm) 7.53 (d, *J* = 7.6 Hz, 2H, Ar-H), 7.31 (t, *J* = 7.3 Hz, 2H, Ar-H), 7.20 (t, *J* = 7.3 Hz, 1H, Ar-H), 2.73 (t, *J* = 7.3 Hz, 2H, SCH_2), 1.70-1.63 (m, 2H, $SCH_2$$\underline{CH_2}$), 1.38-1.25 (m, 4H, 2×$CH_2$), 0.87 (t, *J* = 6.8 Hz, 3H, CH_3).

^{13}C NMR (100 MHz, $CDCl_3$): δ (ppm) 137.7, 128.9, 127.4, 126.6, 39.0, 30.6, 28.5, 22.2, 13.9.

HRMS (APCI): m/z calculated for $C_{16}H_{17}S_2[M+H]^+$: 213.0766, found 213.0766.

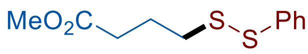

16

Corresponding NHPI ester (0.2 mmol) was sulfurized according to the general procedure. The crude residue was purified by flash column chromatography (SiO_2; petroleum ether/EtOAc = 60:1) to afford disulfide **16** (34.9 mg, 72%) as a colorless oil.

^{1}H NMR (400 MHz, CDCl$_3$): δ (ppm) 7.53 (d, J = 7.4 Hz, 2H, Ar-H), 7.32 (t, J = 7.0 Hz, 2H, Ar-H), 7.22 (t, J = 7.2 Hz, 1H, Ar-H), 3.65 (s, 3H, OMe), 2.77 (t, J = 6.3 Hz, 2H, CH$_2$-S), 2.41 (t, J = 6.4 Hz, 2H, CH$_2$C=O), 2.03-1.98 (m, 2H, CH$_2$<u>CH$_2$</u>CH$_2$).

^{13}C NMR (100 MHz, CDCl$_3$): δ (ppm) 173.2, 137.2, 129.0, 127.8, 126.9, 51.6, 37.7, 32.3, 23.8.

HRMS (APCI): m/z calculated for $C_{11}H_{14}O_2S_2[M+H]^+$: 243.0508, found 243.0508.

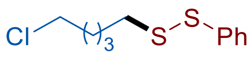

17

Corresponding NHPI ester (0.2 mmol) was sulfurized according to the general procedure. The crude residue was purified by flash column chromatography (SiO$_2$; petroleum ether) to afford disulfide **17** (22.1 mg, 45%) as a colorless oil.

^{1}H NMR (400 MHz, CDCl$_3$): δ (ppm) 7.53 (d, J = 7.4 Hz, 2H, Ar-H), 7.32 (t, J = 7.3 Hz, 2H, Ar-H), 7.22 (t, J = 7.3 Hz, 1H, Ar-H), 3.49 (t, J = 6.1 Hz, 2H, ClCH$_2$), 2.74 (t, J = 6.8 Hz, 2H, CH$_2$S),1.77-1.66 (m, 4H, 2×CH$_2$), 1.53-1.47 (m, 2H, ClCH$_2$CH$_2$<u>CH$_2$</u>).

^{13}C NMR (100 MHz, CDCl$_3$): δ (ppm) 137.5, 129.0, 127.6, 126.8, 44.7, 38.6, 32.1, 28.0, 25.7.

HRMS (APCI): m/z calculated for $C_{11}H_{16}ClS_2[M+H]^+$: 247.0376, found 247.0376.

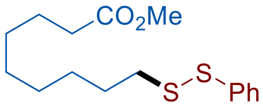

18

Corresponding NHPI ester (0.2 mmol) was sulfurized according to the general procedure. The crude residue was purified by flash column chromatography (SiO$_2$; petroleum ether / EtOAc = 50:1) to afford disulfide **18** (43.7 mg, 70%) as a colorless oil.

^{1}H NMR (400 MHz, CDCl$_3$): δ (ppm) 7.53 (d, J = 7.5 Hz, 2H, Ar-H), 7.31 (t, J = 7.4 Hz, 2H, Ar-H), 7.21 (t, J = 7.3 Hz, 1H, Ar-H), 3.66 (s, 3H, OCH$_3$), 2.73 (t, J = 7.2 Hz, 2H, SCH$_2$), 2.29 (t, J = 7.4 Hz, 2H, O=CCH$_2$), 1.69-1.57 (m, 4H, SCH$_2$<u>CH$_2$</u>, O=CCH$_2$<u>CH$_2$</u>), 1.36-1.26 (m, 8H, 4×CH$_2$).

^{13}C NMR (100 MHz, CDCl$_3$): δ (ppm) 174.2, 137.6, 128.9, 127.4, 126.6, 51.4, 38.9, 34.0, 29.0, 29.0, 28.9, 28.7, 28.3, 24.9.

HRMS (APCI): m/z calculated for $C_{16}H_{25}O_2S_2$[M+H]$^+$: 313.1290, found 313.1290.

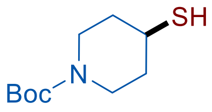

19

Corresponding NHPI ester (0.2 mmol) was thiolated according to the general procedure. The crude residue was purified by flash column chromatography (SiO$_2$; petroleum ether/EtOAc = 9: 1) to afford thiol **19** (17.4 mg, 40%) as a colorless oil.

^{1}H NMR (400 MHz, CDCl$_3$): δ (ppm) 4.02 (d, *J* = 9.6 Hz, 2H, 2×C$\underline{H}^a$H^{b}N), 2.90-2.80 (m, 3H, 2×CH$^a\underline{H}^b$N + CHS), 1.98 (d, *J* = 12.9 Hz, 2H, 2×CH$^a\underline{H}^b$CH), 1.56-1.46 (m, 12H, 2× C$\underline{H}^a$H^{b}CH + SH + $(CH_3)_3$C).

^{13}C NMR (100 MHz, CDCl$_3$): δ (ppm) 154.6, 79.7, 47.4, 43.4, 31.7, 28.4.

HRMS (APCI): m/z calculated for $C_{10}H_{20}NO_2S$[M+H]$^+$: 218.1209, found 218.1209.

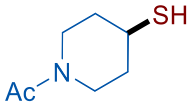

20

Corresponding NHPI ester (0.2 mmol) was thiolated according to the general procedure. The crude residue was purified by flash column chromatography (SiO$_2$; petroleum ether) to afford thiol **20** (11.1 mg, 35%) as a colorless oil.

^{1}H NMR (400 MHz, CDCl$_3$): δ (ppm) 4.38 (d, *J* = 13.5 Hz, 1H, NC$\underline{H}^a$H^b), 3.78 (d, *J* = 13.8 Hz, 1H, NC$\underline{H}^c$H^d), 3.14 (t, *J* = 12.4 Hz, 1H, NCH$^a\underline{H}^b$), 3.06-2.97 (m, 1H, CH), 2.84 (t, *J* = 12.1 Hz, 1H, NCH$^c\underline{H}^d$), 2.09-2.00 (m, 5H, O=CCH$_3$ + 2×C$\underline{H}^a$H^b), 1.59-1.49 (m, 3H, 2×CH$^a\underline{H}^b$ + SH).

^{13}C NMR (100 MHz, CDCl$_3$): δ (ppm) 168.8, 45.9, 41.1, 36.9, 36.1, 35.8, 21.4.

HRMS (APCI): m/z calculated for $C_7H_{14}NOS$[M+H]$^+$: 160.0791, found 160.0791.

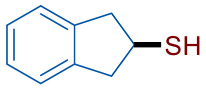

21

Corresponding NHPI ester (0.2 mmol) was thiolated according to the general procedure. The crude residue was purified by flash column chromatography (SiO_2; petroleum ether) to afford thiol **21** (10.2 mg, 34%) as a colorless oil.

^{1}H NMR (400 MHz, CDCl$_3$): δ (ppm) 7.20-7.17 (m, 4H, Ar-H), 3.75-3.67 (m, 1H, CH), 3.41-3.36 (dd, J = 15.6, 6.8 Hz, 2H, 2×C$\underline{H_a}$H$_b$), 2.95-2.90 (dd, J = 15.4, 3.8 Hz, 2H, 2×CH$_a\underline{H_b}$), 1.83 (d, J = 4.8 Hz, 1H, SH).

^{13}C NMR (100 MHz, CDCl$_3$): δ (ppm) 141.6, 126.7, 124.4, 44.3, 37.9.

HRMS (APCI): m/z calculated for $C_9H_{11}S[M+H]^+$: 151.0576, found 151.0577.

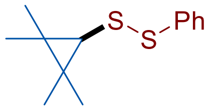

22

Corresponding NHPI ester (0.2 mmol) was sulfurized according to the general procedure. The crude residue was purified by flash column chromatography (SiO_2; petroleum ether) to afford disulfide **22** (11.4 mg, 24%) as a colorless oil.

^{1}H NMR (400 MHz, CDCl$_3$): δ (ppm) 7.57 (d, J = 7.0 Hz, 2H, Ar-H), 7.32 (t, J = 7.0 Hz, 2H, Ar-H), 7.23 (t, J = 7.2 Hz, 1H, Ar-H), 1.88 (s, 1H, SCH), 1.11 (s, 6H, 2×CH$_3$), 1.08 (s, 6H, 2×CH$_3$).

^{13}C NMR (100 MHz, CDCl$_3$): δ (ppm) 138.0, 129.0, 128.9, 127.0, 42.6, 27.4, 23.2, 17.6.

HRMS (ESI): m/z calculated for $C_{13}H_{19}S_2[M+H]^+$: 239.0923, found 239.0923.

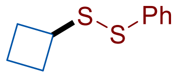

23

Corresponding NHPI ester (0.2 mmol) was sulfurized according to the general procedure. The crude residue was purified by flash column chromatography (SiO_2; petroleum ether) to afford disulfide **23** (23.5 mg, 60%) as a colorless oil.

^{1}H NMR (400 MHz, CDCl$_3$): δ (ppm) 7.52 (d, J = 7.4 Hz, 2H, Ar-H), 7.30 (t, J = 6.8 Hz, 2H, Ar-H), 7.19 (t, J =7.4 Hz, 1H, Ar-H), 3.68-3.60 (m, 1H, CH-S), 2.25-2.23 (m, 2H, 2×C$\underline{H}^a$H^{b}S), 2.17-2.08 (m, 2H, CH$^a\underline{H}^b$S), 1.91-1.85 (m, 2H, $\underline{CH_2}$-CH$_2$-CH).

^{13}C NMR (100 MHz, CDCl$_3$): δ (ppm) 138.2, 128.8, 127.0, 126.4, 44.5, 29.7, 18.1.

HRMS (APCI): m/z calculated for $C_{10}H_{13}S_2$[M+H]$^+$: 197.0453, found 197.0453.

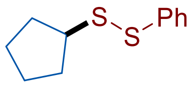

24

Corresponding NHPI ester (0.2 mmol) was sulfurized according to the general procedure. The crude residue was purified by flash column chromatography (SiO$_2$; petroleum ether) to afford disulfide **23** (27.3 mg, 65%) as a colorless oil.

^{1}H NMR (400 MHz, CDCl$_3$): δ (ppm) 7.54 (d, J = 8.0 Hz, 2H, Ar-H), 7.31 (t, J = 7.6 Hz, 2H, Ar-H), 7.20 (t, J = 7.3 Hz, 1H, Ar-H), 3.37-3.30 (m, 1H, SCH), 1.96-1.90 (m, 2H, 2×C$\underline{H}^a$H^b), 1.18-1.55 (m, 6H, 2×CH$^a\underline{H}^b$ + 2×CH$_2$).

^{13}C NMR (101 MHz, CDCl$_3$): δ (ppm) 138.0, 128.8, 127.1, 126.5, 50.3, 32.8, 24.7.

HRMS (APCI): m/z calculated for $C_{11}H_{15}S_2$ (M+H)$^+$: 211.0610, found 211.0610.

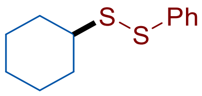

25

Corresponding NHPI ester (0.2 mmol) was sulfurized according to the general procedure. The crude residue was purified by flash column chromatography (SiO$_2$; petroleum ether) to afford disulfide **26** (23.7 mg, 53%) as a colorless oil.

^{1}H NMR (400 MHz, CDCl$_3$): δ (ppm) 7.53 (d, J = 7.4 Hz, 2H, Ar-H), 7.30 (t, J = 7.4 Hz, 2H, Ar-H), 7.15 (d, J = 7.3 Hz, 1H, Ar-H), 2.83-2.77 (m, 1H, SCH), 2.03-2.00 (m, 2H, CH$_2$), 1.77-1.74 (m, 2H, CH$_2$), 1.60-1.54 (m, 1H, CH$_2$), 1.41-1.18 (m, 5H, CH$_2$).

^{13}C NMR (101 MHz, CDCl$_3$): δ (ppm) 138.4, 128.8, 126.8, 126.3, 49.8, 32.6, 26.0, 25.6.

HRMS (EI): m/z calculated for $C_{12}H_{17}S_2$ [M+H]$^+$: 225.0766 found 225.0765.

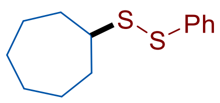

26

Corresponding NHPI ester (0.2 mmol) was sulfurized according to the general procedure The crude residue was purified by flash column chromatography (SiO_2; petroleum ether) to afford disulfide **26** (16.7 mg, 35%) as a colorless oil.

^{1}H NMR (400 MHz, $CDCl_3$): δ (ppm) 7.57 (d, J = 7.2 Hz, 2H, Ar-H), 7.35 (t, J = 7.1 Hz, 2H, Ar-H), 7.23 (t, J = 7.2 Hz, 1H, Ar-H), 3.05-2.98 (m, 1H, CHS), 2.14-2.09 (m, 2H, 2×C$\underline{H}^{a}$$H^{b}$), 1.78-1.42 (m, 10H, 2×C$H^{a}$$\underline{H}^{b}$ + 4×CH_2).

^{13}C NMR (100 MHz, $CDCl_3$): δ (ppm) 138.1, 128.8, 126.9, 126.3, 51.4, 33.9, 28.3, 25.8.

HRMS (APCI): m/z calculated for $C_{13}H_{19}S_2$[M+H]$^+$: 239.0923, found 239.0923.

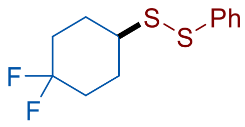

27

Corresponding NHPI ester (0.2 mmol) was sulfurized according to the general procedure. The crude residue was purified by flash column chromatography (SiO_2; petroleum ether) to afford disulfide **27** (38.0 mg, 73%) as a colorless oil.

^{1}H NMR (400 MHz, $CDCl_3$): δ (ppm) 7.53 (d, J = 7.4 Hz, 2H, Ar-H), 7.32 (t, J = 7.1 Hz, 2H, Ar-H), 7.22 (t, J = 7.3 Hz, 1H, Ar-H), 2.93 (s, 1H, SCH), 2.15-2.05 (m, 4H, 2×C$\underline{H}^{a}$$H^{b}$ + 2×C$\underline{H}^{c}$$H^{d}$), 1.84-1.70 (m, 4H, 2×C$H^{a}$$\underline{H}^{b}$ + 2×C$H^{c}$$\underline{H}^{d}$).

^{13}C NMR (101 MHz, $CDCl_3$): δ (ppm) 137.5, 129.0, 127.3, 126.8, 122.5(t, J_{C-F} = 242.3 Hz), 46.5, 32.3(t, J_{C-F} = 97.6 Hz), 28.0(t, J_{C-F} = 19.8 Hz).

^{19}F NMR (376 MHz, $CDCl_3$): δ (ppm) 95.7 (d, J = 237.1 Hz, 1F), 98.8 (d, J = 236.3 Hz, 1F).

HRMS (EI): m/z calculated for $C_{12}H_{15}F_2S_2$ [M+H]$^+$: 261.0578, found 261.0578.

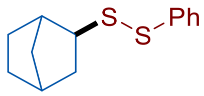

28

Corresponding NHPI ester (0.2 mmol) was sulfurized according to the general procedure. The crude residue was purified by flash column chromatography (SiO_2; petroleum ether) to afford disulfide **28** (30.2 mg, 64%) as a colorless oil.

^{1}H NMR (400 MHz, $CDCl_3$): δ (ppm) 7.53 (d, *J* = 7.4 Hz, 2H, Ar-H), 7.31 (t, *J* = 7.1 Hz, 2H, Ar-H), 7.19 (t, *J* = 7.3 Hz, 1H, Ar-H), 2.93-2.90 (m, 1H, CHS), 2.39 (d, *J* = 2.9 Hz, 1H), 2.29 (s, 1H, CH), 1.69-1.56 (m, 3H), 1.49-1.45 (m, 1H), 1.38-1.34 (m, 1H), 1.16-1.08 (m, 3H).

^{13}C NMR (100 MHz, $CDCl_3$): δ (ppm) 137.9, 128.8, 127.0, 126.4, 53.0, 42.1, 37.8, 36.9, 35.2, 28.7, 28.7.

HRMS (APCI): m/z calculated for $C_{13}H_{17}S_2[M+H]^+$: 237.0766, found 237.0766.

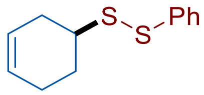

29

Corresponding NHPI ester (0.2 mmol) was sulfurized according to the general procedure. The crude residue was purified by flash column chromatography (SiO_2; petroleum ether) to afford disulfide **29** (22.2 mg, 50%) as a colorless oil.

^{1}H NMR (400 MHz, $CDCl_3$): δ (ppm) 7.55 (d, *J* = 6.8 Hz, 2H, Ar-H), 7.31 (t, *J* = 7.1 Hz, 2H, Ar-H), 7.20 (t, *J* = 7.9 Hz, 1H, Ar-H), 5.68-5.59 (m, 2H, CH=CH), 3.11-3.06 (m, 1H, CHS), 2.43-2.39 (m, 1H), 2.19-2.04 (m, 4H), 1.75-1.66 (m, 1H).

^{13}C NMR (100 MHz, $CDCl_3$): δ (ppm) 138.2, 128.9, 127.1, 126.8, 126.5, 125.0, 46.0, 31.1, 28.1, 24.7.

HRMS (ESI): m/z calculated for $C_{12}H_{15}S_2[M+H]^+$: 223.0610, found 223.0611.

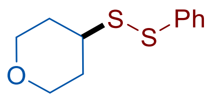

30

Corresponding NHPI ester (0.2 mmol) was sulfurized according to the general procedure. The crude residue was purified by flash column chromatography (SiO_2; petroleum ether / EtOAc = 20:1) to afford disulfide **30** (24.4 mg, 54%) as a colorless oil.

^{1}H NMR (400 MHz, $CDCl_3$): δ (ppm) 7.54 (d, J = 7.6 Hz, 2H, Ar-H), 7.31 (t, J = 7.2 Hz, 2H, Ar-H), 7.21 (t , J = 7.3 Hz, 1H, ArH), 3.96 (d, J = 11.5 Hz, 2H, 2×C$\underline{H}^{a}H^{b}$O), 3.38 (t, J = 11.3 Hz, 2H, 2×C$H^{a}\underline{H}^{b}$O), 3.04-2.96 (m, 1H, CH-S), 1.93 (d, J = 12.7 Hz, 2H, 2×C$\underline{H}^{c}H^{d}$CHS), 1.74-1.61 (m, 2H, 2×C$H^{c}\underline{H}^{d}$CHS).

^{13}C NMR (100 MHz, $CDCl_3$): δ (ppm) 137.9, 128.9, 127.1, 126.6, 67.3, 46.2, 32.4.

HRMS (APCI): m/z calculated for $C_{11}H_{14}OS_2$[M+H]$^+$: 227.0559, found 227.0559.

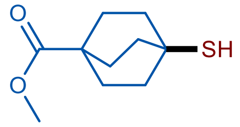

31

Corresponding NHPI ester (0.2 mmol) was thiolated according to the general procedure. The crude residue was purified by flash column chromatography (SiO_2; petroleum ether) to afford thiol **31** (22.4 mg, 56%) as a colorless oil.

^{1}H NMR (600 MHz, $CDCl_3$): δ (ppm) 3.63 (s, 3H, CH_3), 1.85 (s, 12H, 6×CH_2), 1.58 (s, 1H, SH).

^{13}C NMR (151 MHz, $CDCl_3$): δ (ppm) 177.65, 51.76, 40.67, 37.58, 35.68, 29.59.

HRMS (ESI): m/z calculated for $C_{10}H_{17}O_2S$[M+H]$^+$: 201.0944, found 201.0941.

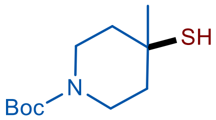

32

Corresponding NHPI ester (0.2 mmol) was thiolated according to the general procedure. The crude residue was purified by flash column chromatography (SiO_2; petroleum ether) to afford

thiol **32** (7.4 mg, 16%) as a colorless oil.

^{1}H NMR (600 MHz, CDCl$_3$): δ (ppm) 3.78 (s, 2H, 2×NC$\underline{H}^a$H^b), 3.28 – 3.16 (m, 2H, 2×NC$\underline{H}^b$H^a), 1.59 (s, 1H, SH), 1.59 (s, 4H, 2×CCH$_2$), 1.46 (s, 3H, CCH$_3$), 1.45 (s, 9H, C(CH$_3$)$_3$).

^{13}C NMR (151 MHz, CDCl$_3$): δ (ppm) 154.72, 79.53, 43.84, 33.73, 29.69, 28.43.

HRMS (ESI): m/z calculated for $C_{11}H_{22}NO_2S[M+H]^+$: 232.1366, found 232.1366.

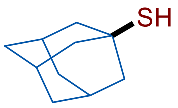

33

Corresponding NHPI ester (0.2 mmol) was thiolated according to the general procedure. The crude residue was purified by flash column chromatography (SiO$_2$; pentane) to afford thiol **31** (17.5 mg, 52%) as a white solid.

^{1}H NMR (400 MHz, CDCl$_3$): 2.02 (s, 3H), 1.93 (s, 6H), 1.70-1.64 (m, 7H, 3×CH$_2$ + 1×SH).

^{13}C NMR (100 MHz, CDCl$_3$): δ (ppm) 47.6, 43.3, 35.8, 30.1.

HRMS (EI): m/z calculated for $C_{10}H_{17}S$ $[M+H]^+$: 169.1045, found 169.1041.

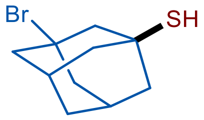

34

Corresponding NHPI ester (0.2 mmol) was sulfurized according to the general procedure. The crude residue was purified by flash column chromatography (SiO$_2$; petroleum ether) to afford disulfide **34** (29.5 mg, 60%) as a white solid.

^{1}H NMR (600 MHz, CDCl$_3$): δ (ppm) 2.51 – 2.48 (m, 2H, 2×CH), 2.29 – 2.21 (m, 4H, 2×BrCCH$_2$), 2.21 – 2.18 (m, 2H, BrCCH$_2$), 1.94 – 1.86 (m, 4H, 2×CH$_2$), 1.79 (s, 1H, SH), 1.66 – 1.63 (m, 2H, CH$_2$).

^{13}C NMR (151 MHz, CDCl$_3$): δ (ppm) 63.49, 58.23, 47.32, 45.27, 44.97, 33.77, 33.40.

HRMS (ESI): m/z calculated for $C_{10}H_{16}BrS[M+H]^+$: 246.0151, found 246.0151.

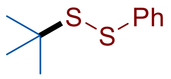

35

Corresponding NHPI ester (0.2 mmol) was sulfurized according to the general procedure, using CF_3CH_2OH as solvent instead of CH_3CN.The crude residue was purified by flash column chromatography (SiO_2; petroleum ether) to afford disulfide **35** (9.9 mg, 25%) as a colorless oil.

^{1}H NMR (400 MHz, $CDCl_3$): δ (ppm) 7.56 (d, *J* = 7.6 Hz, 2H, Ar-H), 7.29 (t, *J* = 7.2 Hz, 2H, Ar-H), 7.17 (t, *J* = 7.3 Hz, 1H, Ar-H),1.31 (s, 9H, 3×CH_3).

^{13}C NMR (100 MHz, $CDCl_3$): δ (ppm) 138.8, 128.7, 126.8, 126.2, 49.2, 29.8.

HRMS (APCI): m/z calculated for $C_{10}H_{15}S_2$[M+H]$^+$: 199.0610, found 199.0610.

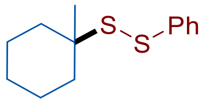

36

Corresponding NHPI ester (0.2 mmol) was sulfurized according to the general procedure, using CF_3CH_2OH as solvent instead of CH_3CN. The crude residue was purified by flash column chromatography (SiO_2; petroleum ether) to afford disulfide **36** (18.6 mg, 39%) as a colorless oil.

^{1}H NMR (400 MHz, $CDCl_3$): δ (ppm) 7.57 (d, *J* = 7.4 Hz, 2H, Ar-H), 7.28 (t, *J* = 7.5 Hz, 2H, Ar-H), 7.17 (t, *J* = 7.2 Hz, 1H, Ar-H), 1.77-1.27 (m, 13H, 5×CH_2 + CH_3).

^{13}C NMR (101 MHz, $CDCl_3$): δ (ppm) 138.9, 128.6, 127.2, 126.2, 53.2, 37.3, 27.4, 25.6, 22.6.

HRMS (APCI): m/z calculated for $C_{13}H_{19}S_2$ (M+H)$^+$: 239.0923, found 239.0923.

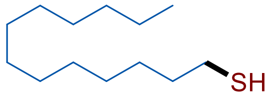

37

Corresponding NHPI ester (0.2 mmol) was thiolated according to the general procedure. The crude residue was purified by flash column chromatography (SiO2; petroleum ether) to afford thiol **37** (32.0 mg, 74%) as a white solid.

^{1}H NMR (400 MHz, $CDCl_3$): δ (ppm) 2.52 (t, *J* = 7.2 Hz, 2H, <u>CH_2</u>SH), 1.64-1.59 (m, 2H, <u>CH_2</u>CH_2SH), 1.37-1.26 (m, 21H, 10×CH_2, 1×SH), 0.88 (t, *J* = 6.0 Hz, 3H, <u>CH_3</u>CH_2).

^{13}C NMR (100 MHz, $CDCl_3$): δ (ppm) 34.1, 31.9, 29.7, 29.66(2C), 29.64, 29.5, 29.3, 29.1, 28.4, 24.6, 22.9, 14.1.

HRMS (ESI): m/z calculated for $C_{13}H_{29}S[M+H]^+$: 217.1984, found 217.1985.

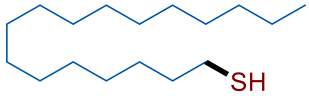

38

Corresponding NHPI ester (0.2 mmol) was thiolated according to the general procedure. The crude residue was purified by flash column chromatography (SiO_2; petroleum ether) to afford thiol **38** (39.2 mg, 72%) as a white solid.

^{1}H NMR (400 MHz, $CDCl_3$): δ (ppm) 2.55-2.49 (m, 2H, <u>CH_2</u>SH), 1.64-1.59 (m, 2H, <u>CH_2</u>CH_2SH), 1.37 (t, *J* = 6.3 Hz, 1H, SH), 1.35-1.26 (m, 28H, CH_2), 0.88 (t, 3H, *J* = 6.2 Hz, CH_3).

^{13}C NMR (100 MHz, $CDCl_3$): δ (ppm) 34.1, 31.9, 29.69(4C), 29.66(3C), 29.59, 29.52, 29.4, 29.1, 28.4, 24.6, 22.7, 14.1.

HRMS (APCI): m/z calculated for $C_{17}H_{37}S[M+H]^+$: 273.2610, found 273.2611.

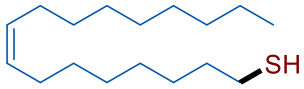

39

Corresponding NHPI ester (0.2 mmol) was thiolated according to the general procedure. The crude residue was purified by flash column chromatography (SiO_2; petroleum ether) to afford thiol **39** (33.5 mg, 62%) as a colorless oil.

^{1}H NMR (400 MHz, $CDCl_3$): δ (ppm) δ (ppm) 5.39-5.37 (m, 2H, 2×C=CH), 2.55-2.49 (m, 2H, <u>CH_2</u>SH), 1.99-1.94 (m, 4H, 2×C=CH<u>CH_2</u>)), 1.62-1.58 (m, 2H, <u>CH_2</u>CH_2SH), 1.35-1.26 (m, 21H, 10×CH_2 + SH), 0.88 (t, *J* = 6.7 Hz, 3H, CH_3).

^{13}C NMR (100 MHz, $CDCl_3$): δ (ppm) 130.5, 130.2, 34.0, 32.6, 32.5, 31.9, 29.6, 29.53, 29.48, 29.3, 29.2, 29.0, 28.9, 28.3, 24.6, 22.7, 14.1.

HRMS (APCI): m/z calculated for $C_{17}H_{35}S[M+H]^+$: 271.2454, found 271.2454.

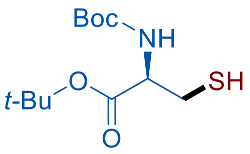

.

40

Corresponding NHPI ester (0.2 mmol) was thiolated according to the general procedure. The crude residue was purified by flash column chromatography (SiO_2; petroleum ether : EtOAc = 20:1) to afford thiol **40** (20.5 mg, 37%) as a colorless oil.

^{1}H NMR (400 MHz, $CDCl_3$): δ (ppm) 5.40 (br, 1H, NH), 4.47 (br, 1H, CH), 2.96 (d, *J* = 7.5 Hz, 2H, CH_2), 1.49 (s, 9H, $CHCOOC(CH_3)_3$), 1.45 (s, 9H, $NHCOOC(CH_3)_3$), 1.37 (t, *J* = 8.4 Hz, 1H, SH).

^{13}C NMR (100 MHz, $CDCl_3$): δ (ppm) 169.3, 155.1, 82.7, 80.0, 55.1, 28.3, 28.0, 27.5.

HRMS (APCI): m/z calculated for $C_{12}H_{24}NO_4S[M+H]^+$: 278.1421, found 278.1421.

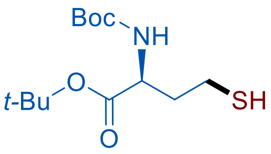

41

Corresponding NHPI ester (0.2 mmol) was thiolated according to the general procedure. The crude residue was purified by flash column chromatography (SiO_2; petroleum ether : EtOAc = 20:1) to afford thiol **41** (26.8 mg, 46%) as a colorless oil.

^{1}H NMR (400 MHz, $CDCl_3$): δ (ppm) 5.08 (br, 1H, NH), 4.31 (br, 1H, CHN), 2.65-2.54 (m, 2H, CH_2S), 2.09-2.08 (m, 1H, $SCH_2C\underline{H}^aH^b$), 1.94-1.85 (m, 1H, $SCH_2CH^a\underline{H}^b$), 1.59 (t, *J* = 6.8 Hz, 1H, SH), 1.48 (s, 9H, $CHCOOC(CH_3)_3$), 1.45 (s, 9H, $NHCOOC(CH_3)_3$).

^{13}C NMR (101 MHz, $CDCl_3$): δ (ppm) 171.4, 155.4, 82.2, 79.9, 52.9, 37.6, 28.3, 28.0, 20.8.

HRMS (EI): m/z calculated for $C_{13}H_{26}NO_4S$ $[M+H]^+$: 292.1577, found 292.1574.

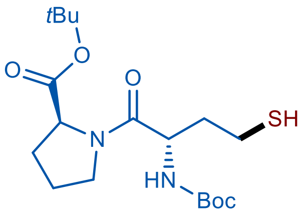

42

Corresponding NHPI ester (0.2 mmol) was thiolated according to the general procedure. The crude residue was purified by flash column chromatography (SiO_2; petroleum ether : EtOAc = 3:1) to afford thiol **42** (35.7 mg, 46%) as a colorless oil.

^{1}H NMR (600 MHz, $CDCl_3$): δ (ppm) 5.35 (d, *J* = 8.8 Hz, 1H, NH), 4.66 (td, *J* = 9.0, 3.8 Hz, 1H, OC<u>CH</u>NH), 4.40 (dd, *J* = 8.5, 4.6 Hz, 1H, NCH), 3.70 (m, 2H, NCH_2), 2.70 – 2.58 (m, 2H, C<u>H_2</u>SH), 2.21 – 2.15 (m, 1H, NCHC<u>H</u>^{b}H^a), 2.07 – 2.01 (m, 1H, NCHC<u>H</u>^{a}H^b), 2.00 – 1.93 (m, 3H, <u>CH_2</u>CH_2SH, CH_2C<u>H</u>^{b}H^aCH_2), 1.90 – 1.86 (m, 1H, CH_2C<u>H</u>^{a}H^bCH_2), 1.75 (t, *J* = 8.5 Hz, 1H, SH), 1.44 (s, 9H, $C(CH_3)_3$), 1.42 (s, 9H, $C(CH_3)_3$).

^{13}C NMR (151 MHz, $CDCl_3$): δ (ppm) 170.94, 170.44, 155.74, 81.43, 79.73, 59.62, 50.21, 47.01, 37.43, 29.02, 28.30, 27.95, 24.82, 20.74.

HRMS (ESI): m/z calculated for $C_{18}H_{33}N_2O_5S$ $[M+H]^+$: 389.2105, found 389.2094.

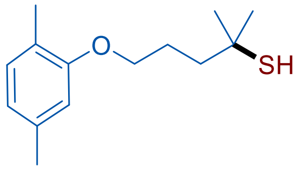

43

Corresponding NHPI ester (0.2 mmol) was thiolated according to the general procedure, using CF_3CH_2OH as solvent instead of CH_3CN. The crude residue was purified by flash column chromatography (SiO_2; petroleum ether) to afford thiol **43** (4.8 mg, 10%) as a colorless oil.

^{1}H NMR (400 MHz, $CDCl_3$): δ (ppm) 7.01 (d, *J* = 7.4 Hz, 1H, Ar-H), 6.66 (d, *J* = 7.6 Hz, 1H, Ar-H), 6.62 (s, 1H, Ar-H), 3.96 (t, *J* = 6.0 Hz, 2H, OCH_2), 2.31 (s, 3H, $ArCH_3$), 2.18 (s, 3H, $ArCH_3$), 1.99-1.92 (m, 2H, OCH_2<u>CH_2</u>), 1.78-1.74 (m, 2H, OCH_2CH_2<u>CH_2</u>), 1.70 (s, 1H, SH), 1.56 (s, 6H, 2×C-CH_3).

^{13}C NMR (100 MHz, $CDCl_3$): δ (ppm) 157.0, 136.5, 130.3, 123.6, 120.7, 112.0, 67.9, 44.5,

43.0, 32.8, 25.7, 21.4, 15.8.

HRMS (APCI): m/z calculated for $C_{14}H_{22}OSNa[M+Na]^+$: 261.1284, found 261.1284.

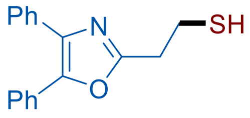

44

Corresponding NHPI ester (0.2 mmol) was thiolated according to the general procedure. The crude residue was purified by flash column chromatography (SiO_2; petroleum ether : EtOAc = 9:1) to afford thiol **44** (13.5 mg, 24%) as a colorless oil.

^{1}H NMR (400 MHz, $CDCl_3$): δ (ppm) 7.64 (d, J = 7.5 Hz, 2H, Ar-H), 7.58 (d, J = 7.5 Hz, 2H, Ar-H), 7.38-7.30 (m, 6H, ArH), 3.18 (t, J = 7.0 Hz, 2H, HS$\underline{CH_2}$), 3.05-3.00 (m, 2H, CH_2), 1.77 (t, J = 8.2 Hz, 1H, SH).

^{13}C NMR (100 MHz, $CDCl_3$): δ (ppm) 161.2, 145.5, 135.2, 132.4, 128.9, 128.6, 128.6, 128.5, 128.1, 127.9, 126.5, 32.8, 21.8.

HRMS (APCI): m/z calculated for $C_{17}H_{16}NOS$ $[M+H]^+$: 282.0947, found 282.0946.

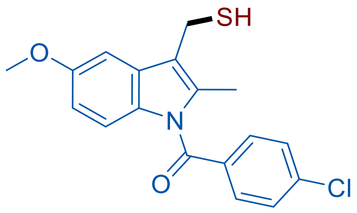

45

Corresponding NHPI ester (0.2 mmol) was thiolated according to the general procedure. The crude residue was purified by flash column chromatography (SiO_2; petroleum ether / EtOAc = 20:1) to afford thiol **45** (33.8 mg, 49%) as a pale yellow solid.

^{1}H NMR (400 MHz, $CDCl_3$): δ (ppm) 7.41-7.36 (m, 4H, Ar-H), 6.92 (d, J = 8.8 Hz, 1H, Ar-H), 6.68 (s, 1H, Ar-H), 6.64 (d, J = 9.0 Hz, 1H, Ar-H), 3.62 (s, 3H, OCH_3), 3.00 (s, 2H, $\underline{CH_2}$SH), 2.02 (s, 3H, CH_3).

^{13}C NMR (100 MHz, $CDCl_3$): δ (ppm) 168.2, 155.8, 139.0, 134.3, 134.0, 131.3, 130.9, 130.8, 129.0, 118.8, 115.0, 111.3, 100.6, 55.4, 23.7, 12.9.

HRMS (APCI): m/z calculated for $C_{18}H_{17}ClNO_2S[M+H]^+$: 346.0663, found 346.0663.

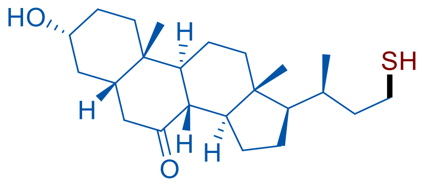

46

Corresponding NHPI ester (0.2 mmol) was thiolated according to the the general procedure. The crude residue was purified by flash column chromatography (SiO_2; petroleum ether / EtOAc = 20:1) to afford thiol **46** (24.2 mg, 32%) as a white solid.

^{1}H NMR (400 MHz, $CDCl_3$): δ (ppm) 3.63-3.57 (m, 1H, HOCH), 2.86 (dd, *J* = 12.4, 5.8 Hz, 1H, COCH), 2.66-2.57 (m, 1H), 2.46-2.36 (m, 2H), 2.23-2.16 (m, 1H), 2.01-1.09 (m, 24H), 1.00-0.90 (m, 4H), 0.66 (s, 3H).

^{13}C NMR (101 MHz, $CDCl_3$): δ (ppm) 212.0, 70.9, 54.8, 49.5, 48.9, 46.1, 45.4, 42.7, 42.7, 40.6, 38.9, 37.4, 35.1, 34.9, 34.1, 29.8, 28.4, 24.8, 23.0, 21.9, 21.7, 18.4, 12.0.

HRMS (ESI): m/z calculated for $C_{23}H_{39}O_2S$ $[M+H]^+$: 379.2665, found 379.2661.

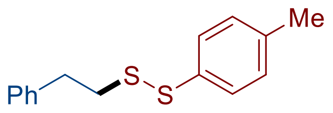

47

Corresponding NHPI ester (0.2 mmol) was thiolated according to the general procedure. Upon completion, the K_2CO_3 (55 mg, 0.4 mmol, 2.0 equiv.) and p-tolyl disulfide (49.3 mg, 0.2 mmol, 1.0 equiv.) was added under argon atmosphere. The tube stirred at ambient temperature for 6 hours in the dark. The crude residue was purified by flash column chromatography(SiO_2; petroleum ether) to afford sulfide **47** (33.8 mg, 65%) as a colorless oil.

^{1}H NMR (400 MHz, $CDCl_3$): δ (ppm) 7.43 (d, *J* = 7.7 Hz, 2H, ArH), 7.29-7.12 (m, 7H, ArH), 2.97 (s, 4H, CH_2CH_2), 2.33 (s, 3H, CH_3).

^{13}C NMR (100 MHz, $CDCl_3$): δ (ppm) 139.9, 137.2, 133.9, 129.8, 128.6, 128.6, 128.4, 126.4, 39.9, 35.3, 21.0.

HRMS (APCI): m/z calculated for $C_{15}H_{17}S_2[M+H]^+$: 261.0766, found 261.0767.

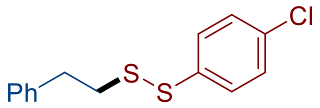

48

Corresponding NHPI ester (0.2 mmol) was thiolated according to the general procedure. Upon completion, the K_2CO_3 (55 mg, 0.4 mmol, 2.0 equiv.) and bis(4-cholorophenyl) disulfide (58 mg, 0.2 mmol, 1.0 equiv.) was added under argon atmosphere. The tube stirred at ambient temperature for 6 hours in the dark. The crude residue was purified by flash column chromatography(SiO_2; petroleum ether) to afford sulfide **48** (32.5 mg, 58%) as a colorless oil.

^{1}H NMR (400 MHz, $CDCl_3$): δ (ppm) 7.45 (d, J = 7.6 Hz, 2H, ArH), 7.28-7.19 (m, 5H, ArH), 7.14 (d, J = 7.1 Hz, 2H, ArH), 2.96 (s, 4H, CH_2CH_2).

^{13}C NMR (100 MHz, $CDCl_3$): δ (ppm) 139.6, 136.0, 132.8, 129.1, 128.9, 128.5, 128.5, 126.5, 40.0, 35.2.

HRMS (APCI): m/z calculated for $C_{14}H_{13}ClS_2[M]^{\cdot+}$: 280.0142, found 280.0143.

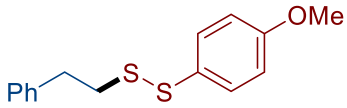

49

Corresponding NHPI ester (0.2 mmol) was thiolated according to the general procedure. Upon completion, the K_2CO_3 (55 mg, 0.4 mmol, 2.0 equiv.) and bis(4-methoxyphenyl) disulfide (56 mg, 0.2 mmol, 1.0 equiv.) was added under argon atmosphere. The tube stirred at ambient temperature for 6 hours in the dark. The crude residue was purified by flash column chromatography(SiO_2; petroleum ether) to afford sulfide **49** (30.9 mg, 56%) as a colorless oil.

^{1}H NMR (400 MHz, CDCl$_3$): δ (ppm) 7.49 (d, *J* = 7.6 Hz, 2H, ArH), 7.30-7.14 (m, 5H, ArH), 6.86 (d, *J* = 7.6 Hz, 2H, ArH), 3.80 (s, 3H, OCH$_3$), 2.97 (s, 4H, CH$_2$CH$_2$).

^{13}C NMR (100 MHz, CDCl$_3$): δ (ppm) 159.6, 140.0, 131.9, 128.6, 128.5, 128.2, 126.4, 114.7, 55.4, 39.9, 35.2.

HRMS (APCI): m/z calculated for $C_{15}H_{16}OS_2[M]^{\cdot+}$: 276.0637, found 276.0637.

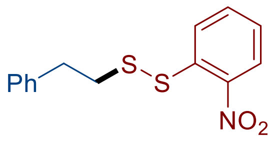

50

Corresponding NHPI ester (0.2 mmol) was thiolated according to the general procedure. Upon completion, the K_2CO_3 (55 mg, 0.4 mmol, 2.0 equiv.) and bis(2-nitrophenyl) disulfide (62 mg, 0.2 mmol, 1.0 equiv.) was added under argon atmosphere. The tube stirred at ambient temperature for 6 hours in the dark. The crude residue was purified by flash column chromatography(SiO$_2$; petroleum ether) to afford sulfide **50** (31.4 mg, 54%) as a colorless oil.

^{1}H NMR (400 MHz, CDCl$_3$): δ (ppm) 8.26 (d, *J* = 7.8 Hz, 1H, Ar-H), 8.19 (d, *J* = 8.0 Hz, 2H, Ar-H), 7.62 (t, *J* = 7.2 Hz, 1H, Ar-H), 7.36-7.16 (m, 6H, Ar-H), 2.99 (m, 4H, ArCH$_2$CH$_2$).

^{13}C NMR (101 MHz, CDCl$_3$): δ (ppm) 145.67, 139.28, 137.62, 133.95, 128.57, 128.55, 127.26, 126.62, 126.12, 126.06, 39.46, 35.31.

HRMS (EI): m/z calculated for $C_{14}H_{14}NO_2S_2$ $[M+H]^+$: 292.0460, found 292.0459.

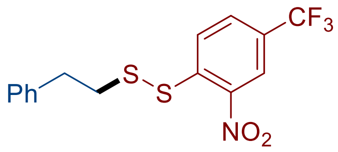

51

Corresponding NHPI ester (0.2 mmol) was thiolated according to the general procedure. Upon completion, the K_2CO_3 (55 mg, 0.4 mmol, 2.0 equiv.) and 4,4'-Bis(trifluoromethyl)-2,2'-dinitrodiphenyl disulfide (89 mg, 0.2 mmol, 1.0 equiv.) was

added under argon atmosphere. The tube stirred at ambient temperature for 6 hours in the dark. The crude residue was purified by flash column chromatography(SiO_2; petroleum ether / EtOAc = 20:1) to afford sulfide **51** (35.9 mg, 50%) as a yellow oil.

^{1}H NMR (400 MHz, $CDCl_3$): δ (ppm) 8.51 (s, 1H, Ar-H)，8.32 (d, *J* = 8.4 Hz, 1H, Ar-H), 7.80 (d, *J* = 8.4 Hz, 1H, Ar-H), 7.31-7.21 (m, 3H, Ar-H), 7.16 (d, *J* = 6.8 Hz, 2H, Ar-H), 3.01 (s, 4H, $ArCH_2CH_2$).

^{13}C NMR (101 MHz, $CDCl_3$): δ (ppm) 145.3, 142.6, 138.9, 129.9 (q, J_{C-F} = 3.4 Hz), 128.7 (q, J_{C-F} = 34.7 Hz), 128.6, 128.5, 128.2, 126.8, 123.4 (q, J_{C-F} = 3.7 Hz), 122.8 (q, J_{C-F} = 273.7 Hz), 39.6, 35.2.

HRMS (EI): m/z calc. for $C_{15}H_{13}F_3NO_2S_2$ $[M+H]^+$: 360.0334, found 360.0334.

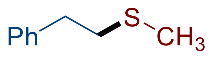

52

Corresponding NHPI ester (0.2 mmol) was thiolated according to the general procedure. Upon completion, the DIPEA (66 μl, 0.4 mmol, 2.0 equiv.) and chloroiodomethane (141 mg, 0.8 mmol, 4.0 equiv.) was added under argon atmosphere. The tube stirred at ambient temperature for 6 hours in the dark. The crude residue was purified by flash column chromatography(SiO_2; petroleum ether) to afford sulfide **52** (12.5 mg, 41%) as a colorless oil.

^{1}H NMR (400 MHz, $CDCl_3$): δ (ppm) 7.32-7.20 (m, 5H, Ar-H), 2.90 (t, *J* = 7.7 Hz, 2H, $ArCH_2$), 2.75 (t, *J* = 7.7 Hz, 2H, CH_2S), 2.12 (s, 3H, CH_3).

^{13}C NMR (101 MHz, $CDCl_3$): δ (ppm) 140.5, 128.4, 128.4, 126.3, 35.9, 35.8, 15.7.

HRMS (APCI): m/z calculated for $C_9H_{13}S$ $[M+H]^+$: 153.0732, found 153.0732.

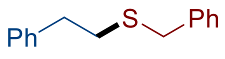

53

Corresponding NHPI ester (0.2 mmol) was thiolated according to the general procedure. Upon completion, the DIPEA (66 μl, 0.4 mmol, 2.0 equiv.) and Benzyl bromide (68 mg, 0.4 mmol, 2.0 equiv.) was added under argon atmosphere. The tube stirred at ambient temperature

for 6 hours in the dark. The crude residue was purified by flash column chromatography(SiO_2; petroleum ether) to afford sulfide **53** (18.2 mg, 40%) as a colorless oil.

^{1}H NMR (400 MHz, $CDCl_3$): δ (ppm) 7.29-7.12 (m, 10H, Ar-H), 3.69 (s, 2H, $PhCH_2S$), 2.82 (t, *J* = 7.4 Hz, 2H, Ph$\underline{CH_2}$$CH_2$), 2.82 (t, *J* = 7.4 Hz, 2H, Ph$CH_2$$\underline{CH_2}$).

^{13}C NMR (100 MHz, $CDCl_3$): δ (ppm) 140.5, 138.4, 128.8, 128.43, 128.38(2C), 126.9, 126.2, 36.4, 36.0, 32.7.

HRMS (APCI): m/z calculated for $C_{15}H_{17}S[M+H]^+$: 229.1045, found 229.1045.

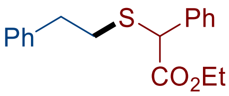

54

Corresponding NHPI ester (0.2 mmol) was thiolated according to the general procedure. Upon completion, the DIPEA (66 μl, 0.4 mmol, 2.0 equiv.) and Ethyl α-bromophenylacetate (97mg, 0.4 mmol, 2.0 equiv.) was added under argon atmosphere. The tube stirred at ambient temperature for 6 hours in the dark. The crude residue was purified by flash column chromatography(SiO_2; petroleum ether) to afford sulfide **54** (37.8 mg, 63%) as a colorless oil.

^{1}H NMR (400 MHz, $CDCl_3$): δ (ppm) 7.45 (d, *J* = 7.5 Hz, 2H, Ar-H), 7.36-7.18 (m, 6H, Ar-H), 7.14 (t, *J* = 7.4 Hz, 2H, Ar-H), 4.56 (s, 1H, Ar-CH), 4.23-4.12 (m, 2H, OCH_2), 2.86-2.72 (m, 4H, $ArCH_2CH_2S$), 1.25 (t, *J* = 7.1 Hz, 3H, CH_3).

^{13}C NMR (101 MHz, $CDCl_3$): δ (ppm) 170.7, 140.1, 136.1, 128.6, 128.47(2C), 128.43, 128.1, 126.4, 61.6, 52.3, 35.7, 33.2, 14.0.

HRMS (APCI): m/z calculated for $C_{18}H_{21}O_2S$ $[M+H]^+$: 301.1257, found 301.1257.

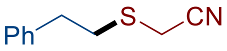

55

Corresponding NHPI ester (0.2 mmol) was thiolated according to the general procedure. Upon completion, the DIPEA (66 μl, 0.4 mmol, 2.0 equiv.) and Chloroacetonitrile (30 mg, 0.4

mmol, 2.0 equiv.) was added under argon atmosphere. The tube stirred at ambient temperature for 6 hours in the dark. The crude residue was purified by flash column chromatography(SiO_2; petroleum ether) to afford sulfide **55** (24.1 mg, 68%) as a colorless oil.

^{1}H NMR (400 MHz, $CDCl_3$): δ (ppm) 7.26-7.22 (m, 5H, Ar-H), 3.19 (s, 2H, CH_2CN), 3.02-2.94 (m, 4H, $ArCH_2CH_2S$).

^{13}C NMR (100 MHz, $CDCl_3$): δ (ppm) 139.2, 128.6, 128.5, 126.7, 116.4, 35.3, 33.8, 17.1.

HRMS (APCI): m/z calculated for $C_{10}H_{12}NS[M+H]^+$: 178.0685, found 178.0685.

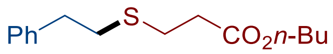

56

Corresponding NHPI ester (0.2 mmol) was thiolated according to the general procedure. Upon completion, the DIPEA (66 μl, 0.4 mmol, 2.0 equiv.) and Butyl acrylate (58 μl, 0.4 mmol, 2.0 equiv.) was added under argon atmosphere. The tube stirred at ambient temperature for 6 hours in the dark. The crude residue was purified by flash column chromatography(SiO_2; petroleum ether : EtOAc = 70:1) to afford sulfide **56** (42.6 mg, 80%) as a colorless oil.

^{1}H NMR (400 MHz, $CDCl_3$): δ (ppm) 7.31-7.27 (m, 2H, Ar-H), 7.22-7.19 (m, 3H, Ar-H), 4.09 (t, J = 6.4 Hz, 2H, OCH_2), 2.89 (t, J = 7.6 Hz, 2H, $ArCH_2$), 2.80-2.77 (m, 4H, $SCH_2CH_2C{=}O$), 2.59 (t, J = 7.2 Hz, 2H, $S\underline{CH_2}$), 1.64-1.57 (m, 2H, $OCH_2\underline{CH_2}$), 1.42-1.33 (m, 2H, $\underline{CH_2}CH_3$), 0.93 (t, J = 7.1 Hz, 3H, CH_3).

^{13}C NMR (100 MHz, $CDCl_3$): δ (ppm) 171.9, 140.3, 128.4(2C), 126.3, 64.5, 36.2, 34.8, 33.6, 30.6, 27.1, 19.1, 13.6.

HRMS (APCI): m/z calculated for $C_{15}H_{23}O_2S[M+H]^+$: 267.1413, found 267.1413.

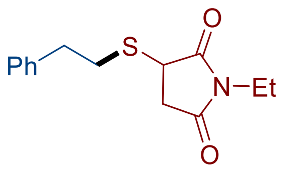

57

Corresponding NHPI ester (0.2 mmol) was thiolated according to the general procedure. Upon completion, the DIPEA (66 μl, 0.4 mmol, 2.0 equiv.) and N-Ethylmaleimide (50.1 mg, 0.4 mmol, 2.0 equiv.) was added under argon atmosphere. The tube stirred at ambient temperature for 6 hours in the dark. The crude residue was purified by flash column chromatography(SiO_2; petroleum ether : EtOAc = 70:1) to afford sulfide **57** (36.8 mg, 70%) as a colorless oil.

^{1}H NMR (400 MHz, $CDCl_3$): δ (ppm) 7.33-7.22 (m, 5H, Ar-H), 3.65 (dd, *J* = 8.9, 2.2 Hz, 1H, SCH), 3.57 (q, *J* = 7.1 Hz, 2H, NCH_2), 3.23-3.15 (m, 1H, COC$\underline{H^a}$H^b), 3.09-2.90(m, 4H, $ArCH_2CH_2$), 2.46 (dd, *J* = 18.6, 2.2 Hz, 1H, COCH$^a\underline{H^b}$), 1.17 (t, *J* = 7.1 Hz, 3H, CH_3).

^{13}C NMR (101 MHz, $CDCl_3$): δ (ppm) 176.28, 174.40, 139.65, 134.03, 128.48, 126.53, 38.92, 35.91, 35.59, 33.94, 33.06, 12.79.

HRMS (EI): m/z calculated for $C_{14}H_{18}NO_2S$ $[M+H]^+$: 264.1052, found 264.1050 .

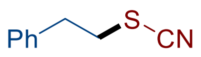

58

Corresponding NHPI ester (0.2 mmol) was thiolated according to the general procedure. Upon completion, the DIPEA (66 μl, 0.4 mmol, 2.0 equiv.) and Tosyl cyanide (72 mg, 0.4 mmol, 2.0 equiv.) was added under argon atmosphere. The tube stirred at ambient temperature for 6 hours in the dark. The crude residue was purified by flash column chromatography(SiO_2; petroleum ether) to afford sulfide **58** (21.2 mg, 65%) as a colorless oil.

^{1}H NMR: (400 MHz, CDCl3) δ (ppm) 7.34 (t, *J* = 7.2 Hz, 2H, ArH), 7.28-7.25 (m, 1H, ArH), 7.22 (d, *J* = 7.4 Hz, 2H, ArH), 3.17 (t, *J* = 7.2 Hz, 2H, CH_2SCN), 3.11 (t, *J* = 7.0 Hz, 2H, $PhCH_2$).

^{13}C NMR: (100 MHz, CDCl3) δ (ppm) 137.6, 128.8, 128.6, 127.2, 112.0, 36.0, 35.1.

HRMS (EI): m/z calculated for $C_9H_{10}NS$ $[M+H]^+$: 164.0528, found 164.0524 .

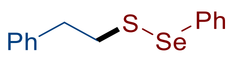

59

Corresponding NHPI ester (0.2 mmol) was thiolated according to the general procedure. Upon completion, the DIPEA (66 μl, 0.4 mmol, 2.0 equiv.) and Diphenyl diselenide (62 mg, 0.2 mmol, 1.0 equiv.) was added under argon atmosphere. The tube stirred at ambient temperature for 6 hours in the dark. The crude residue was purified by flash column chromatography(SiO_2; petroleum ether) to afford sulfide **59** (29.4 mg, 50%) as a yellow oil.

^{1}H NMR (400 MHz, $CDCl_3$): δ (ppm) 7.63 (d, *J* = 6.9 Hz, 2H, Ar-H), 7.32-7.18 (m, 6H, Ar-H), 7.15 (d, *J* = 7.0 Hz, 2H, Ar-H), 3.09 (t, *J* = 7.4 Hz, 2H, SCH_2), 2.95 (t, *J* = 7.6 Hz, 2H, $ArCH_2$).

^{13}C NMR (101 MHz, $CDCl_3$): δ (ppm) 139.9, 132.1, 130.1, 129.2, 128.6, 128.5, 127.4, 126.4, 39.3, 36.8.

HRMS (EI): m/z calculated for $C_{14}H_{15}SSe$ $[M+H]^+$: 289.0114, found 289.0114 .

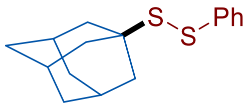

60

Corresponding NHPI ester (0.2 mmol) was sulfurized according to the general procedure. The crude residue was purified by flash column chromatography (SiO_2; petroleum ether) to afford disulfide **60** (27.6 mg, 50%) as a colorless oil.

^{1}H NMR (400 MHz, $CDCl_3$): δ (ppm) 7.55 (d, *J* = 6.7 Hz, 2H, Ar-H), 7.28 (t, *J* = 8.4 Hz, 2H, Ar-H), 7.15 (t, *J* = 8.6 Hz, 1H, Ar-H), 2.02 (s, 3H), 1.84 (s, 6H), 1.64 (s, 6H).

^{13}C NMR (100 MHz, $CDCl_3$): δ (ppm) 139.2, 128.6, 126.5, 126.0, 50.7, 42.5, 36.04, 29.9.

HRMS (APCI): m/z calculated for $C_{16}H_{21}S_2$$[M+H]^+$: 214.9536, found 214.9536.

Supplementary Tables

Supplementary Table 1. Screening of sulfur donor.[a]

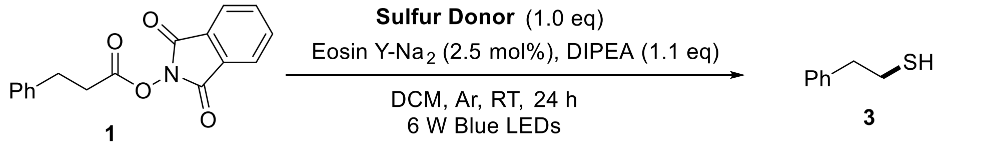

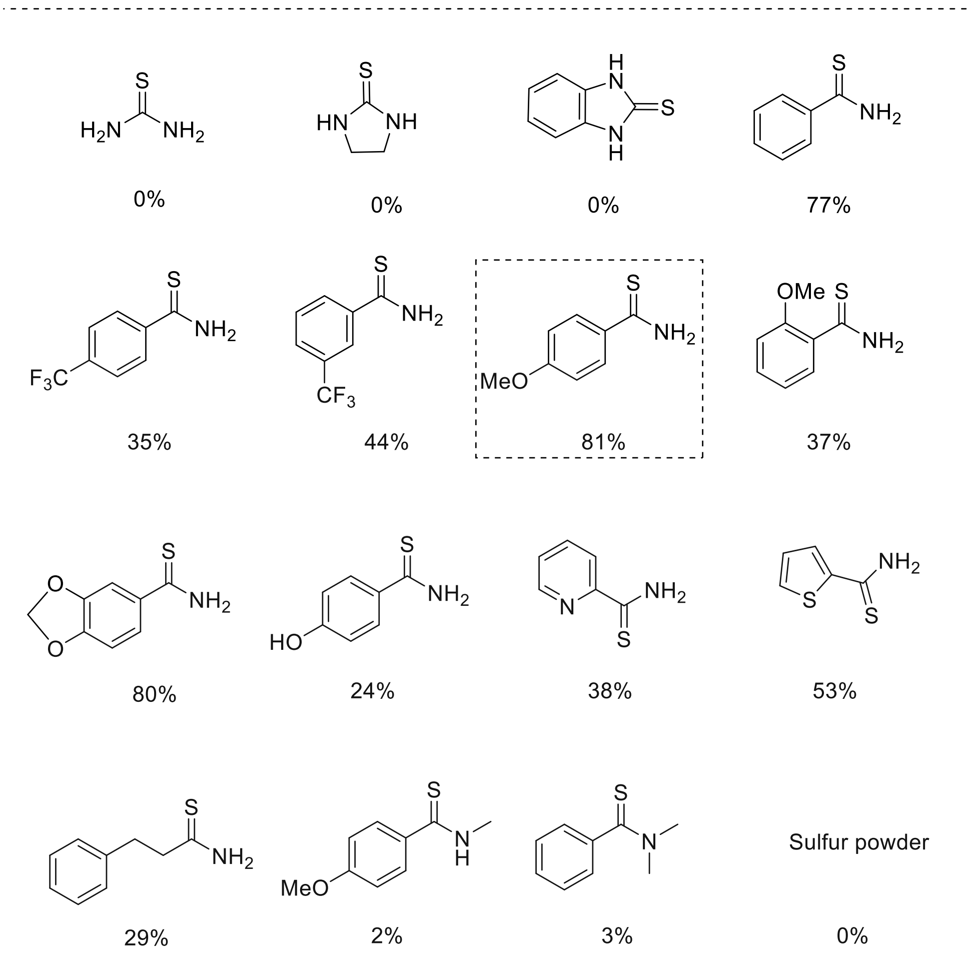

[a]The yield was determined by GC using anisole as an internal standard.

Supplementary Table 2. Screening of photocatalyst.[a]

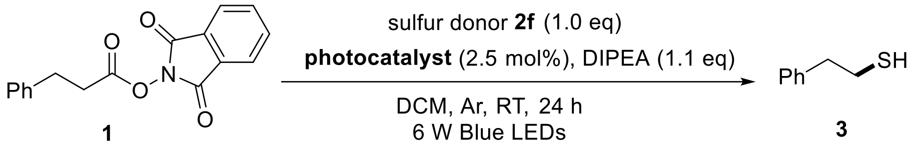

Entry	Photocatalyst	Yield[a]
1	Rhodamine B	76%
2	Fluorescein	71%
3	Eosin Y-Na_2	81%
4	Eosin Y-H_2	81%
5	$[Ir(ppy)_2(dtbbpy)]PF_6$	37%
6	$Ir(ppy)_3$	37%
7	$Ru(bpy)_3Cl_2$•$6H_2O$	71%

[a]The yield was determined by GC using anisole as an internal standard.

Supplementary Table 3. Screening of Solvent and Light Source.[a]

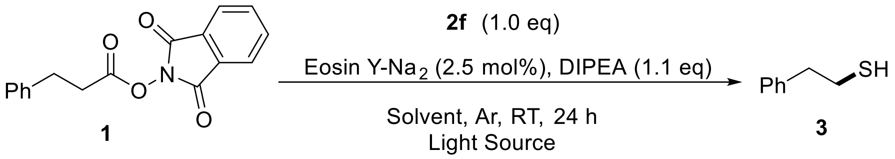

Entry	Solvent	Light Source	Yield[a]
1	DCM	blue LEDs	81%
2	CH_3CN	blue LEDs	83%
3	THF	blue LEDs	36%
4	Toluene	blue LEDs	22%
5	CH_3CN	purple LEDs	53%
6	CH_3CN	green LEDs	69%
7[b]	CH_3CN	blue LEDs	38%
8[c]	CH_3CN	blue LEDs	22%
9	CH_3CN	In dark	0%
10[d]	CH_3CN	blue LEDs	68%
11[e]	CH_3CN	blue LEDs	59%
12[f]	CH_3CN	blue LEDs	88%

[a]The yield was determined by GC using anisole as an internal standard.

[b]Without catalyst.

[c]Without DIPEA.

[d]With K_2CO_3 instead of DIPEA.

[e]"one-pot" conditions with carboxylic acid by pre-formation of **1**.

[f]With 2 equiv of **2f**.

TEMPO trapping experiment

To an oven-dried 10-ml Schlenk tube equipped with a magnetic stir bar and a Teflon coated septum screwcap was added the NHPI redox-active ester (0.2 mmol, 1.0 equiv.), 4-methoxythiobenzamide (**2f**, 0.4 mmol, 2.0 equiv.), eosin Y-Na_2 (2.5 mol%), TEMPO (0.60 mmol, 3 equiv.). The tube was evacuated and back-filled with argon for three cycles. The DIPEA (0.22 mmol, 1.1 equiv.) and dry CH_3CN (2.0 ml) was added via a gastight syringe under argon atmosphere. Make sure the screwcap was closed, the solvent was frozen by liquid nitrogen. Then the screwcap was opened and the tube was evacuated for about 3 mins. The screwcap was closed and let the solvent melts in a tepid water bath. Repeat above Freeze-Pump-Thaw procedures for 3-5 times until you no longer see the evolution of gas as the solution thaws. The tube was filled with argon and sealed, irradiated with 6W blue LED reactor and stirred at ambient temperature for 24 hours. A sample of the reaction mixture was then submitted to HRMS analysis, which indicated the phenylethyl radical formed after decarboxylation and was trapped by TEMPO.

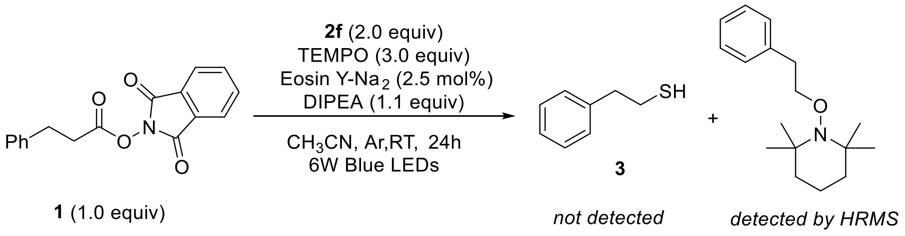

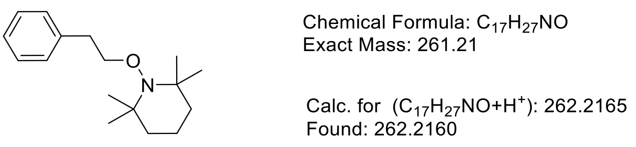

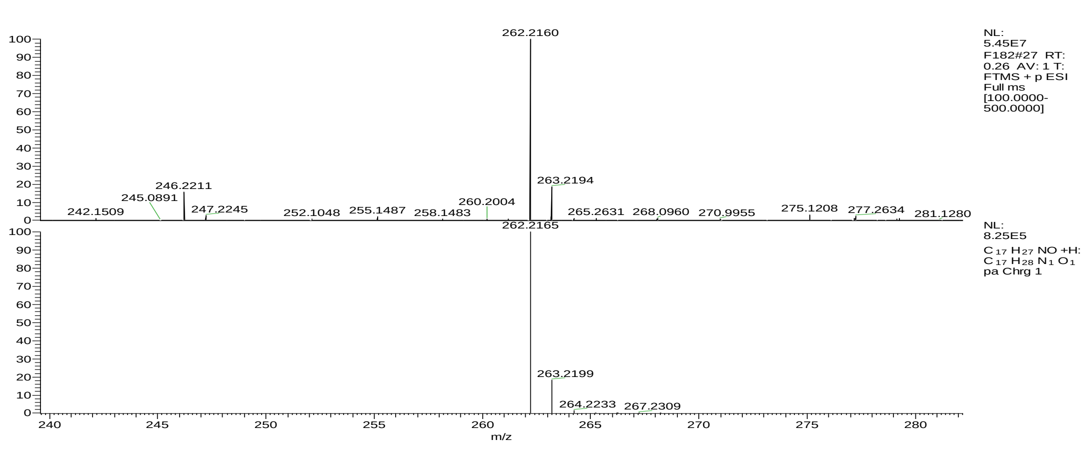

Supplementary Figure 3. HRMS spectra. Trapping reaction with TEMPO.

Competing experiment

To an oven-dried 10-ml Schlenk tube equipped with a magnetic stir bar and a Teflon coated septum screwcap was added the NHPI redox-active ester (0.2 mmol, 1.0 equiv.), 4-methoxythiobenzamide (**2f**, 0.4 mmol, 2.0 equiv.), *n*-butyl acrylate (0.4 mmol, 2.0 equiv.), eosin Y-Na_2 (2.5 mol%). The tube was evacuated and back-filled with argon for three cycles. The DIPEA (0.22 mmol, 1.1 equiv.) and dry CH_3CN (2.0 ml) was added via a gastight syringe under argon atmosphere. Make sure the screwcap was closed, the solvent was frozen by liquid nitrogen. Then the screwcap was opened and the tube was evacuated for about 3 mins. The screwcap was closed and let the solvent melts in a tepid water bath. Repeat above Freeze-Pump-Thaw procedures for 3-5 times until you no longer see the evolution of gas as the solution thaws. The tube was filled with argon and sealed, irradiated with 6W blue LED reactor and stirred at ambient temperature for 24 hours.

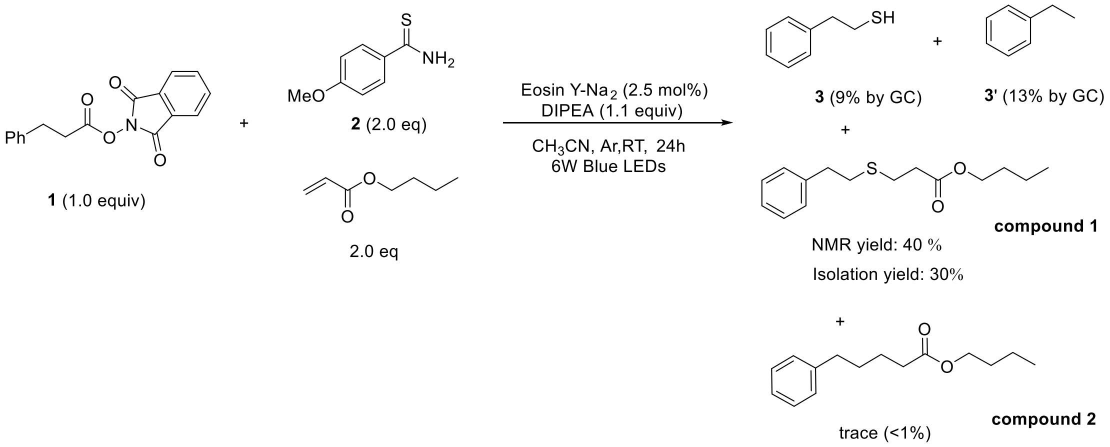

A sample of the reaction mixture was then submitted to HRMS and NMR analysis. We found **3** and compound 1 were obtained as the major products, and only a trace amount of compound 2 was detected by GC-MS.

UV-Visible Absorption

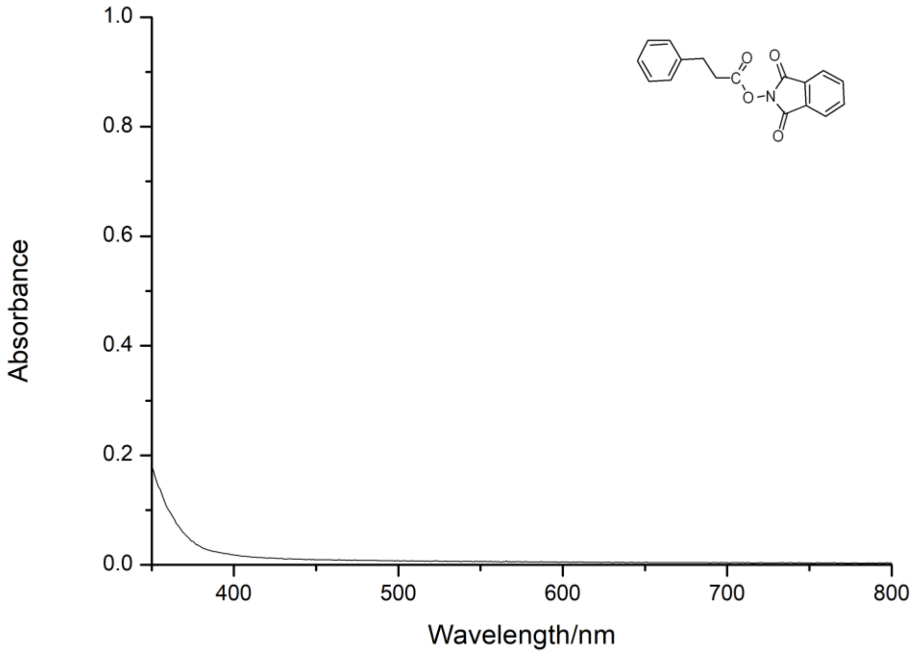

Supplementary Figure 4. UV-Vis Absorption of **1** (in CH_3CN).

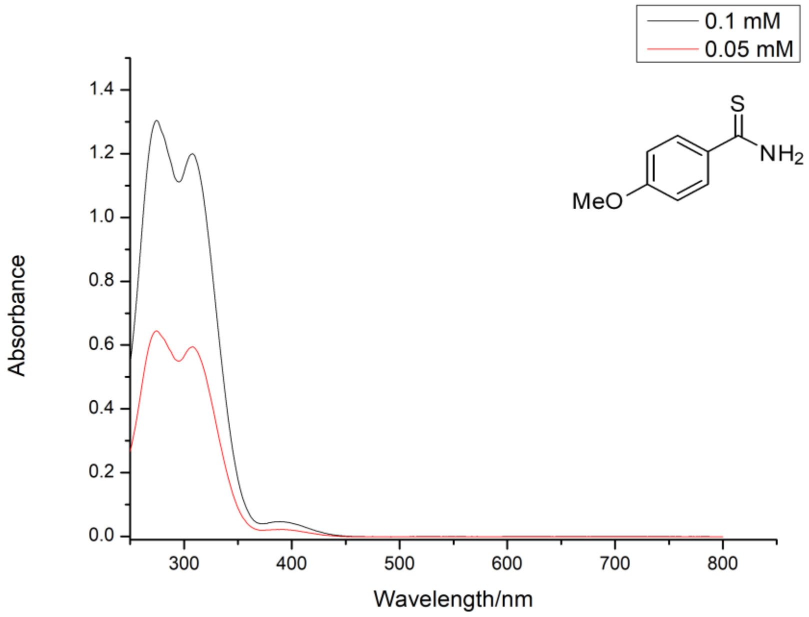

Supplementary Figure 5. UV-Vis Absorption of **2f** (in CH_3CN).

Fluorescence Titration of Photocatalyst

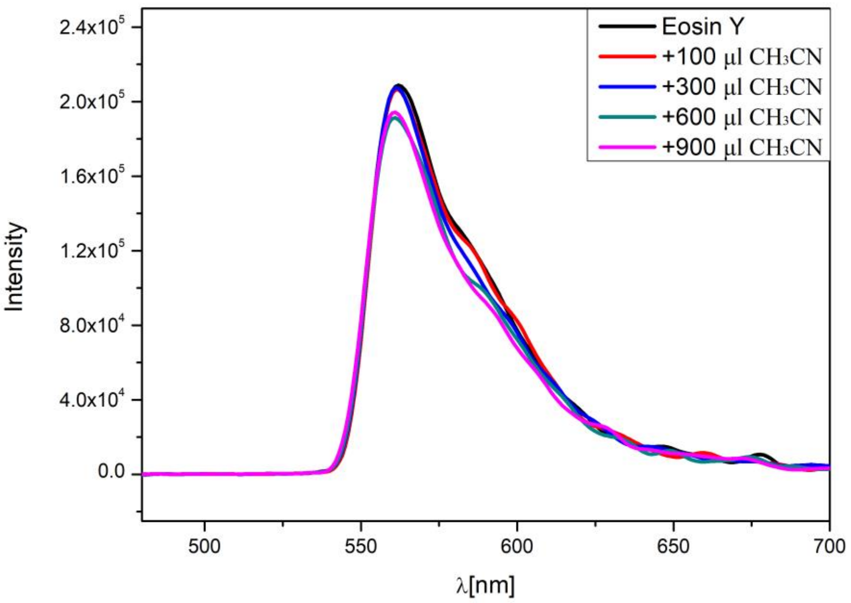

Supplementary Figure 6. Fluorescence titration of Eosin Y (187.5 µM in CH_3CN) with blank sample

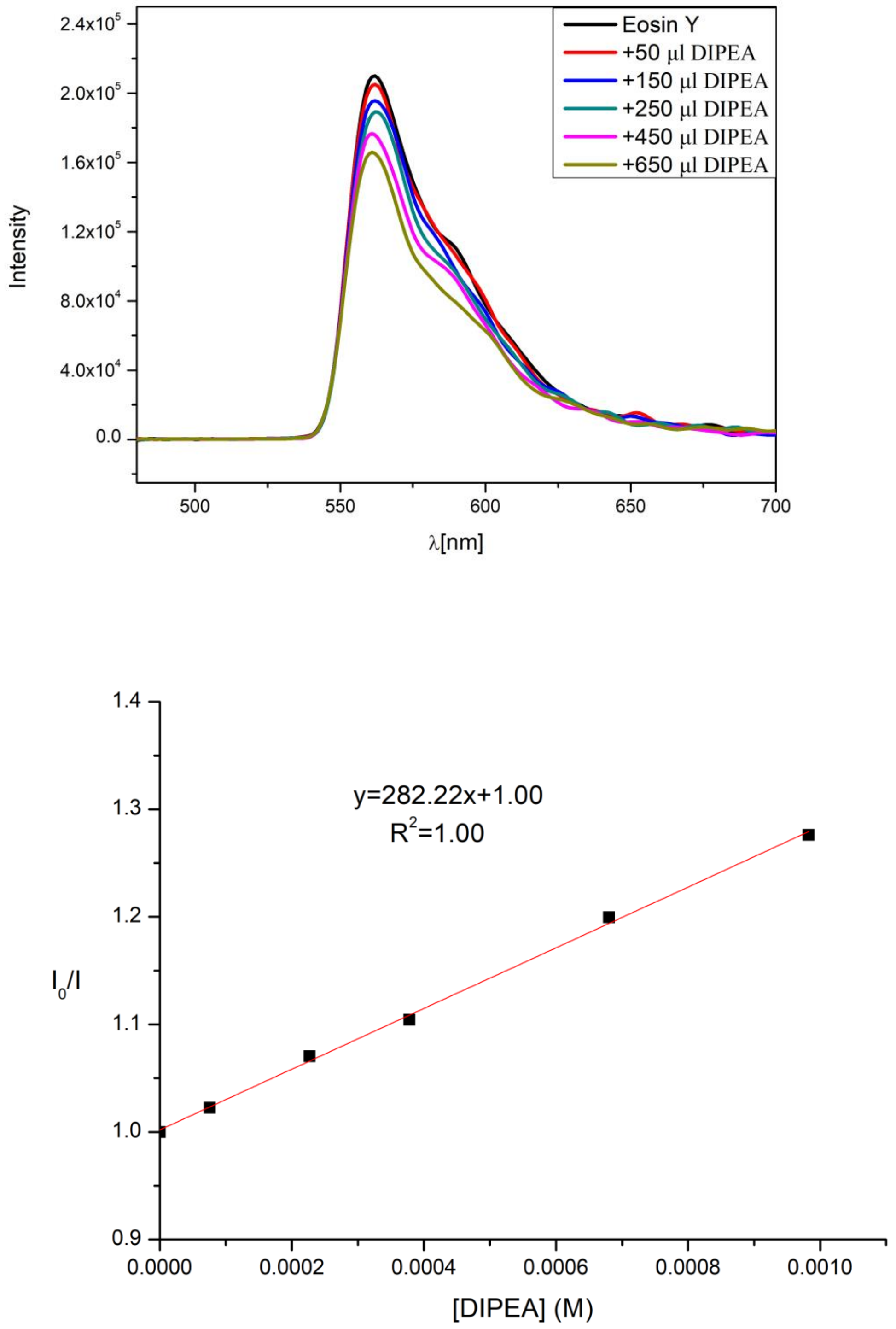

Supplementary Figure 7. Fluorescence titration of Eosin Y (187.5 μM in CH_3CN) with DIPEA (100 mM in CH_3CN).

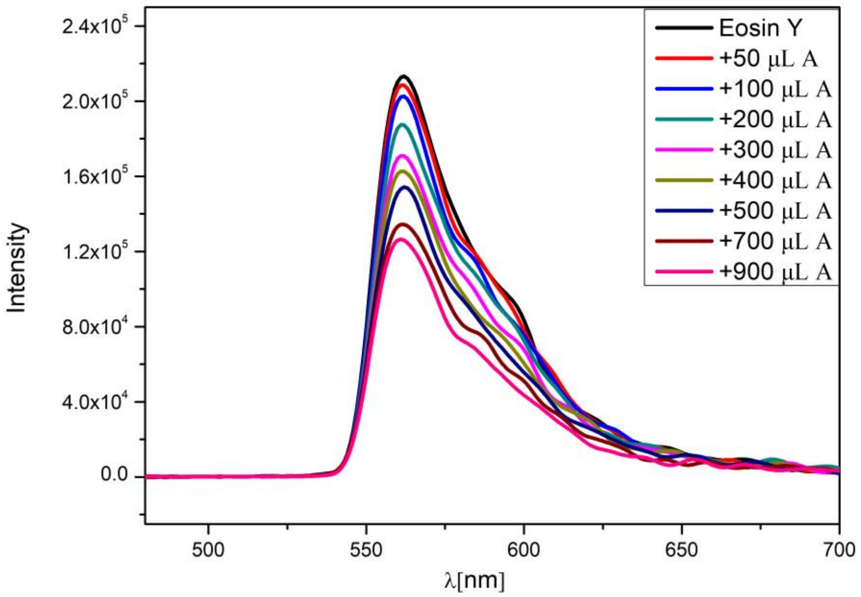

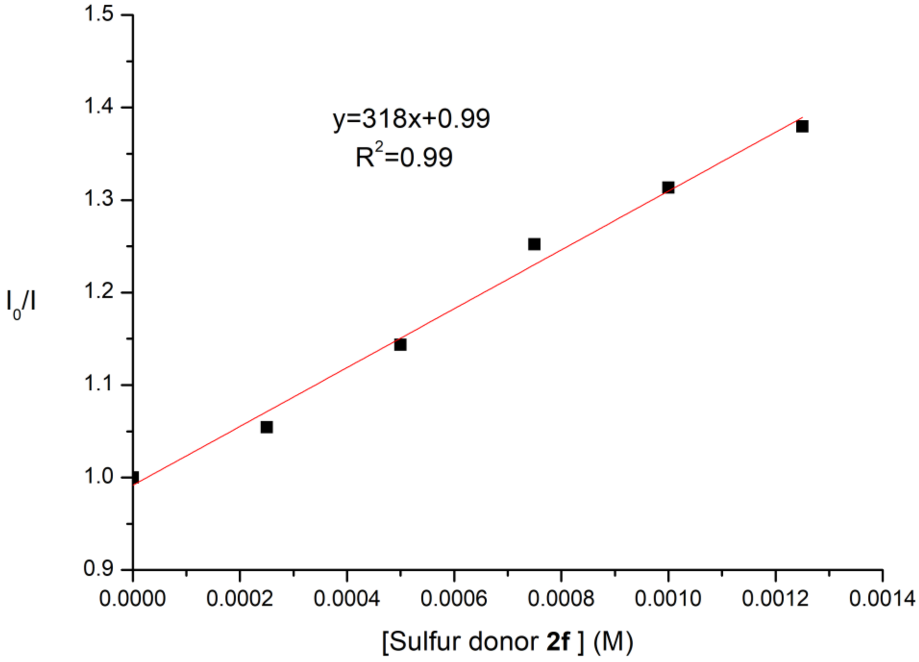

Supplementary Figure 8. Fluorescence titration of Eosin Y (187.5 μM in CH_3CN) with **2f** (**A**, 100 mM in CH_3CN).

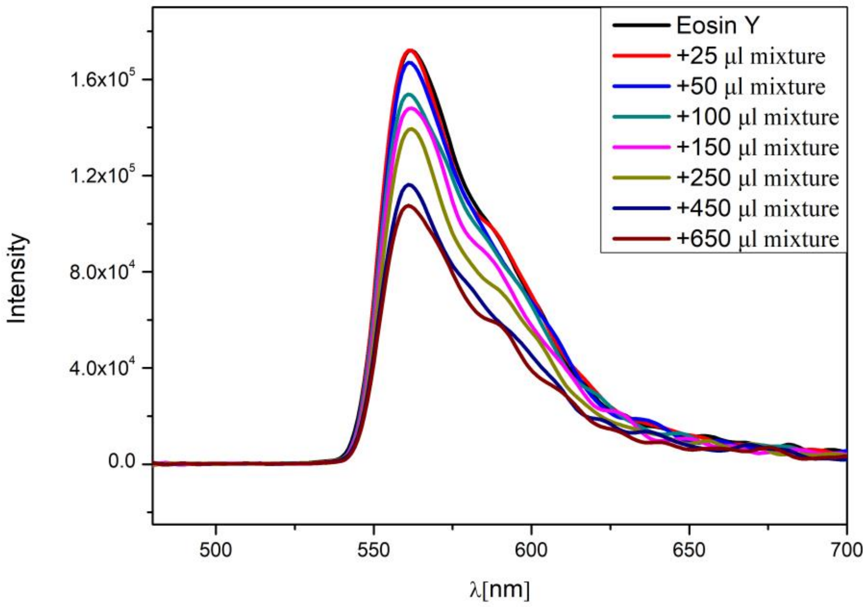

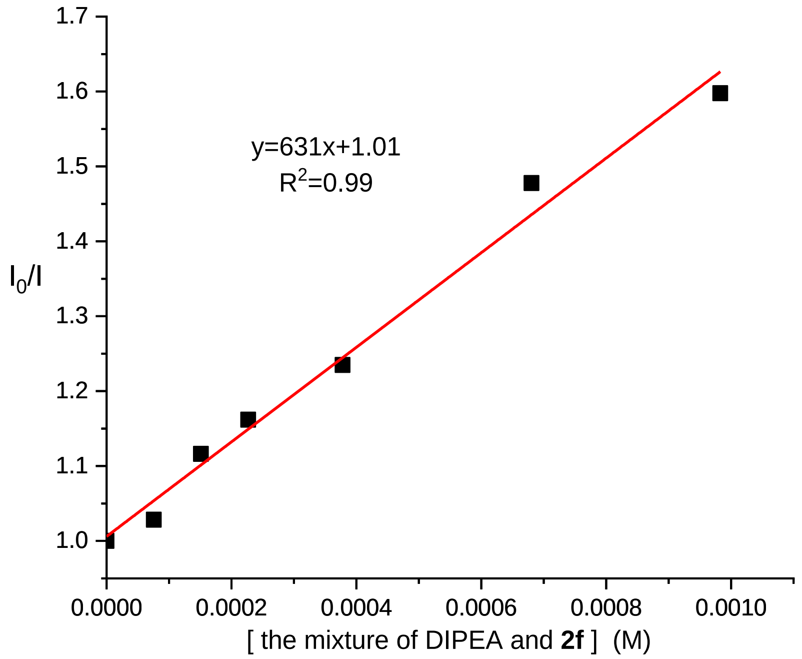

Supplementary Figure 9. Fluorescence titration of Eosin Y (187.5 μM in CH_3CN) with the mixture of DIPEA and **2f** (**mixture**, 100 mM in CH_3CN).

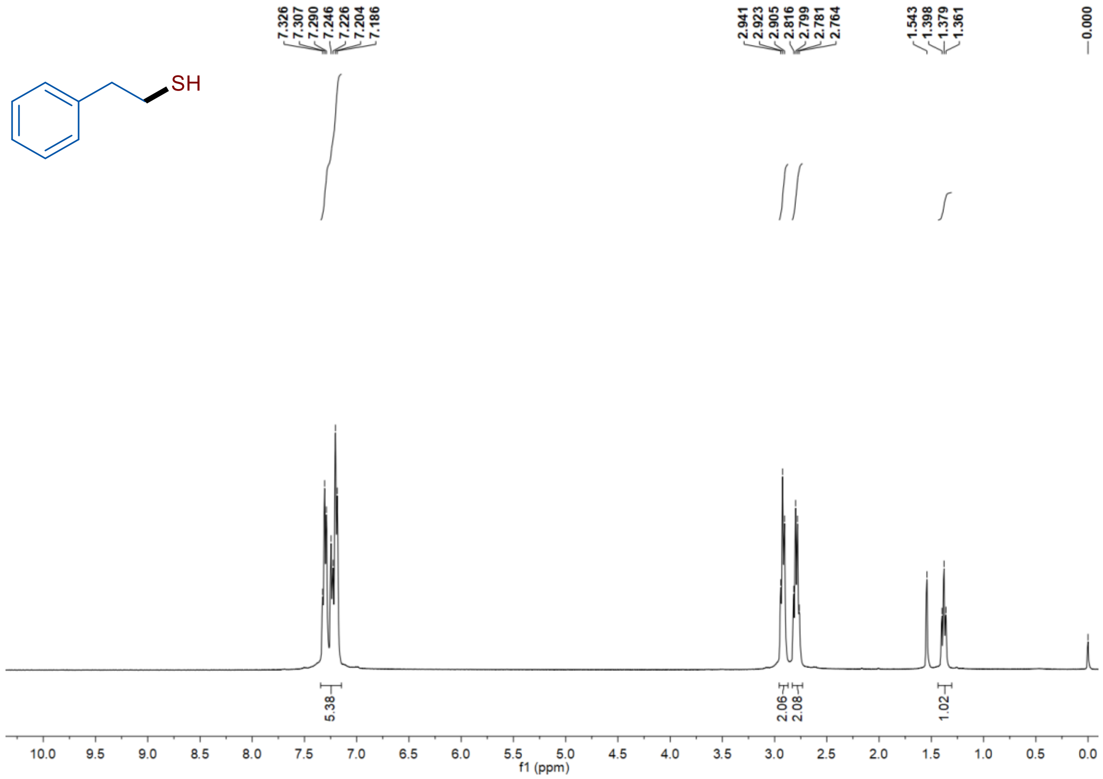

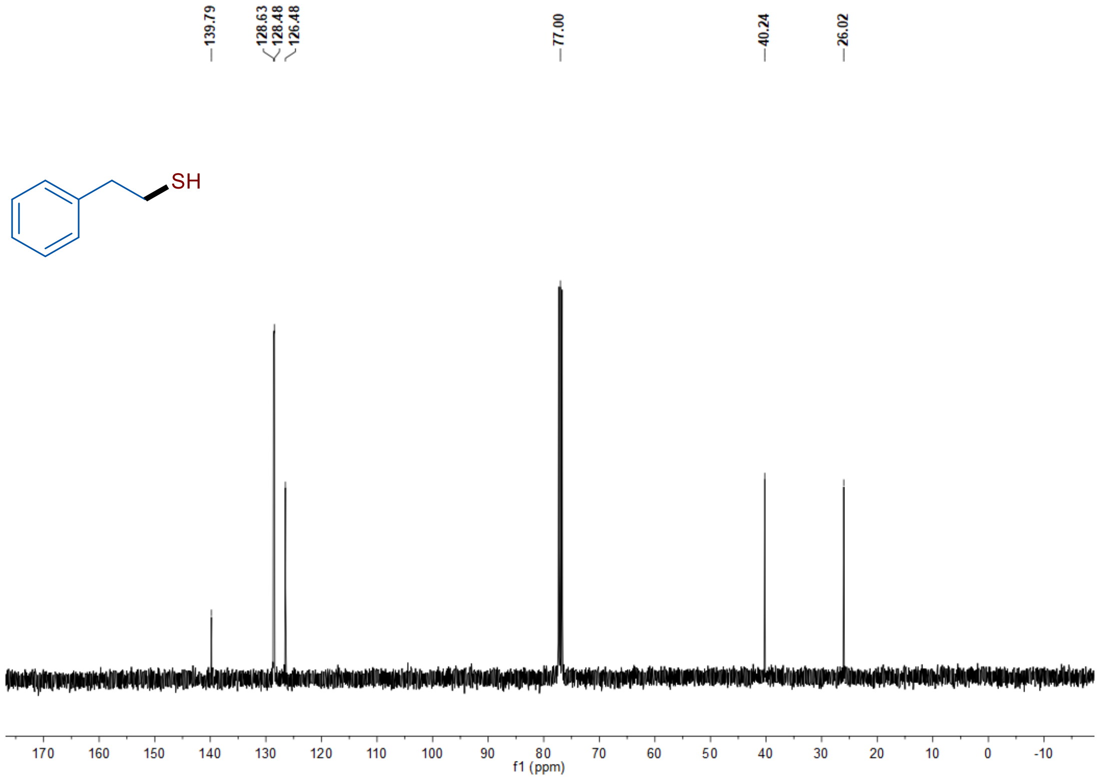

Supplementary Figure 10. ^{1}H and ^{13}C NMR spectra for compound **3**

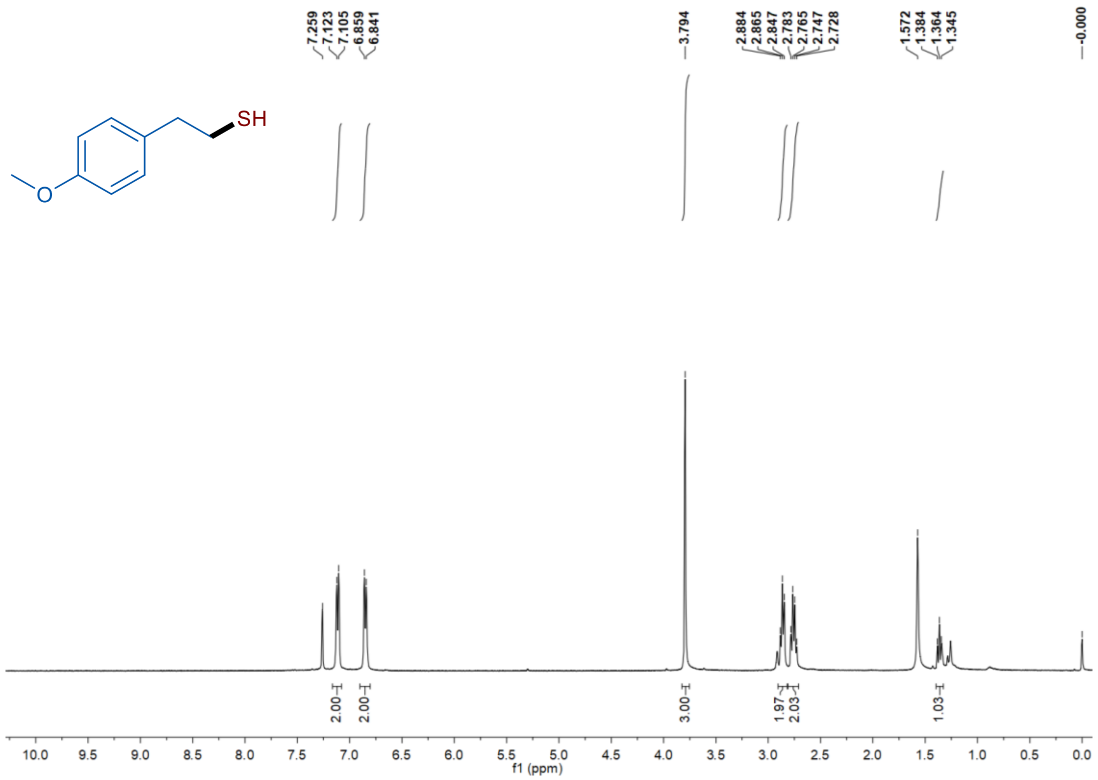

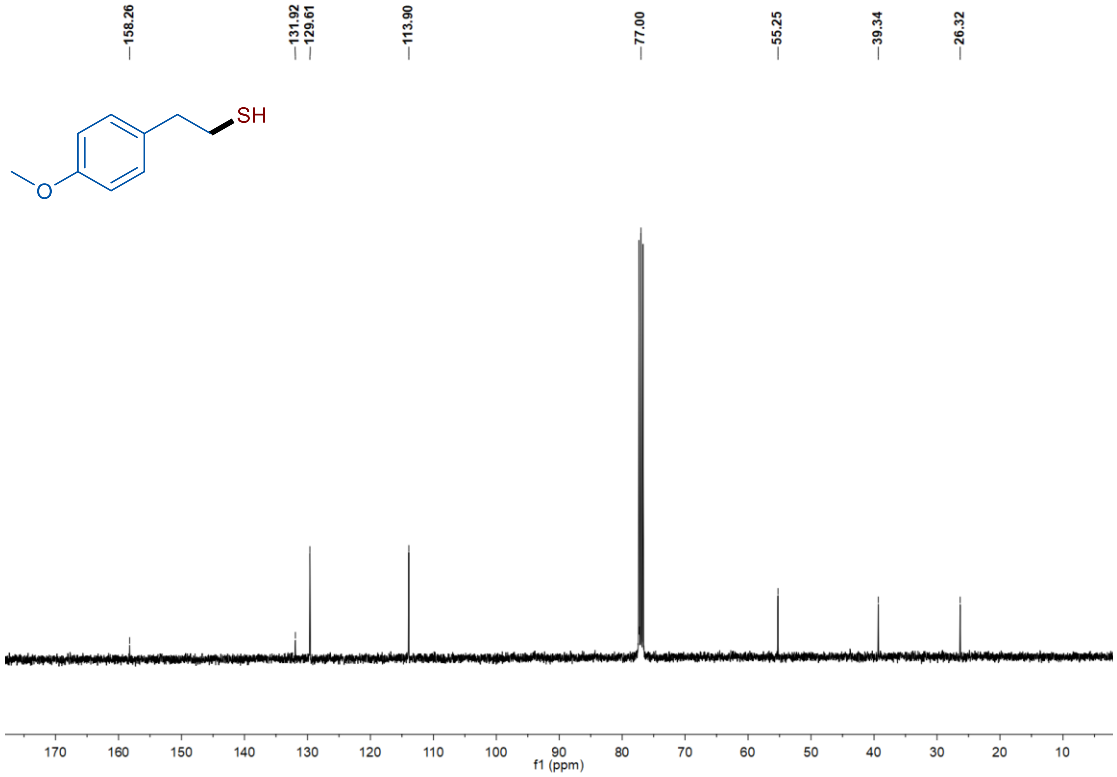

Supplementary Figure 11. ^{1}H and ^{13}C NMR spectra for compound **4**

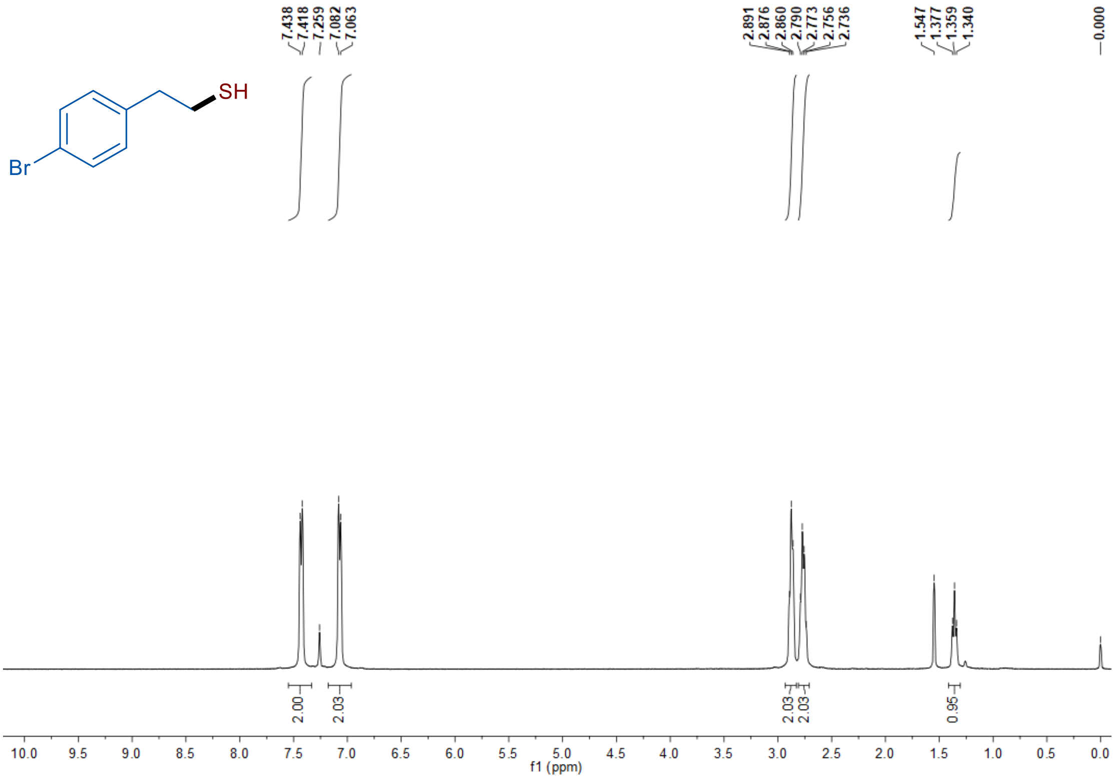

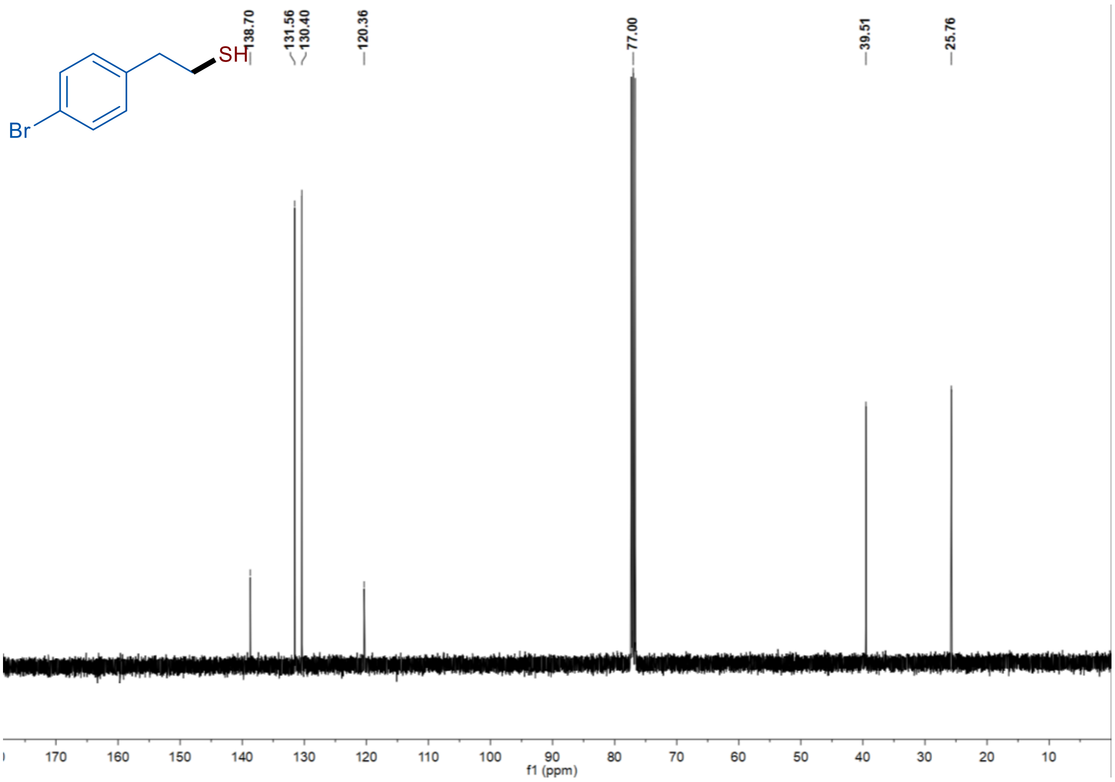

Supplementary Figure 12. ^{1}H and ^{13}C NMR spectra for compound **5**

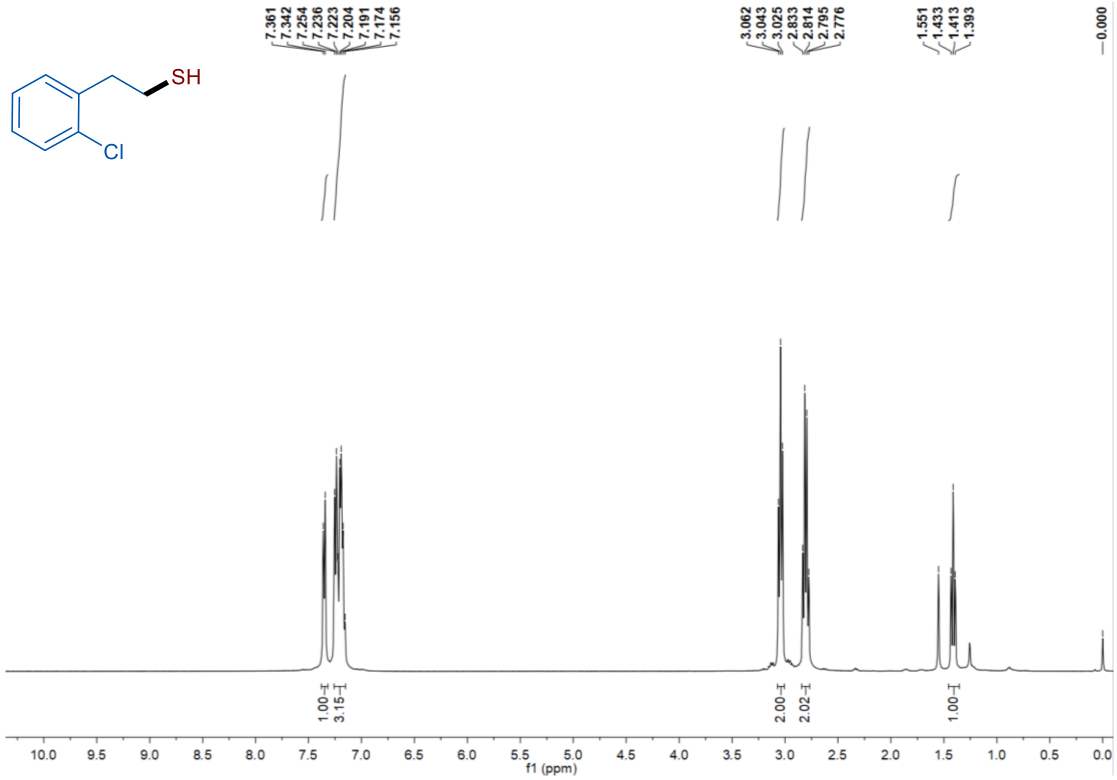

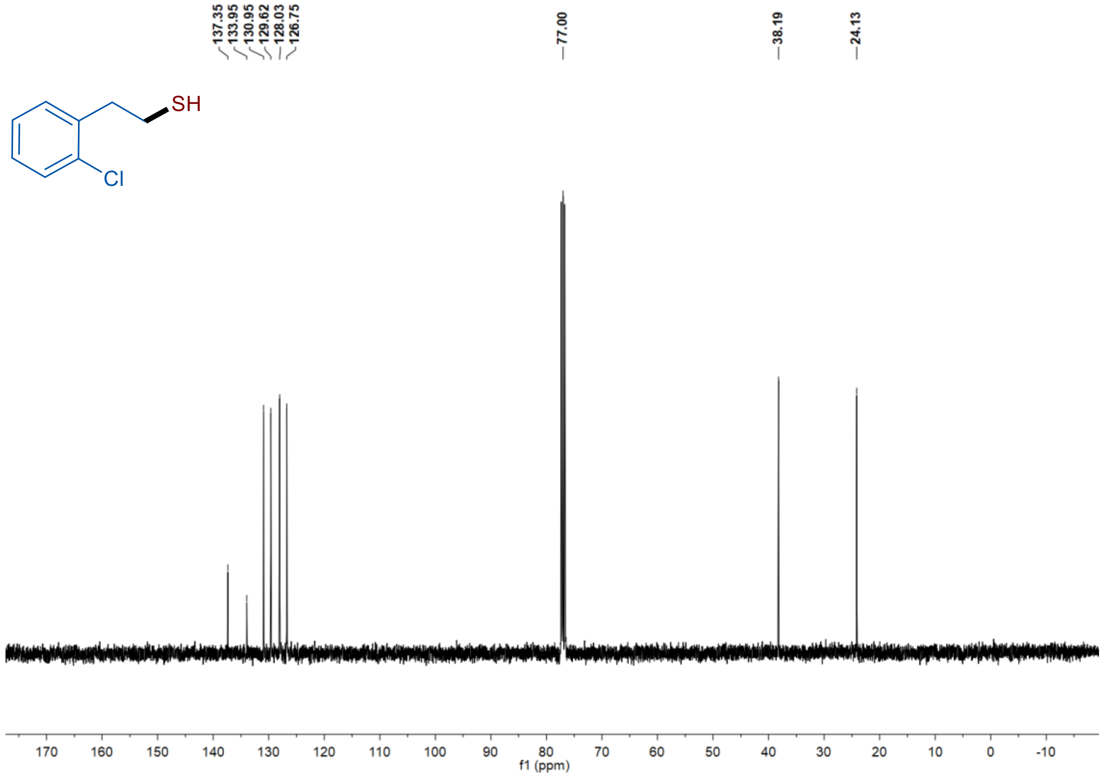

Supplementary Figure 13. ^{1}H and ^{13}C NMR spectra for compound **6**

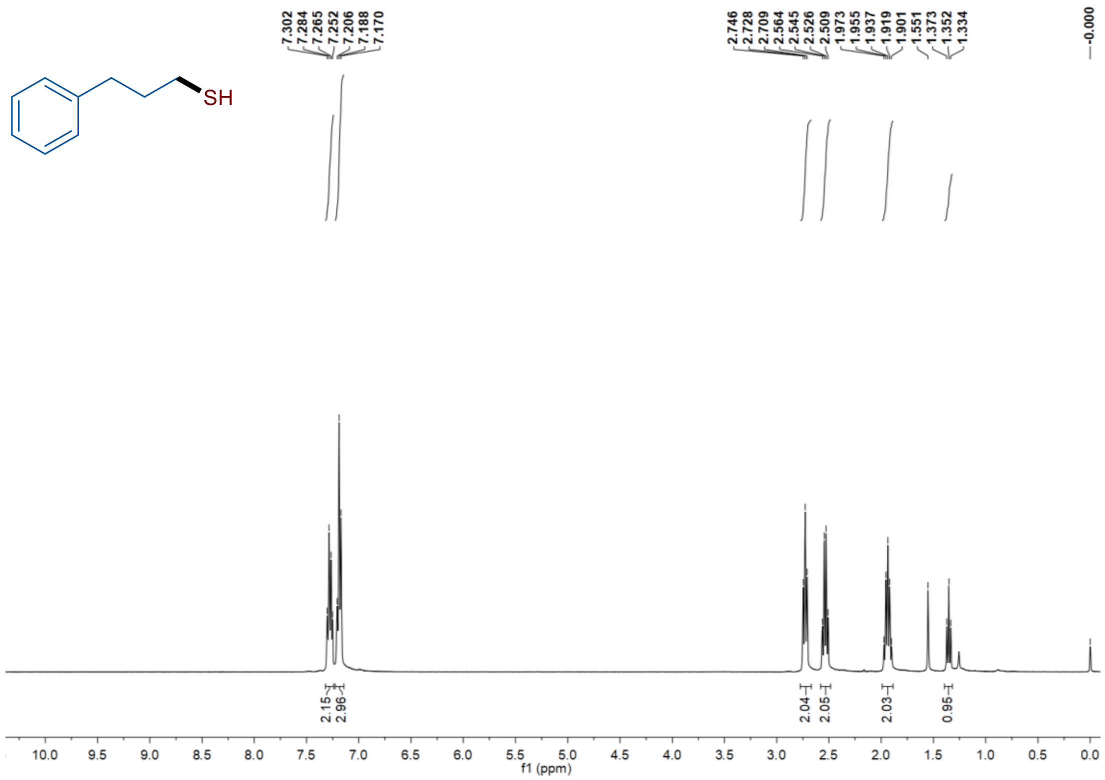

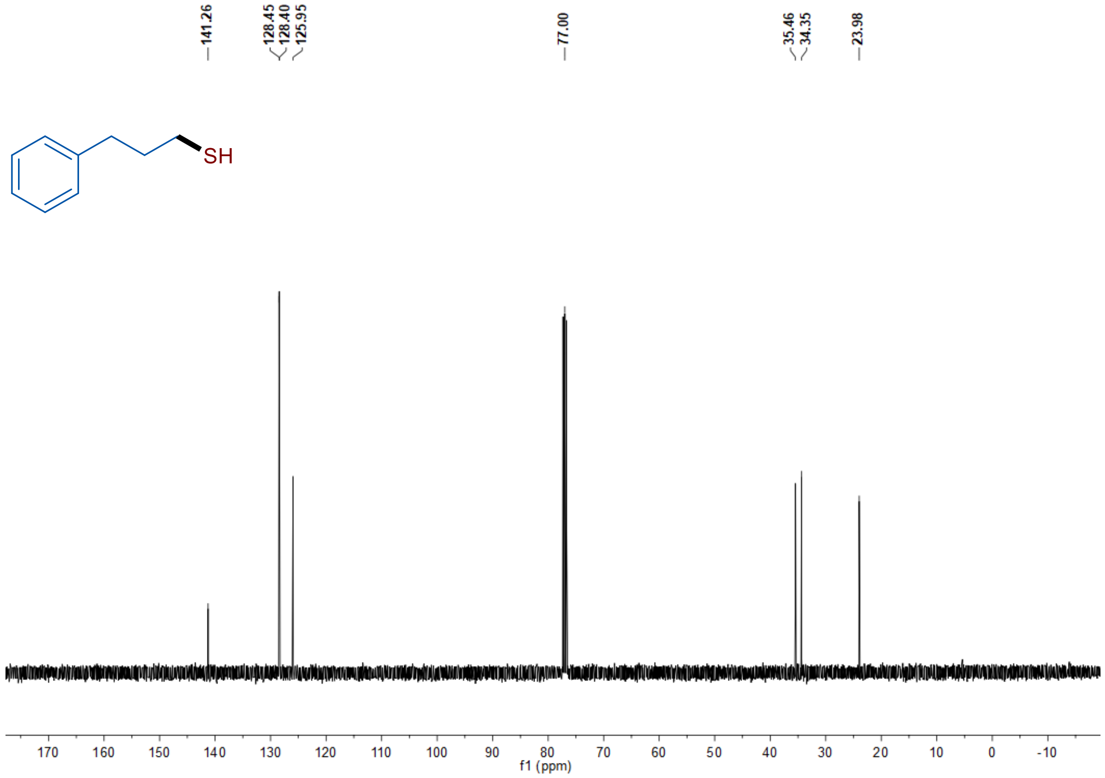

Supplementary Figure 14. ^{1}H and ^{13}C NMR spectra for compound **7**

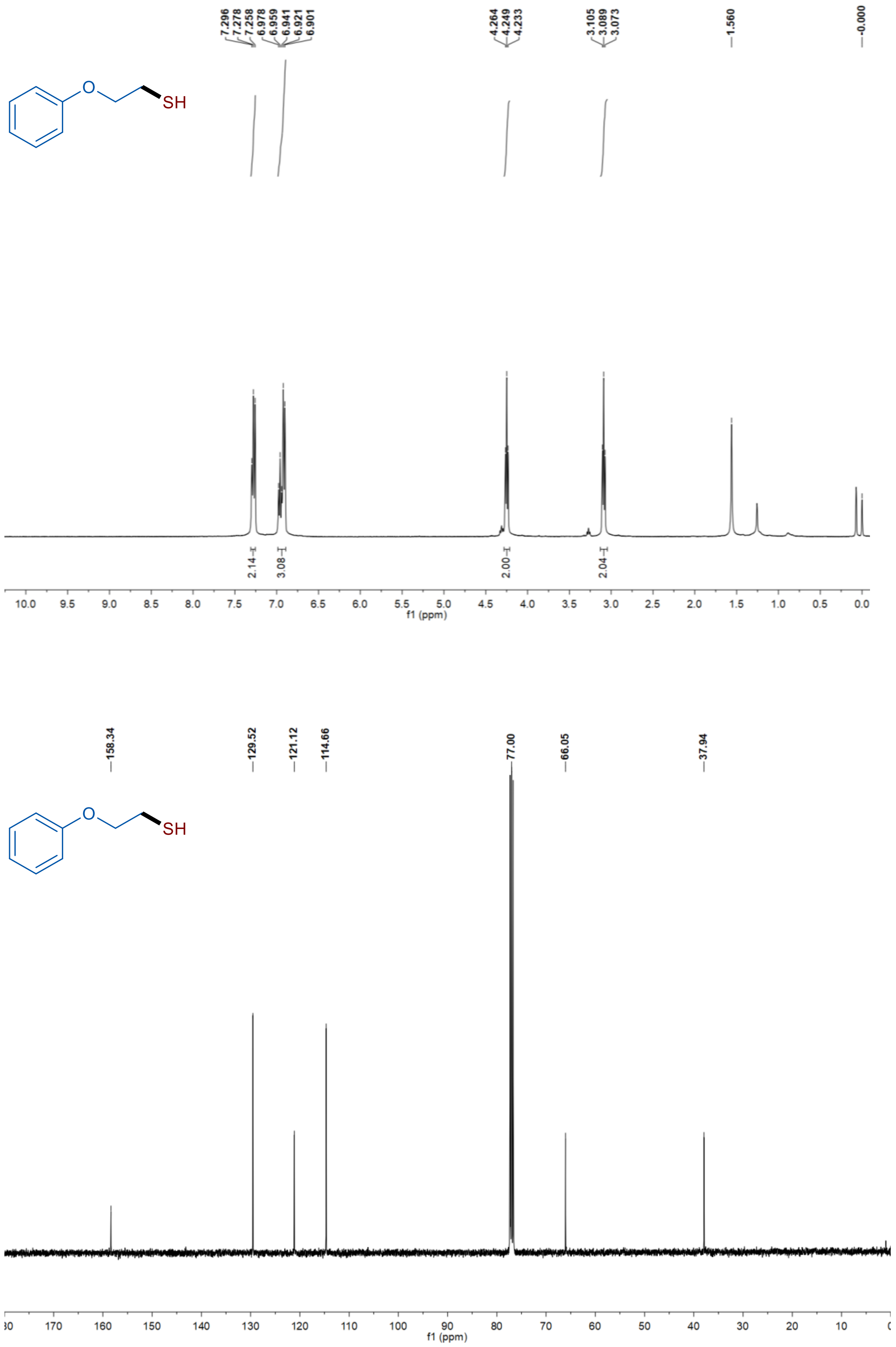

Supplementary Figure 15. ^{1}H and ^{13}C NMR spectra for compound **8**

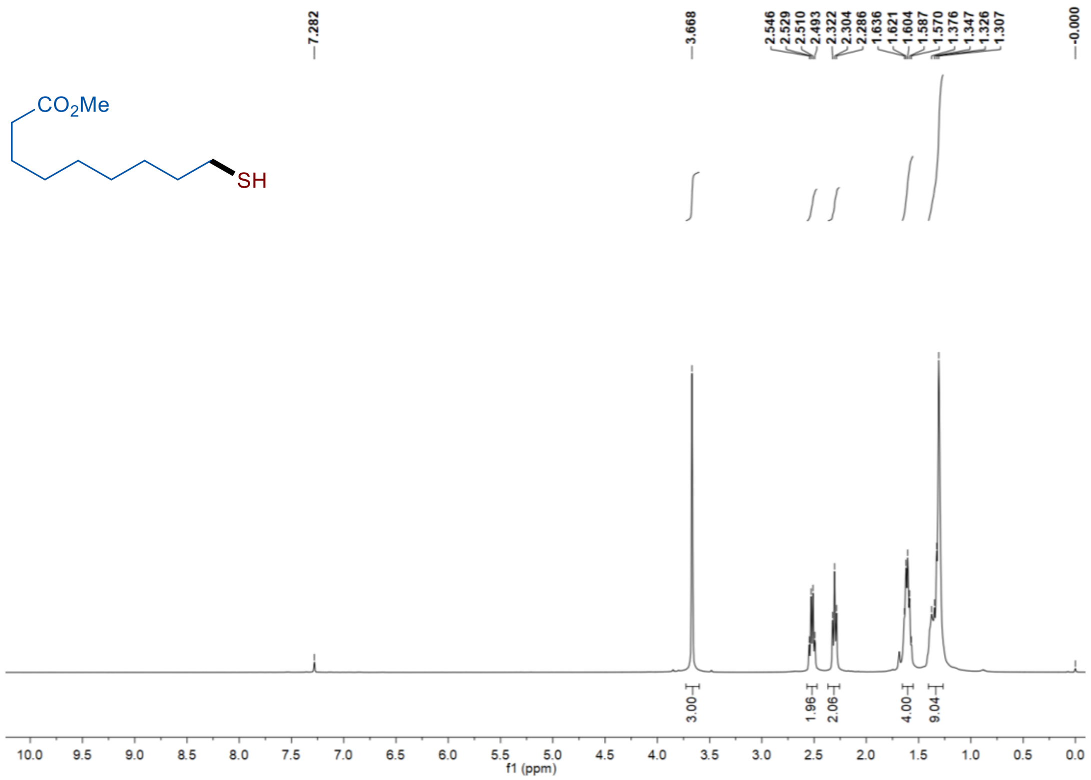

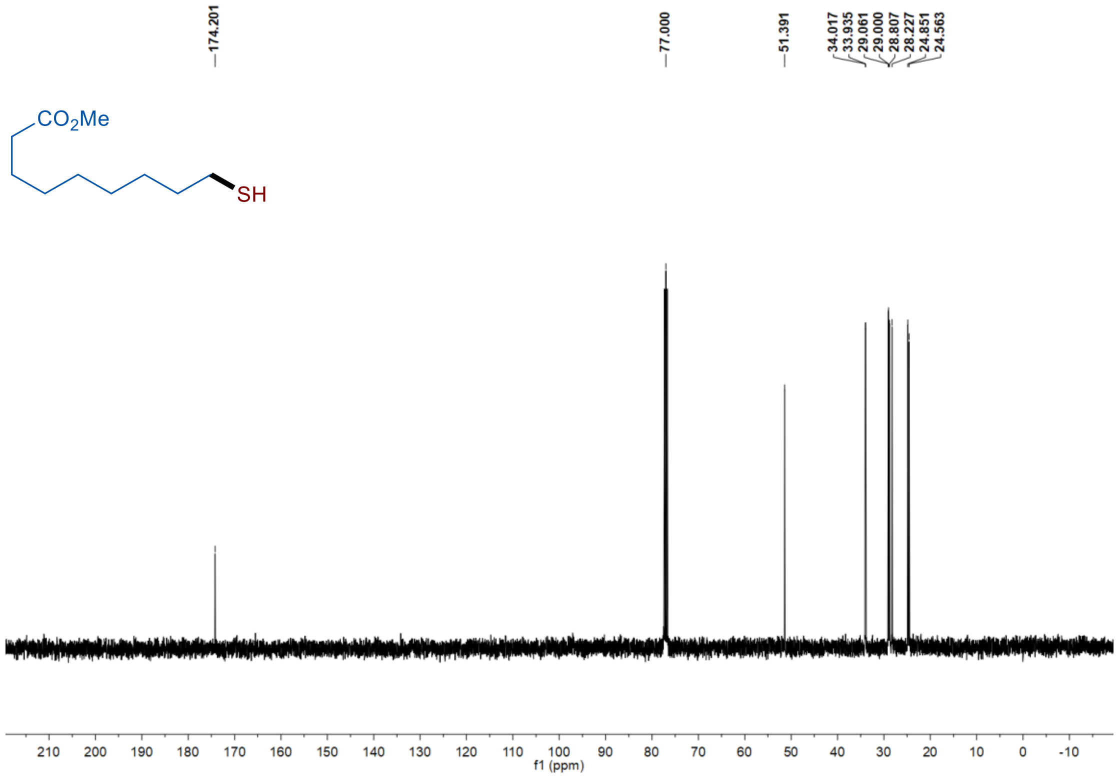

Supplementary Figure 16. ^{1}H and ^{13}C NMR spectra for compound **9**

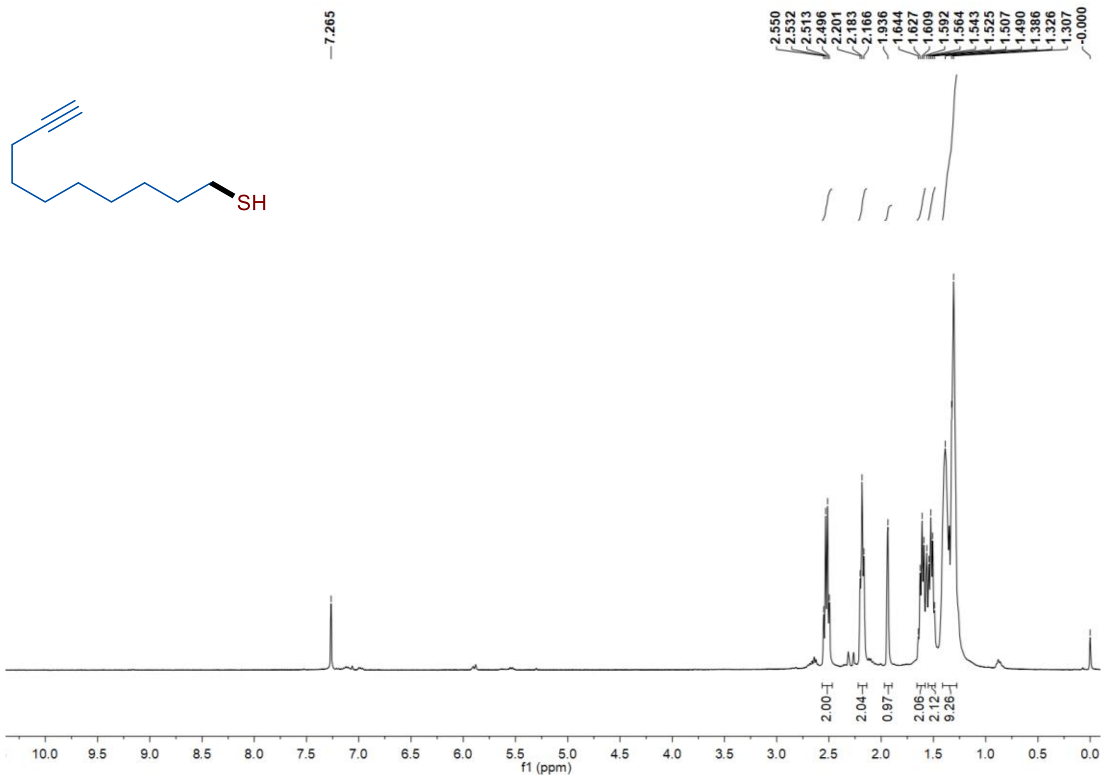

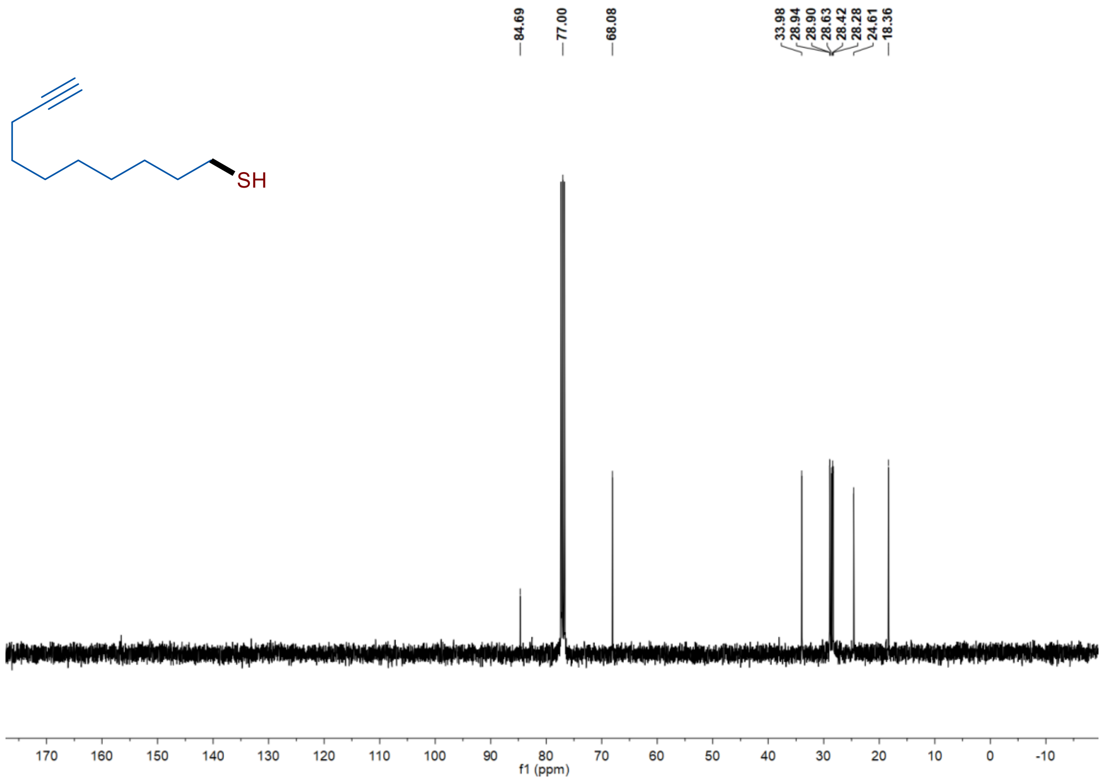

Supplementary Figure 17. ^{1}H and ^{13}C NMR spectra for compound **10**

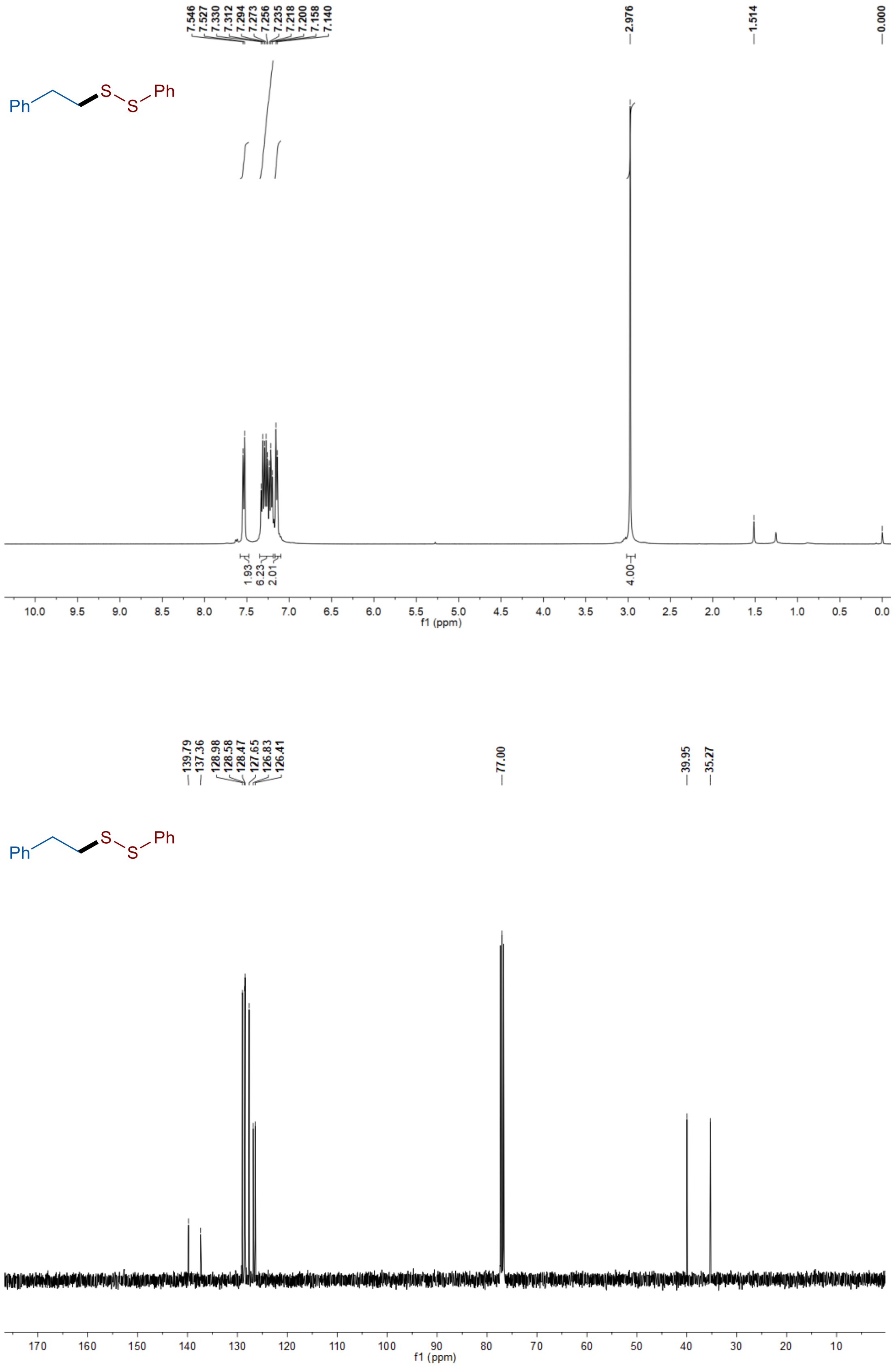

Supplementary Figure 18. ^{1}H and ^{13}C NMR spectra for compound **11**

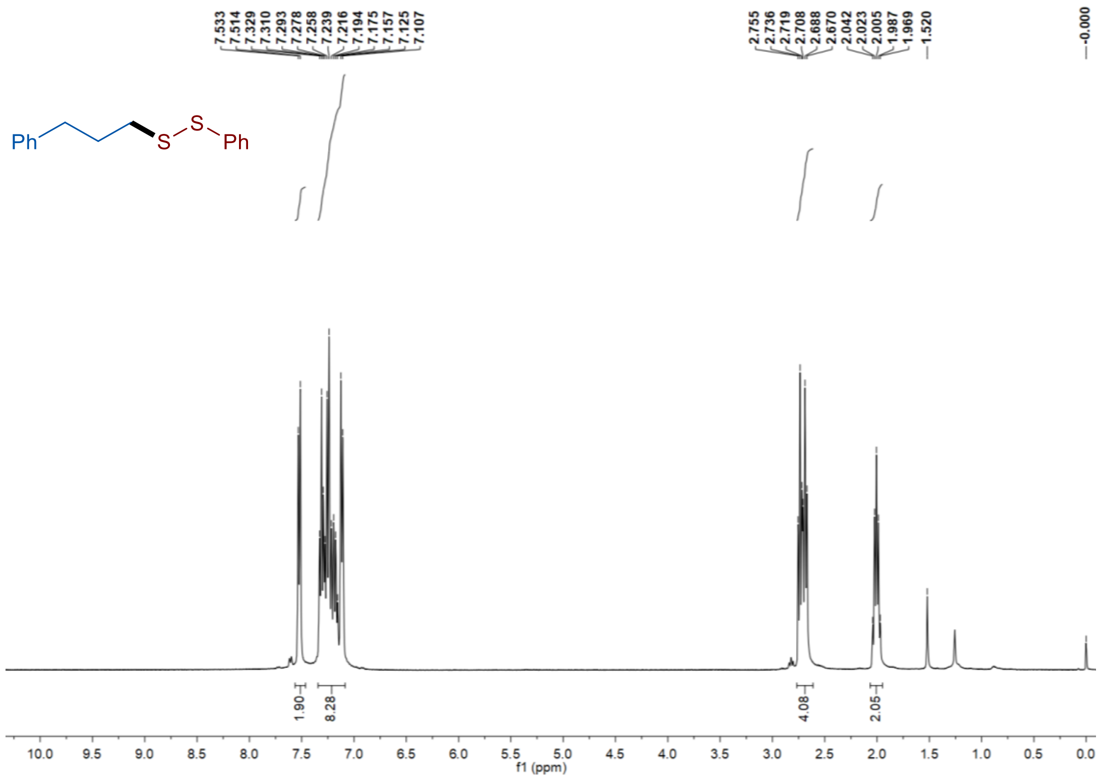

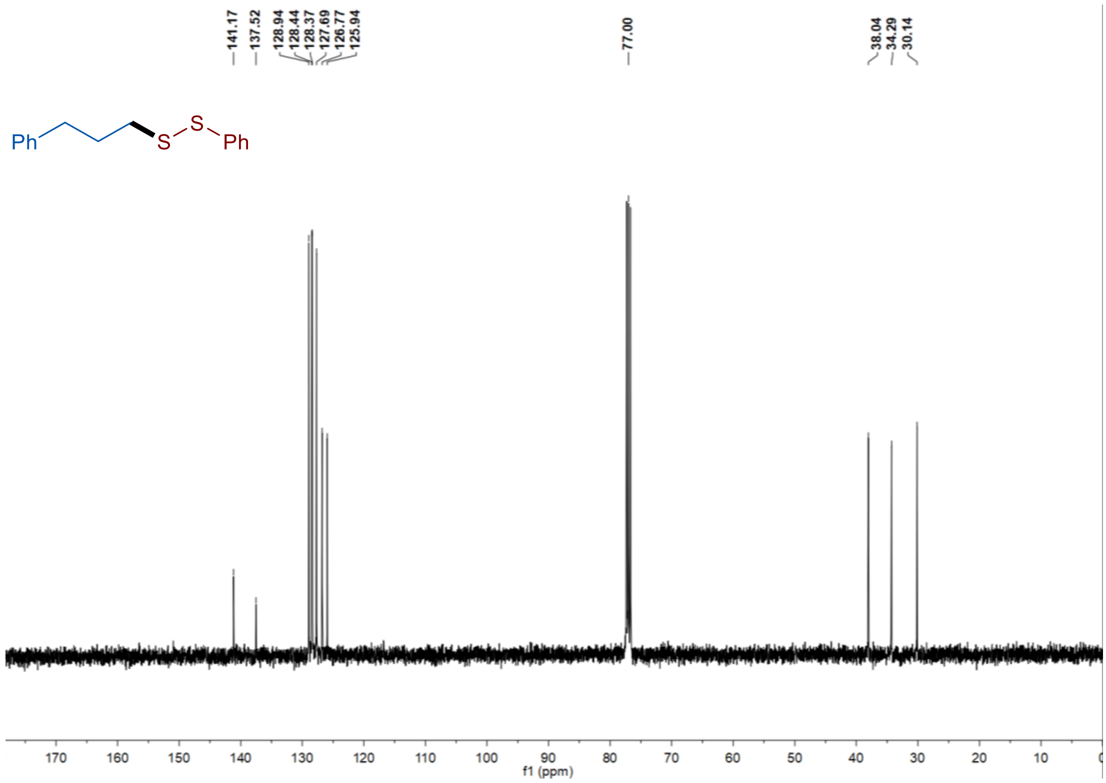

Supplementary Figure 19. ^{1}H and ^{13}C NMR spectra for compound **12**

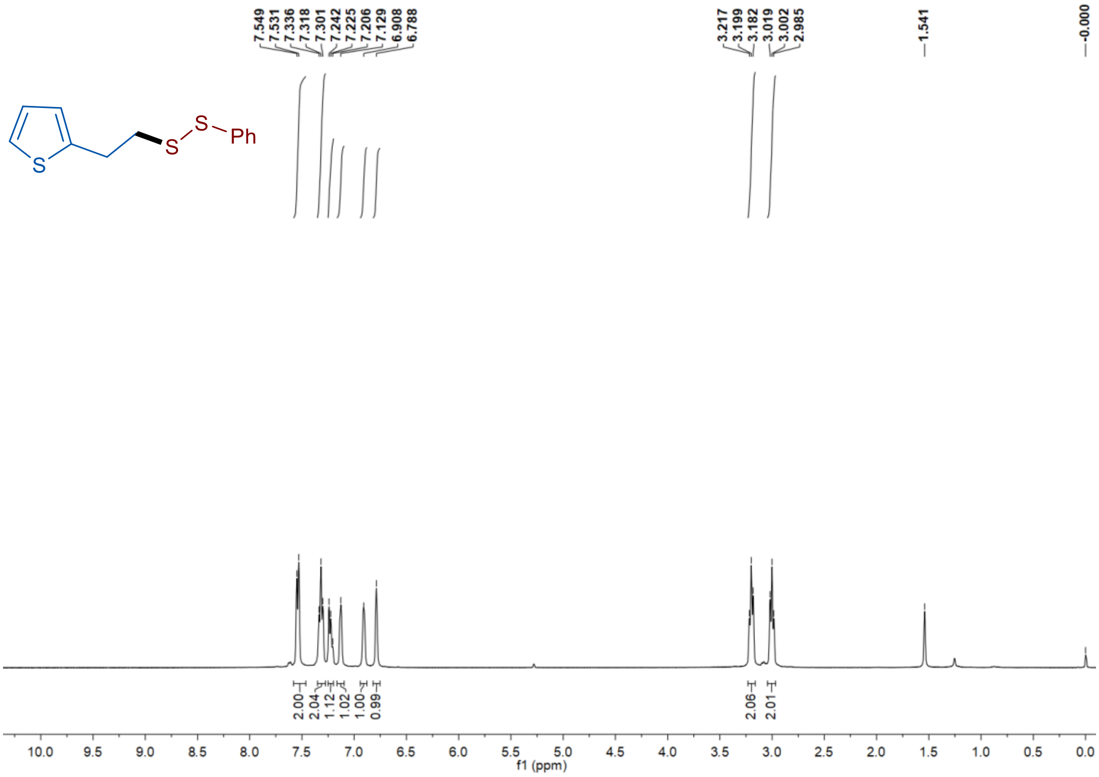

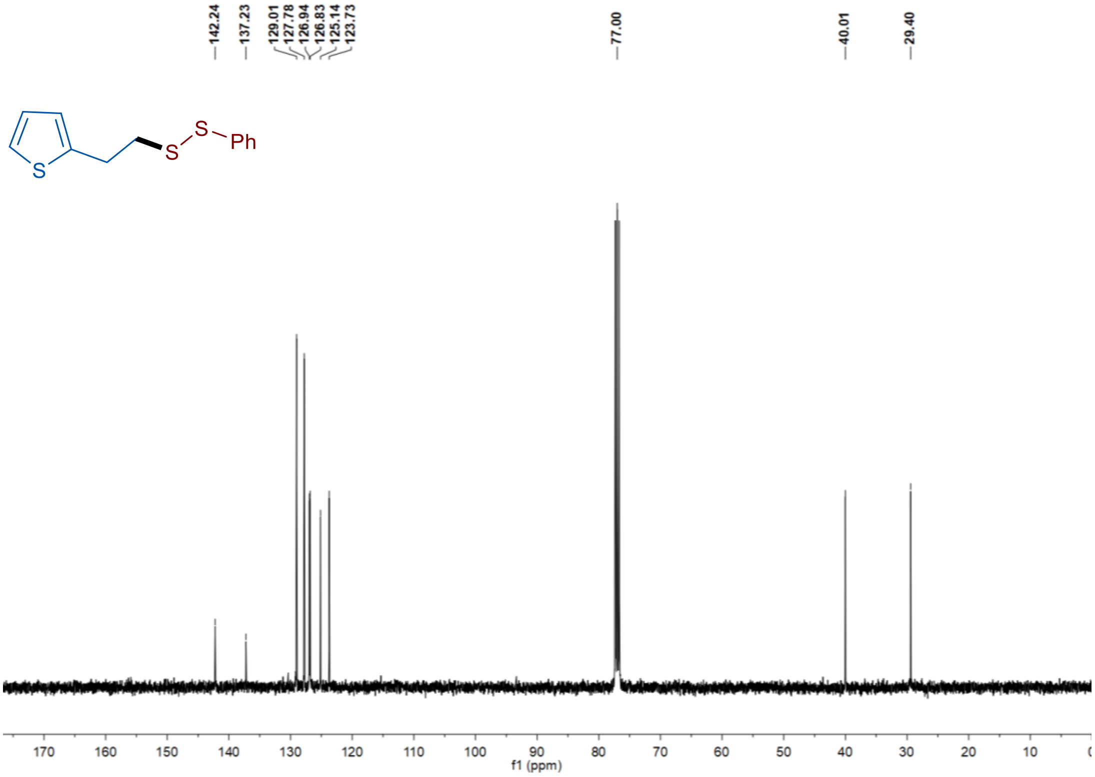

Supplementary Figure 20. ^{1}H and ^{13}C NMR spectra for compound **13**

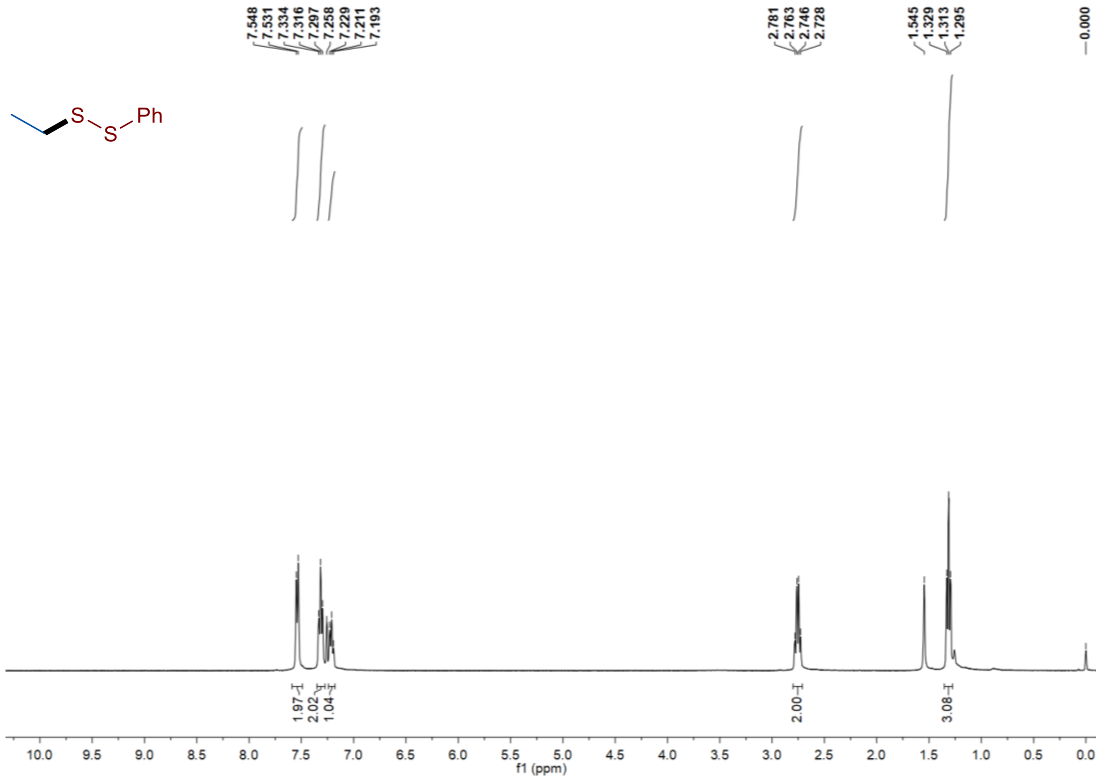

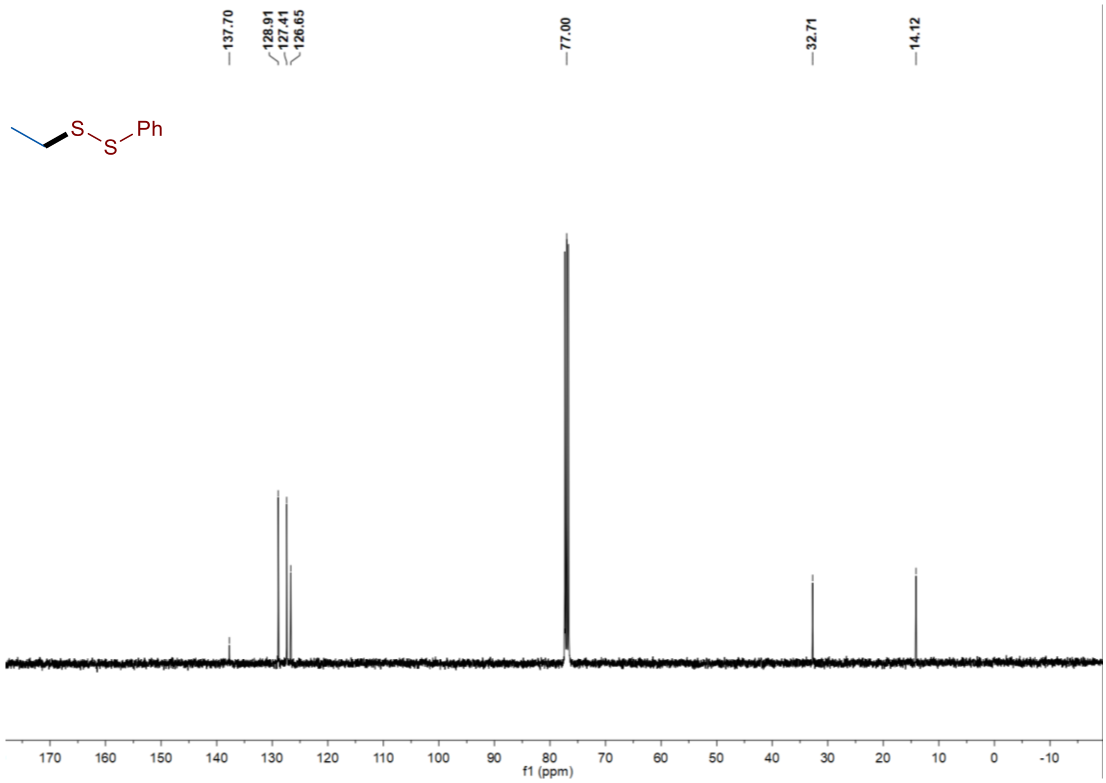

Supplementary Figure 21. ^{1}H and ^{13}C NMR spectra for compound **14**

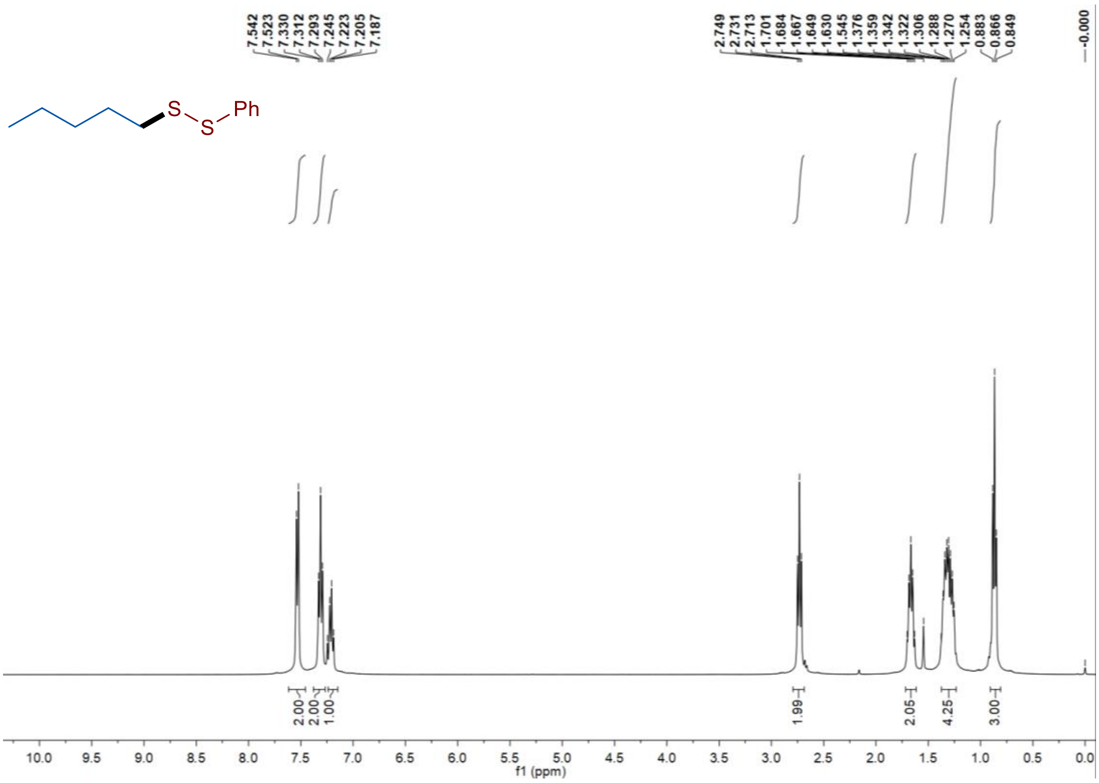

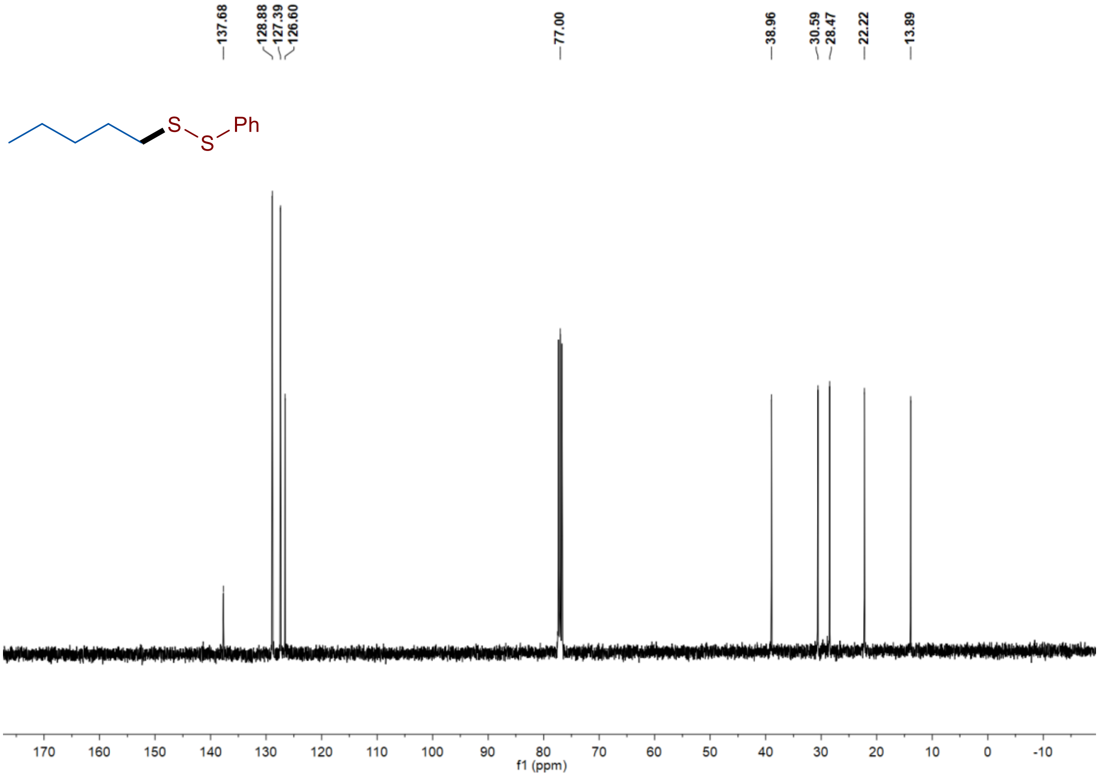

Supplementary Figure 22. ^{1}H and ^{13}C NMR spectra for compound **15**

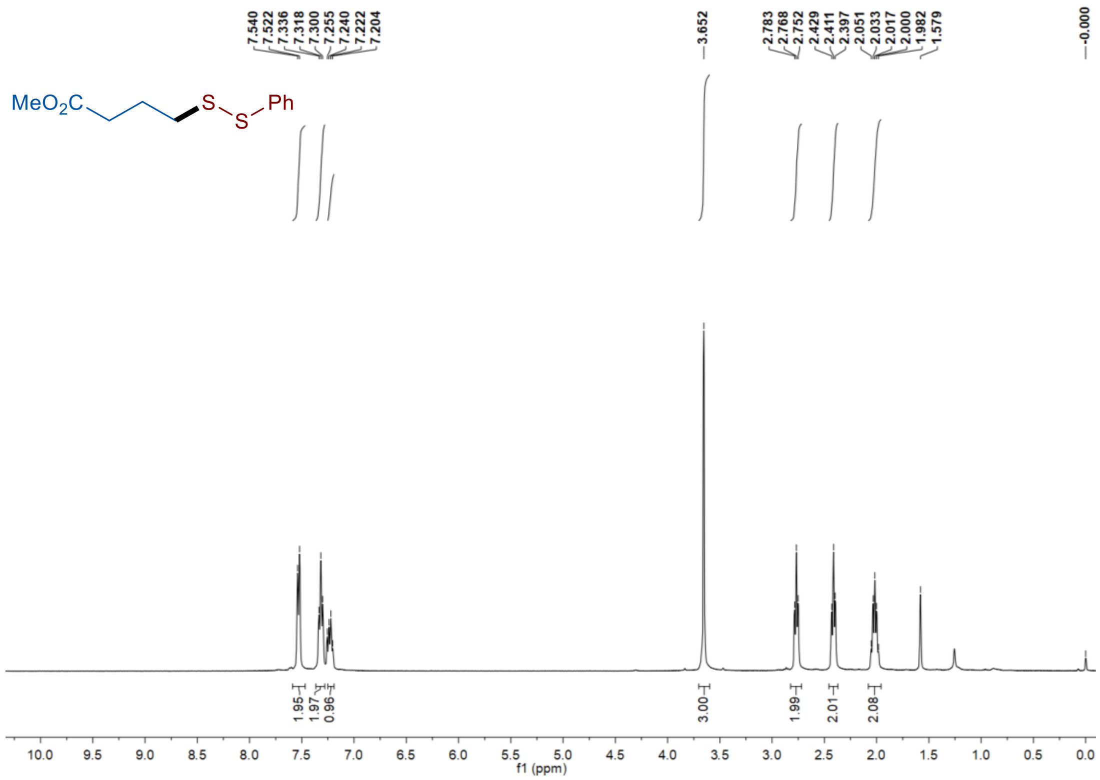

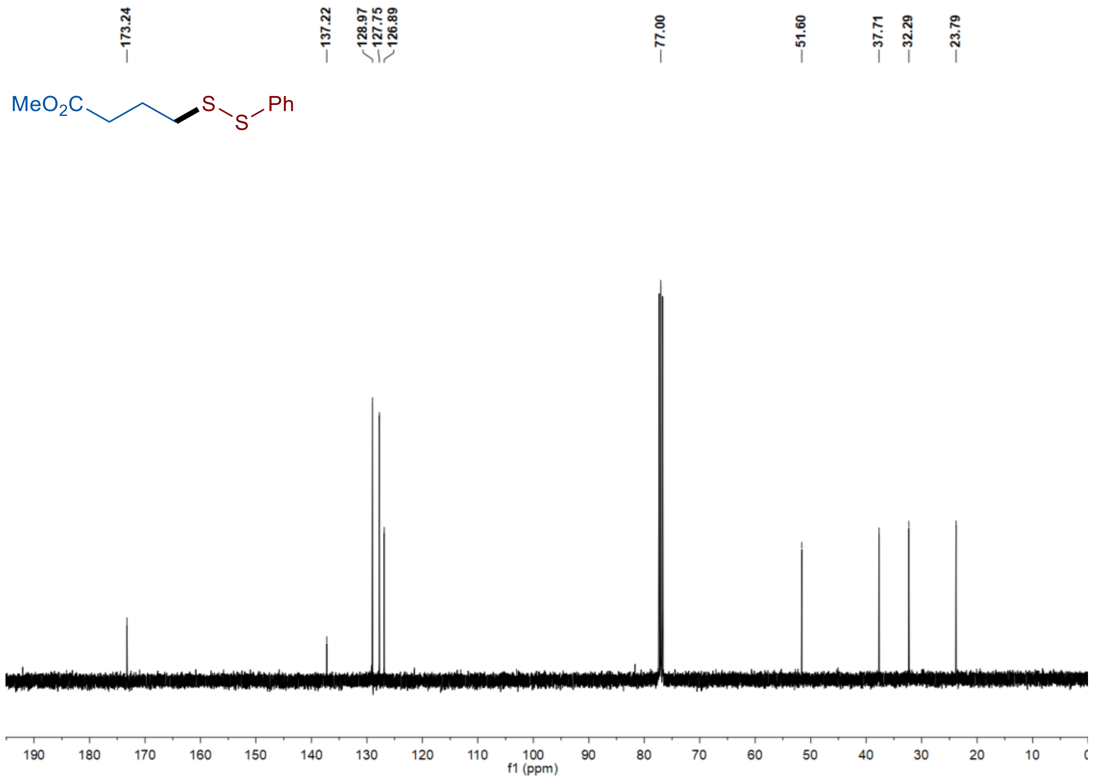

Supplementary Figure 23. ^{1}H and ^{13}C NMR spectra for compound **16**

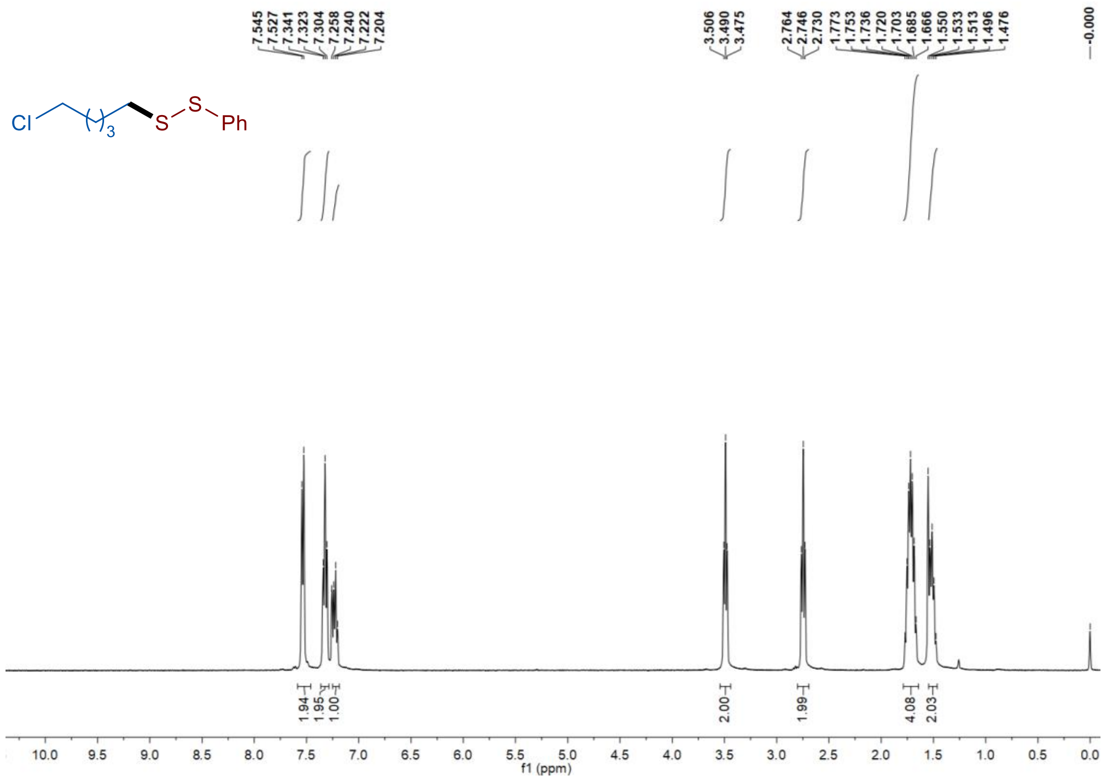

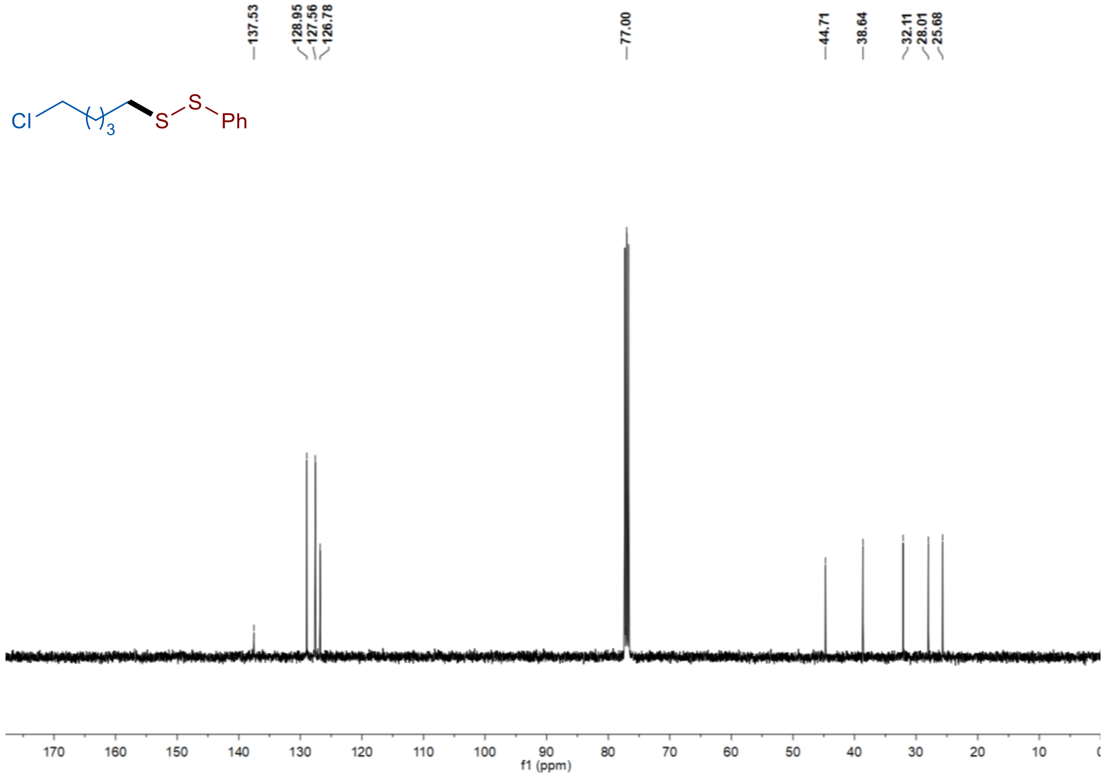

Supplementary Figure 24. ^{1}H and ^{13}C NMR spectra for compound **17**

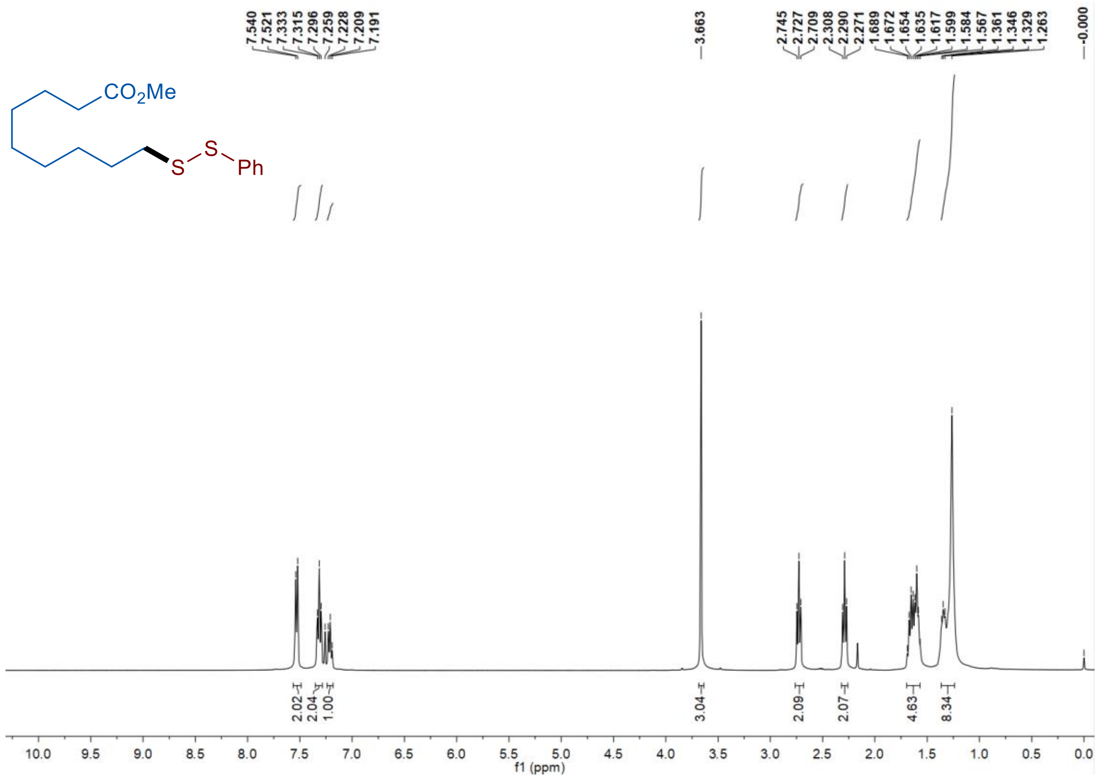

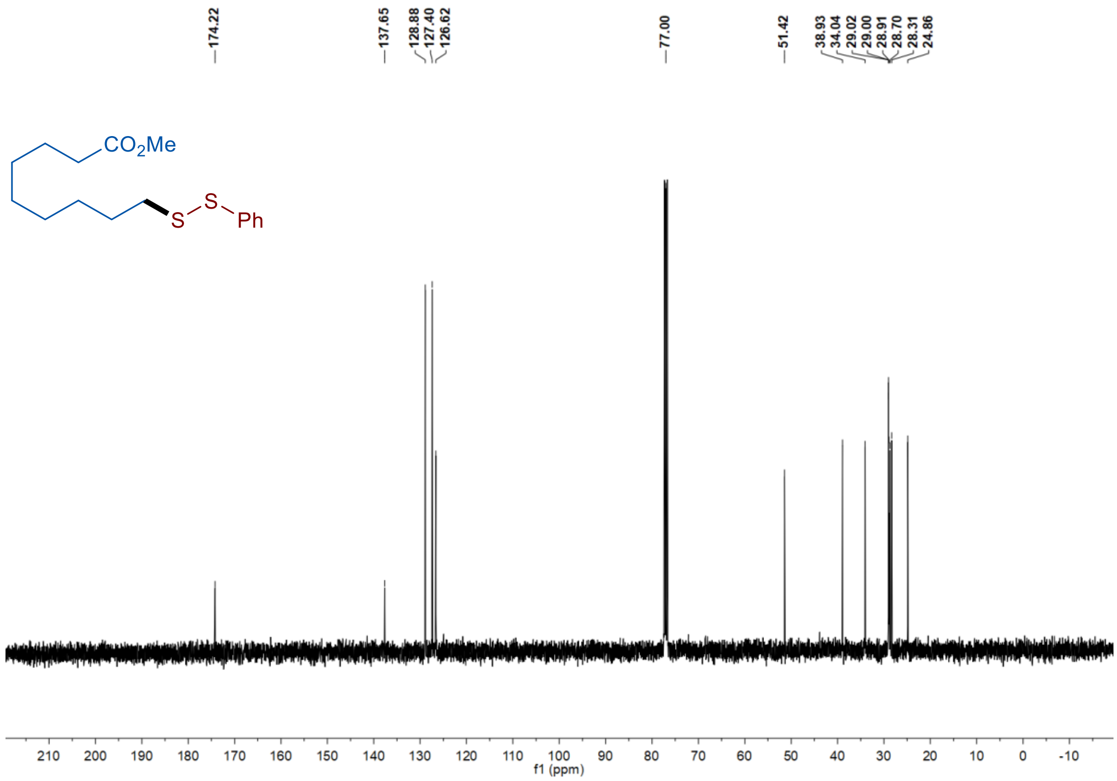

Supplementary Figure 25. ^{1}H and ^{13}C NMR spectra for compound **18**

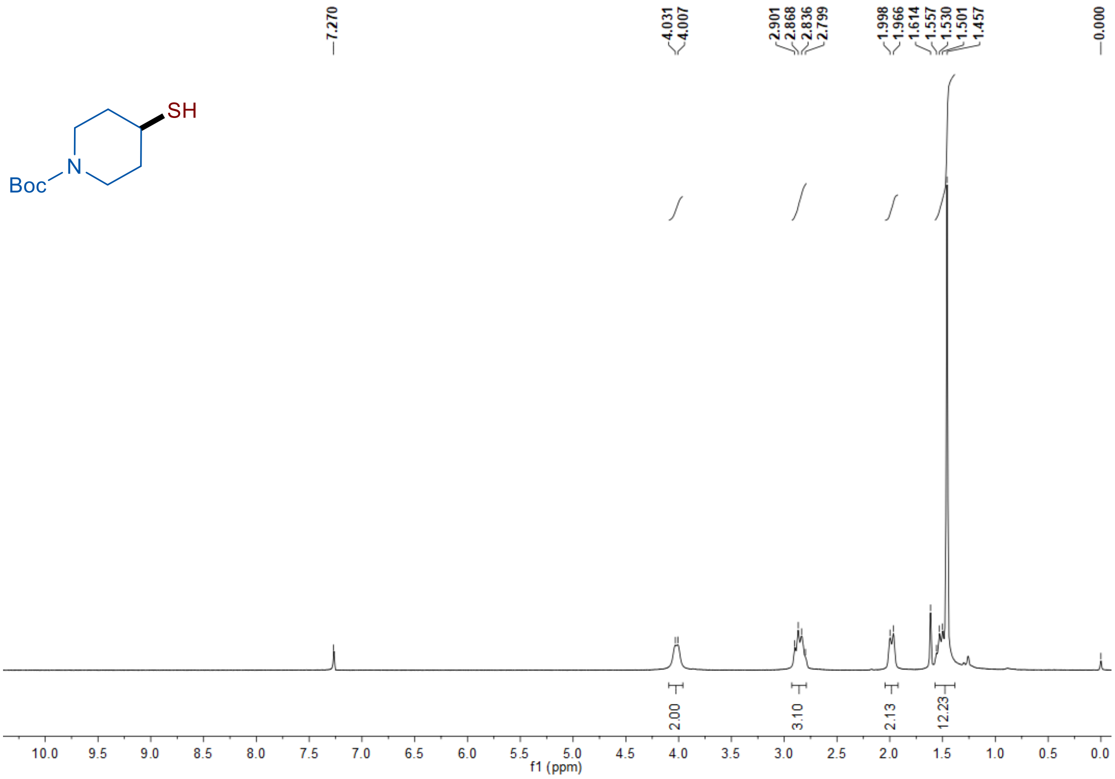

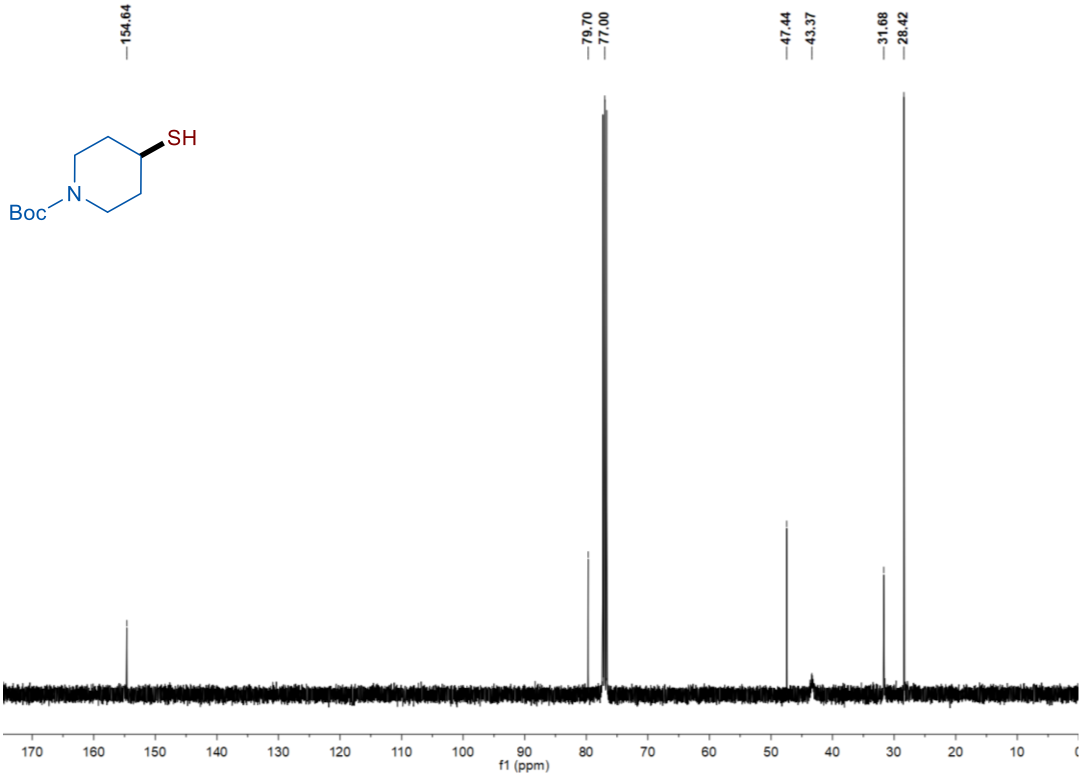

Supplementary Figure 26. ^{1}H and ^{13}C NMR spectra for compound **19**

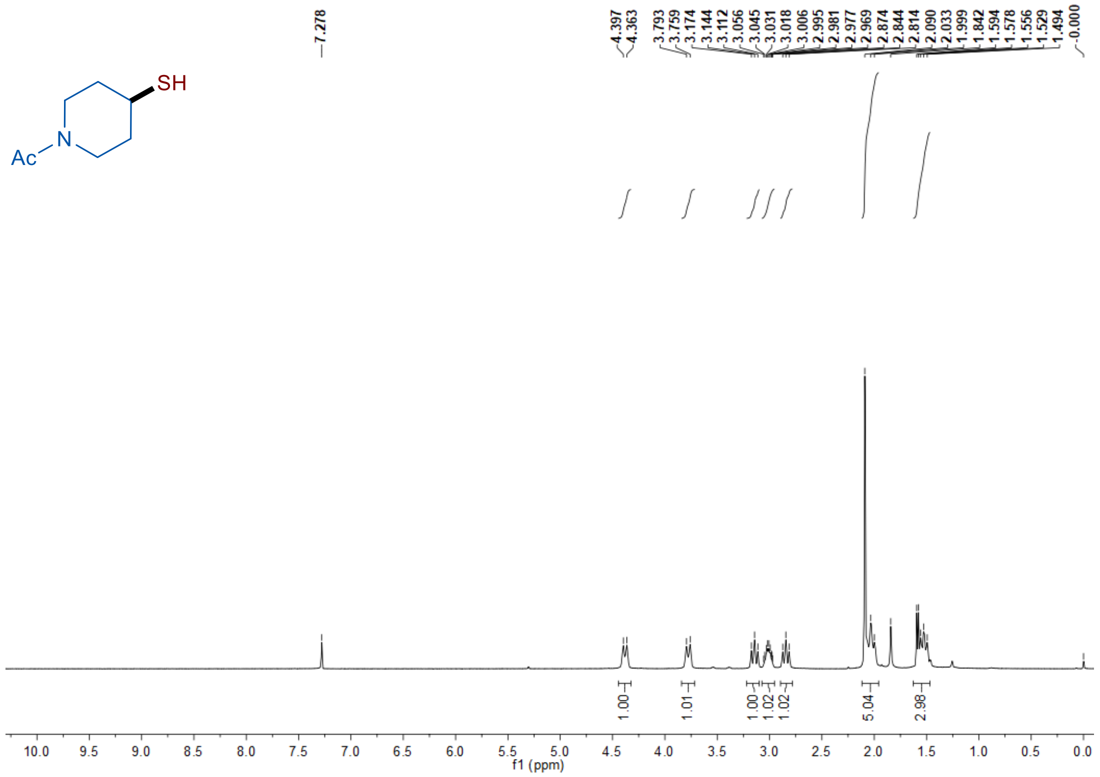

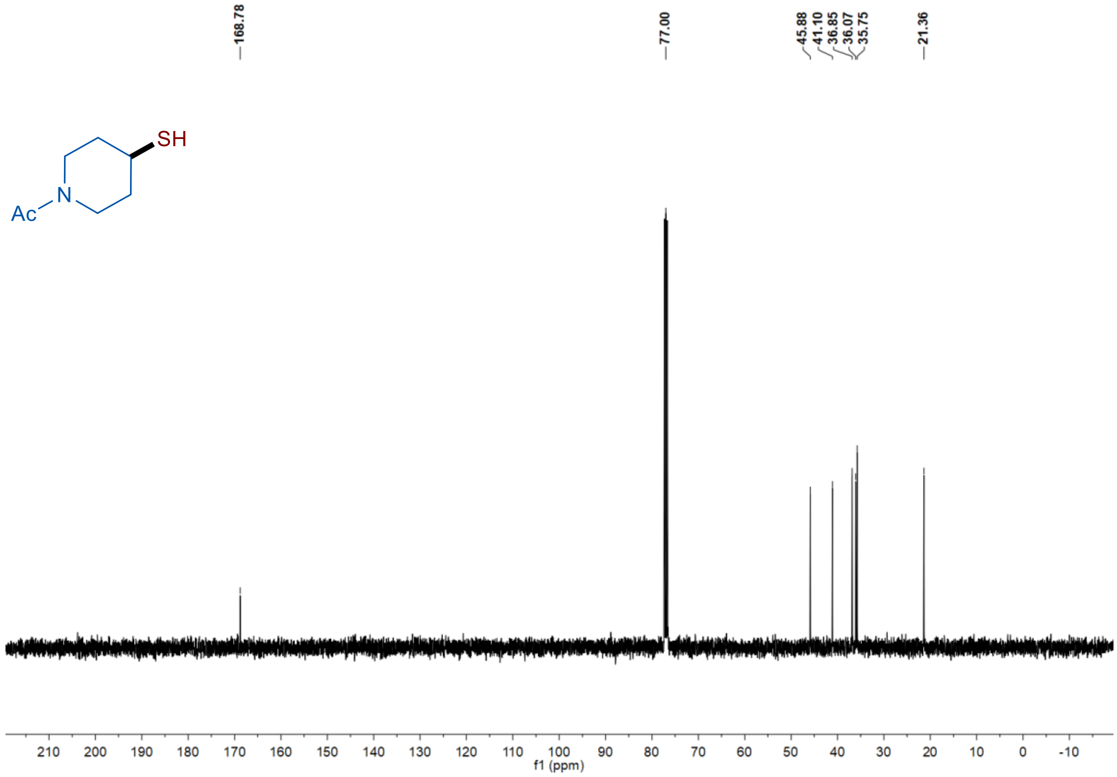

Supplementary Figure 27. ^{1}H and ^{13}C NMR spectra for compound **20**

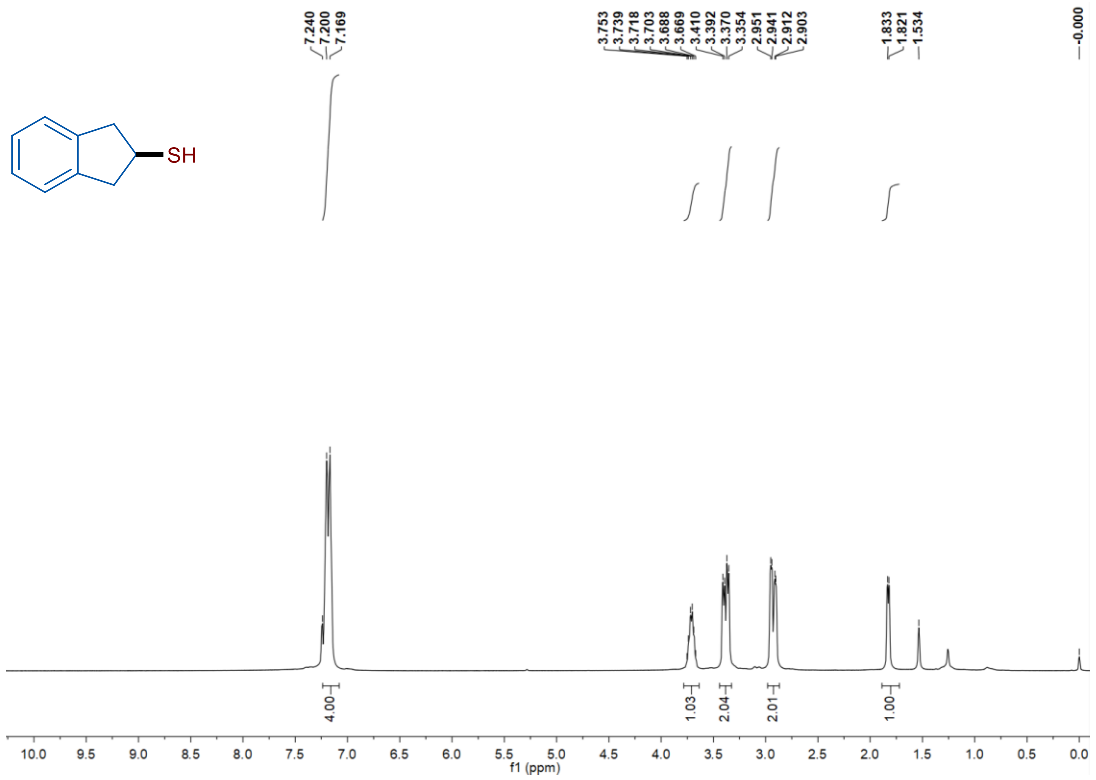

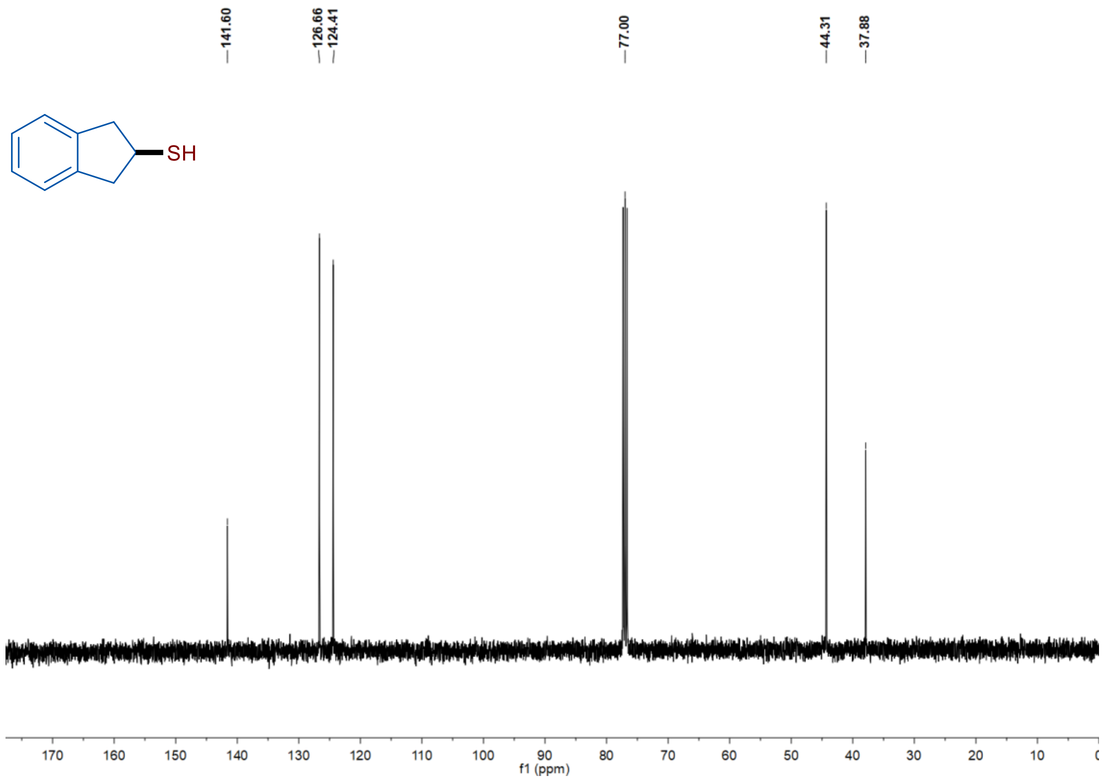

Supplementary Figure 28. ^{1}H and ^{13}C NMR spectra for compound **21**

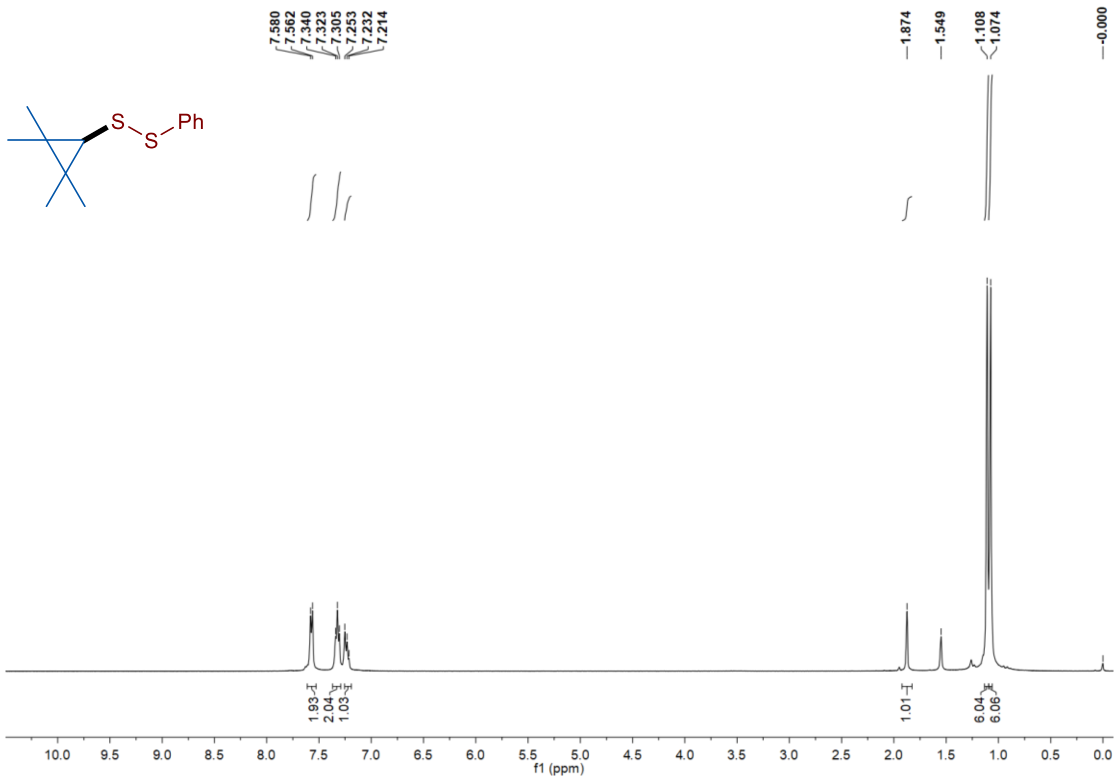

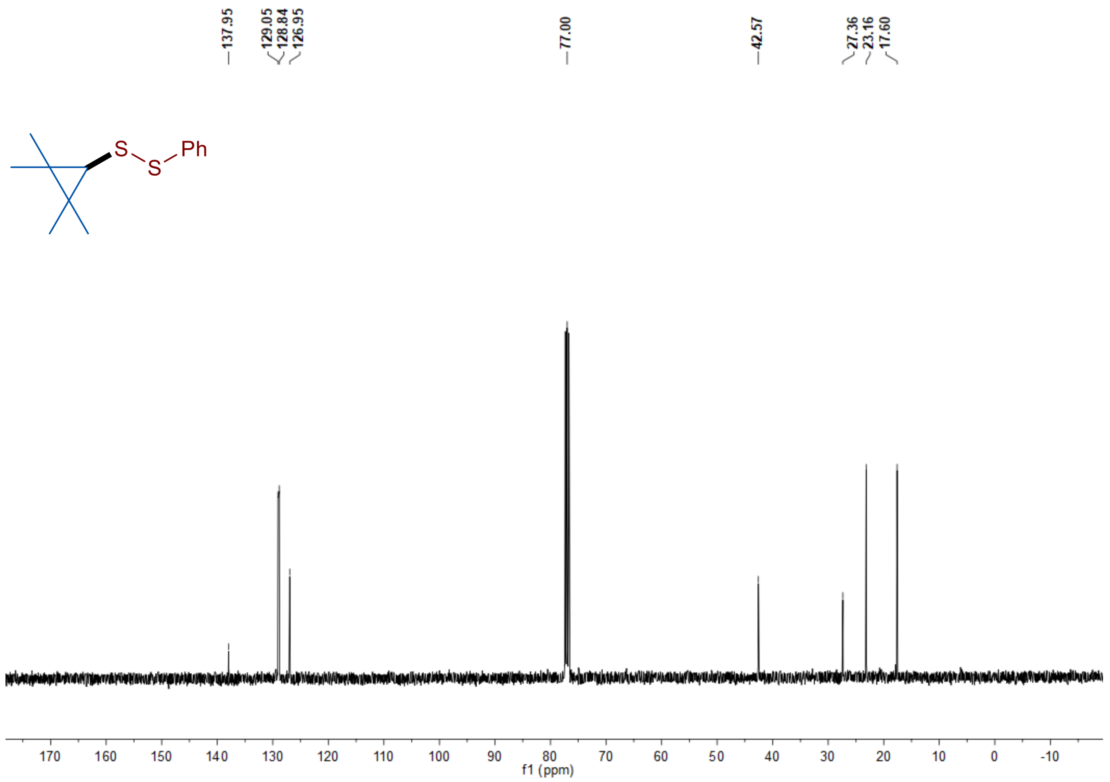

Supplementary Figure 29. ^{1}H and ^{13}C NMR spectra for compound **22**

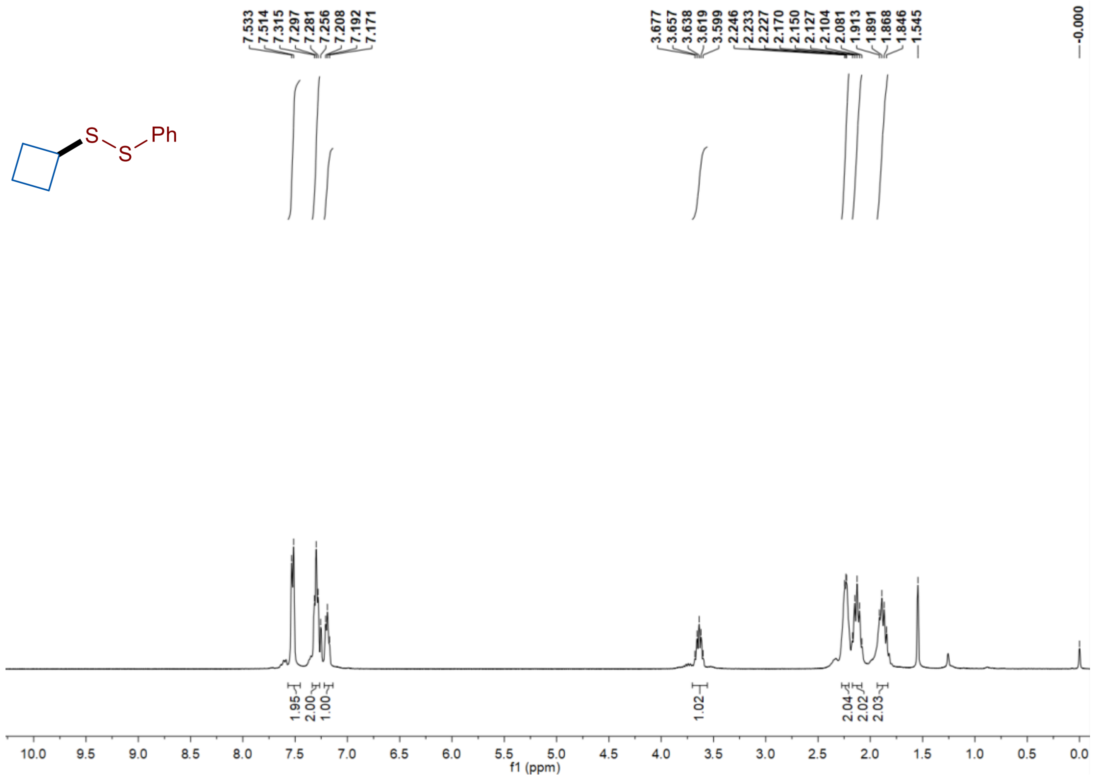

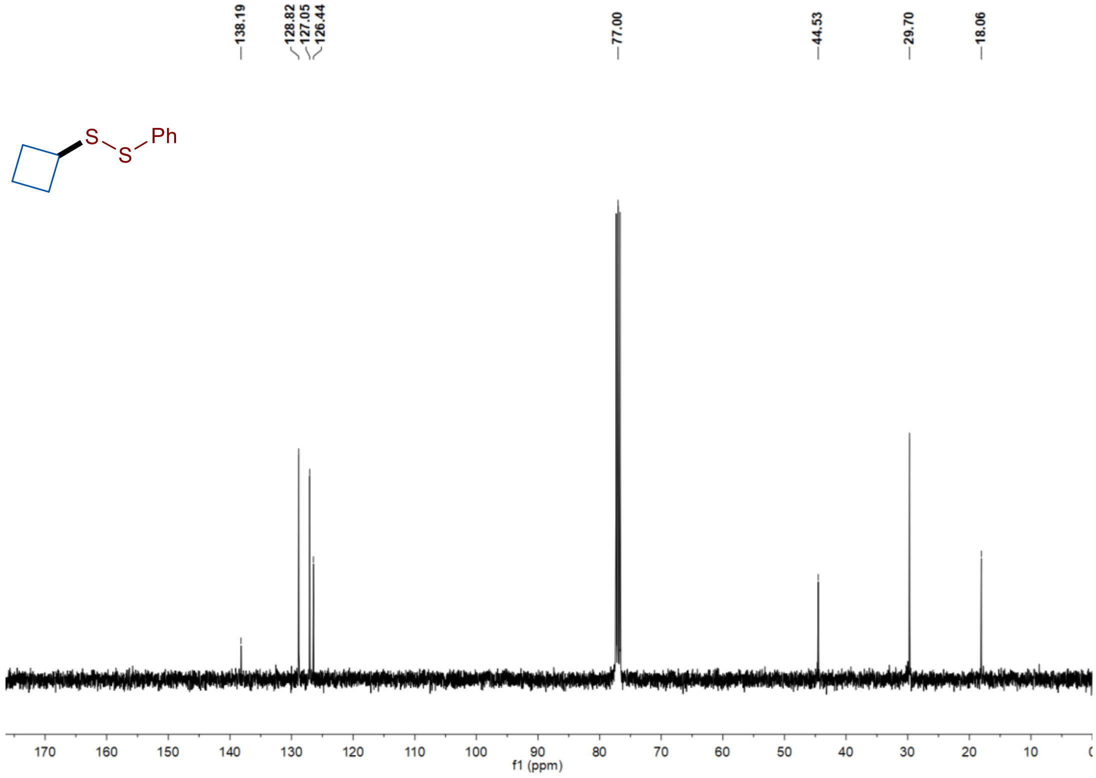

Supplementary Figure 30. ^{1}H and ^{13}C NMR spectra for compound **23**

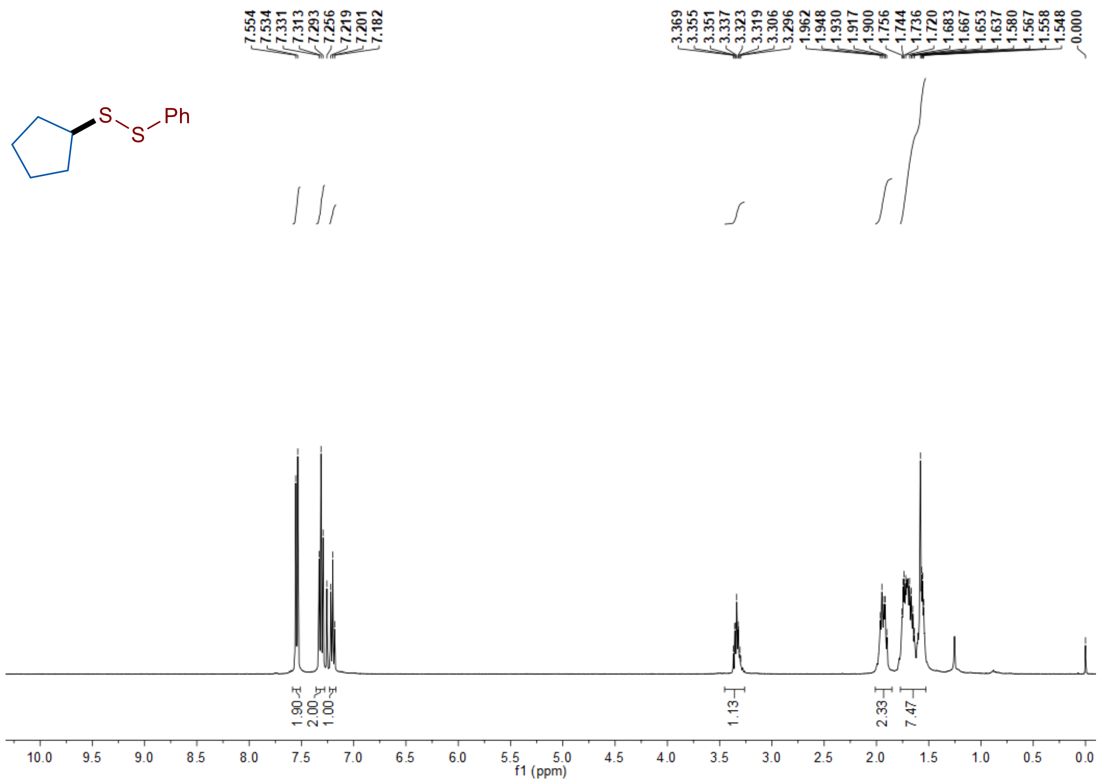

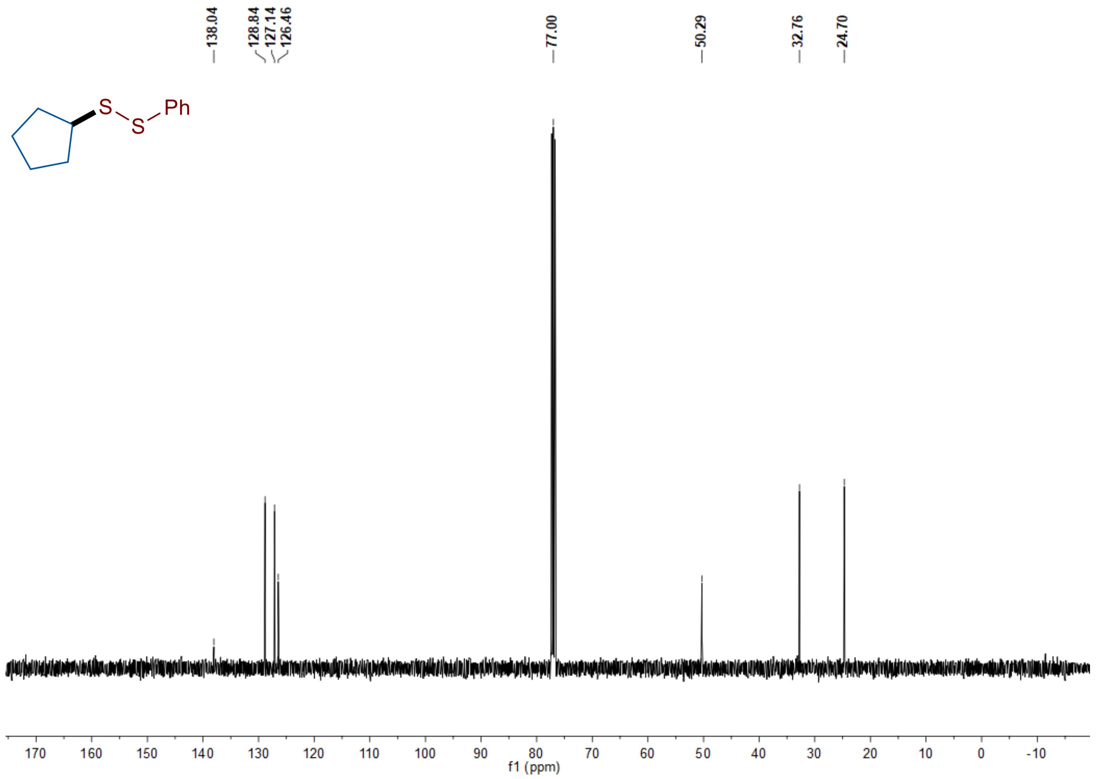

Supplementary Figure 31. ^{1}H and ^{13}C NMR spectra for compound **24**

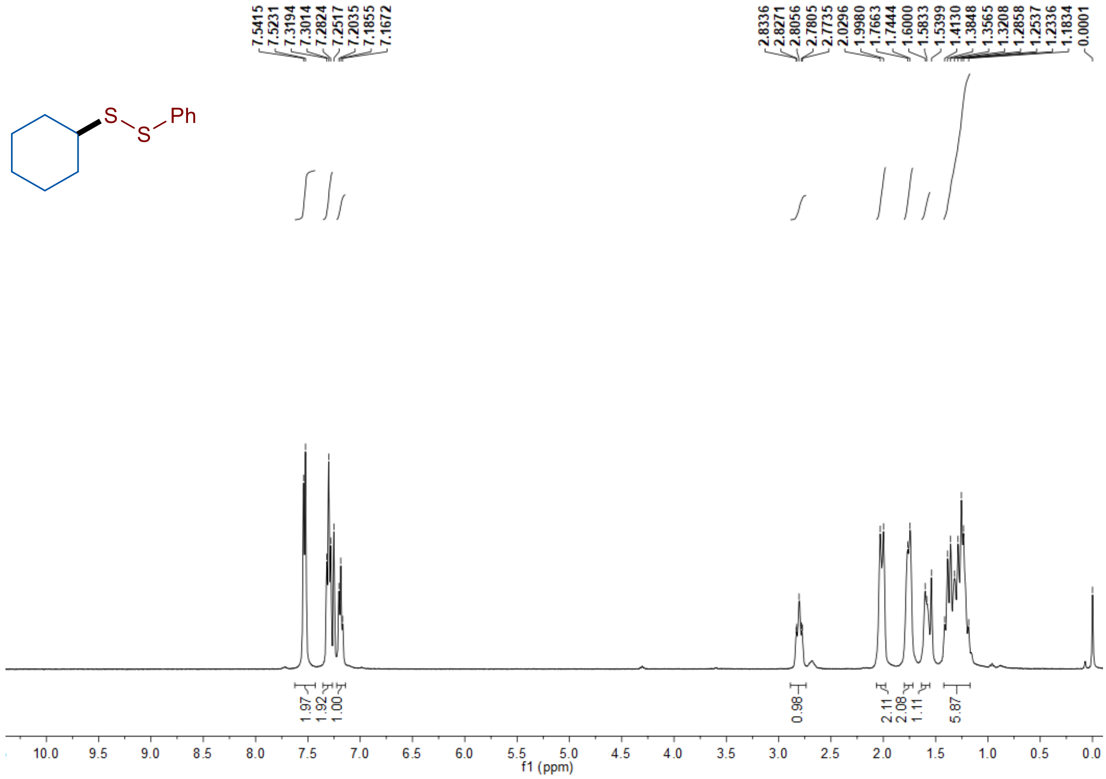

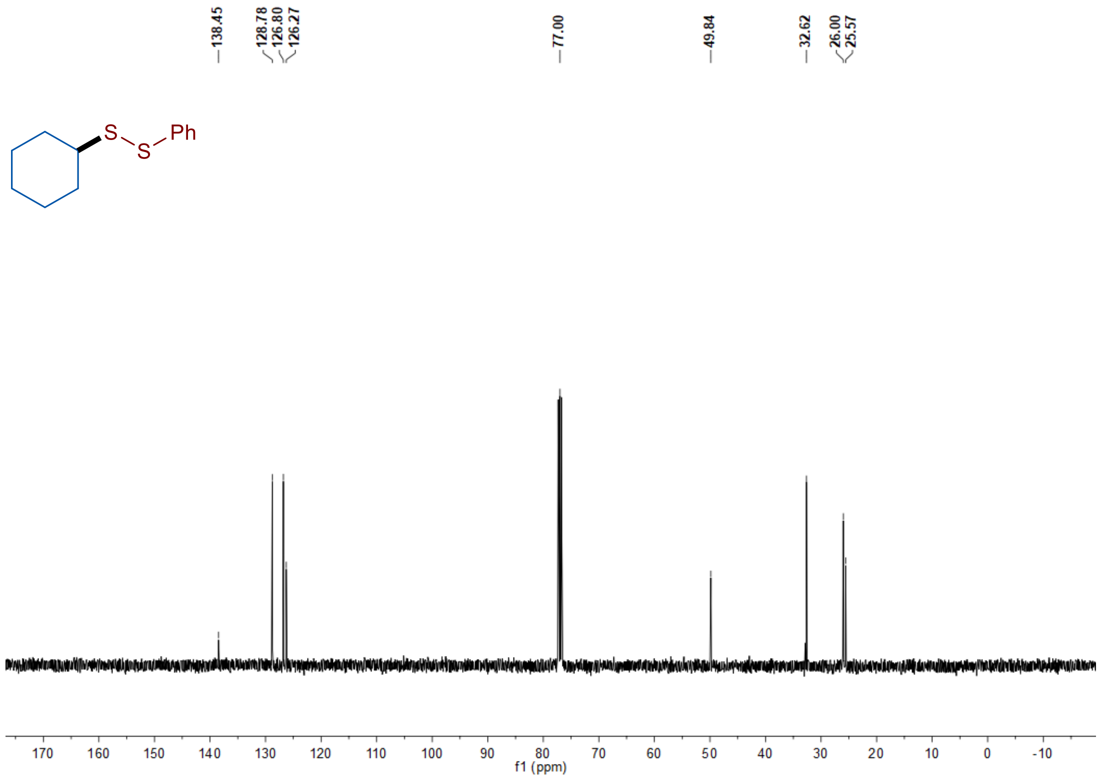

Supplementary Figure 32. ^{1}H and ^{13}C NMR spectra for compound **25**

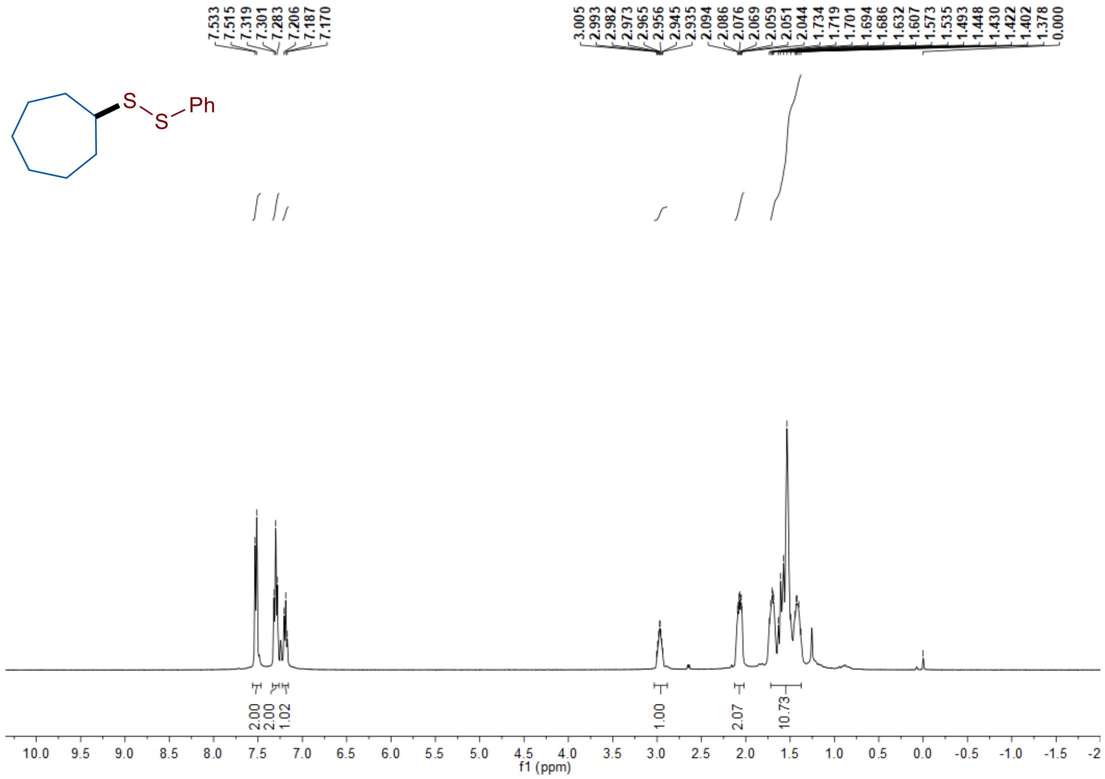

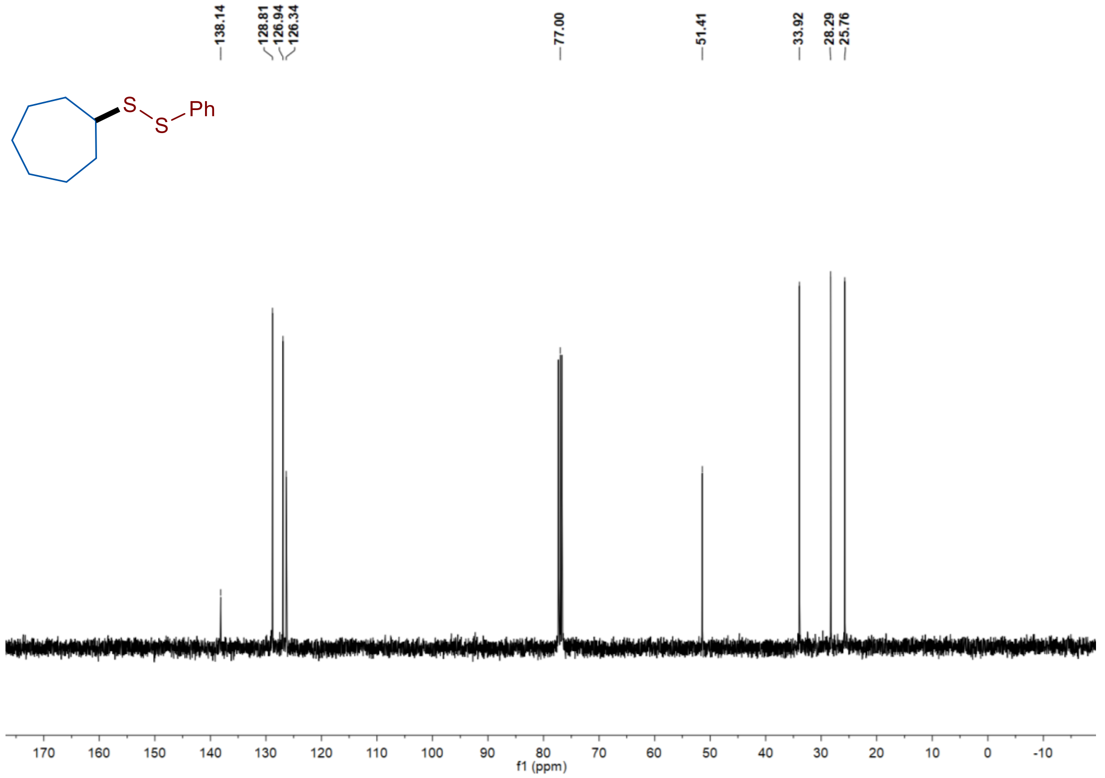

Supplementary Figure 33. ^{1}H and ^{13}C NMR spectra for compound **26**

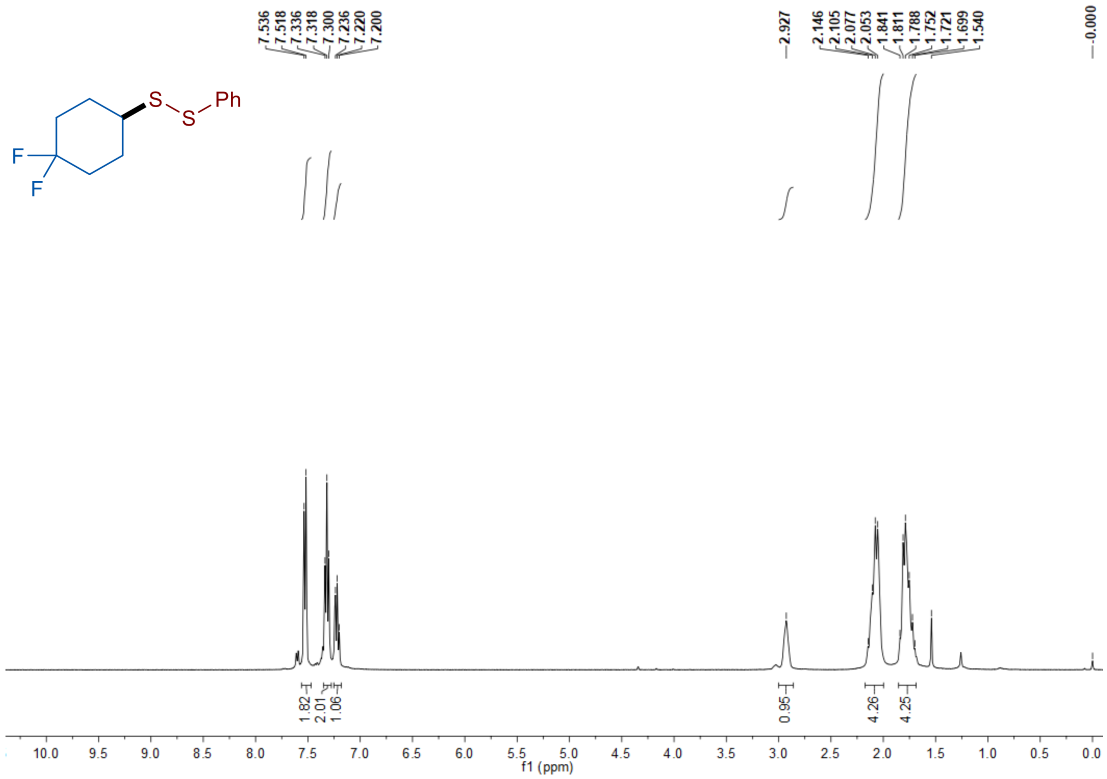

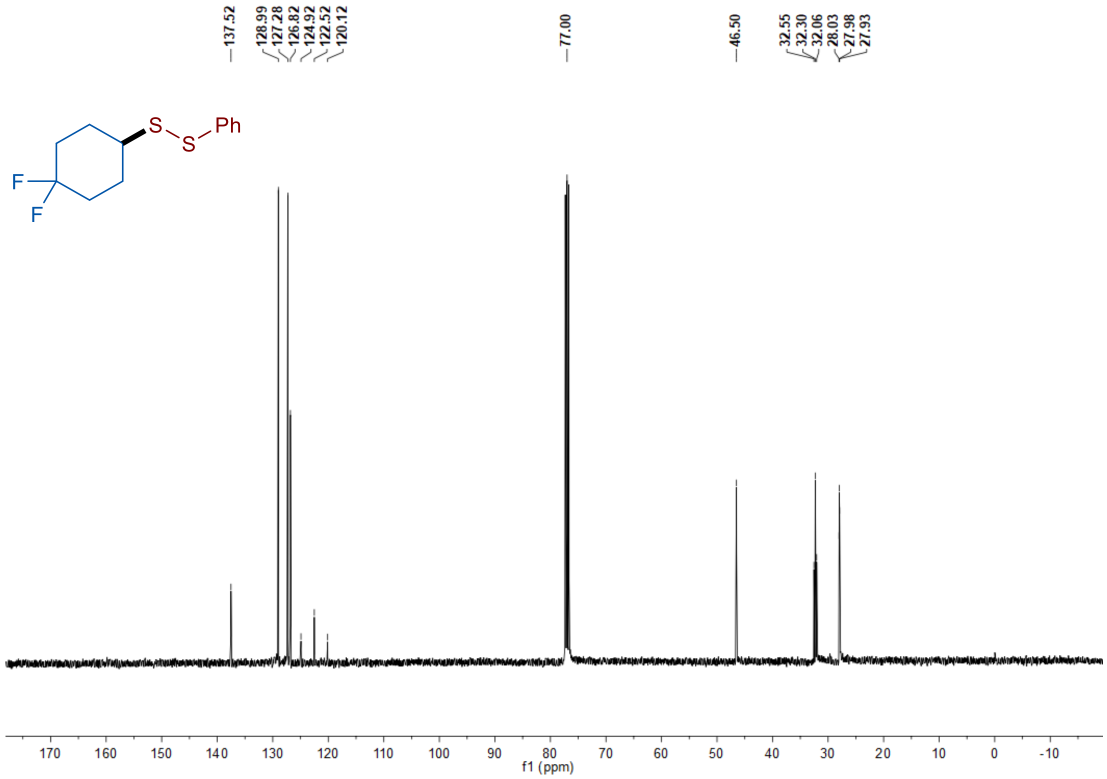

Supplementary Figure 34. ^{1}H and ^{13}C NMR spectra for compound **27**

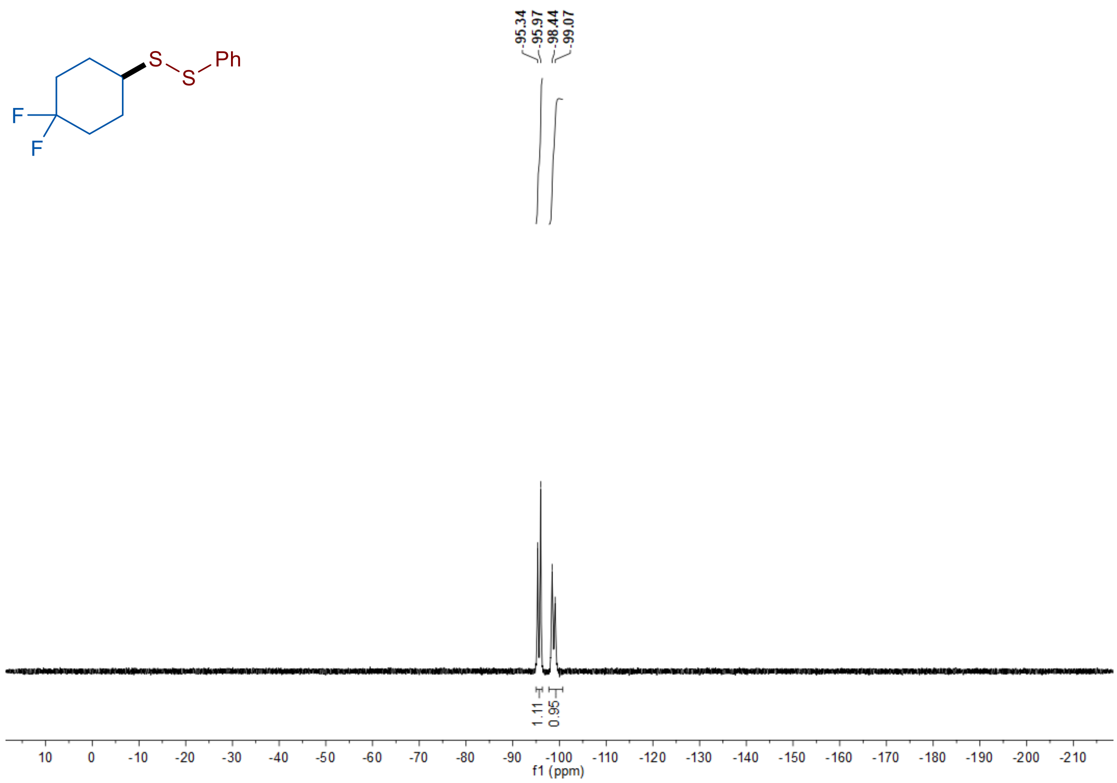

Supplementary Figure 35. ^{19}F NMR spectra for compound **27**

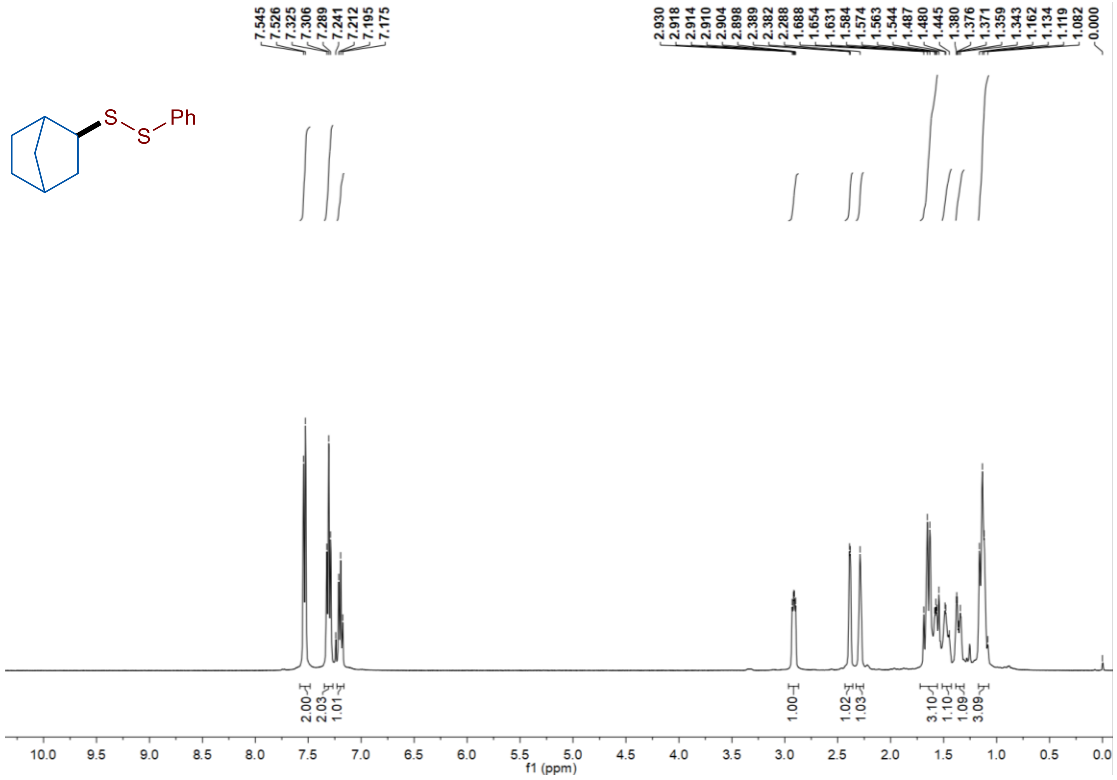

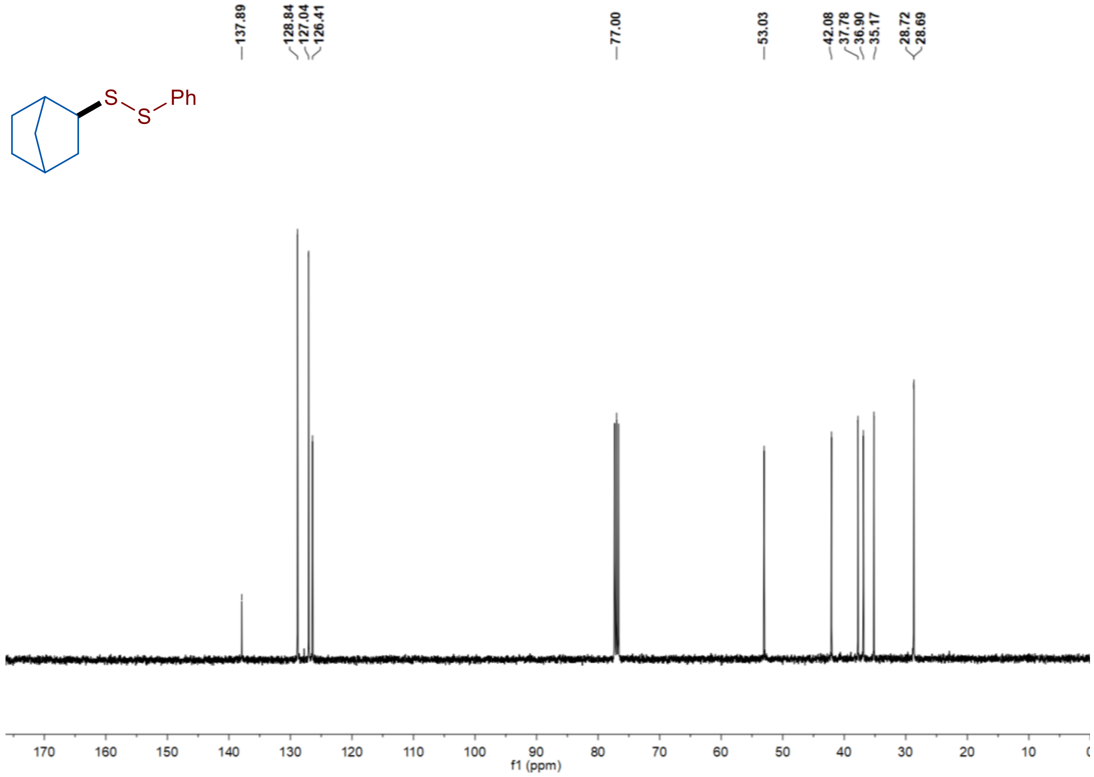

Supplementary Figure 36. ^{1}H and ^{13}C NMR spectra for compound **28**

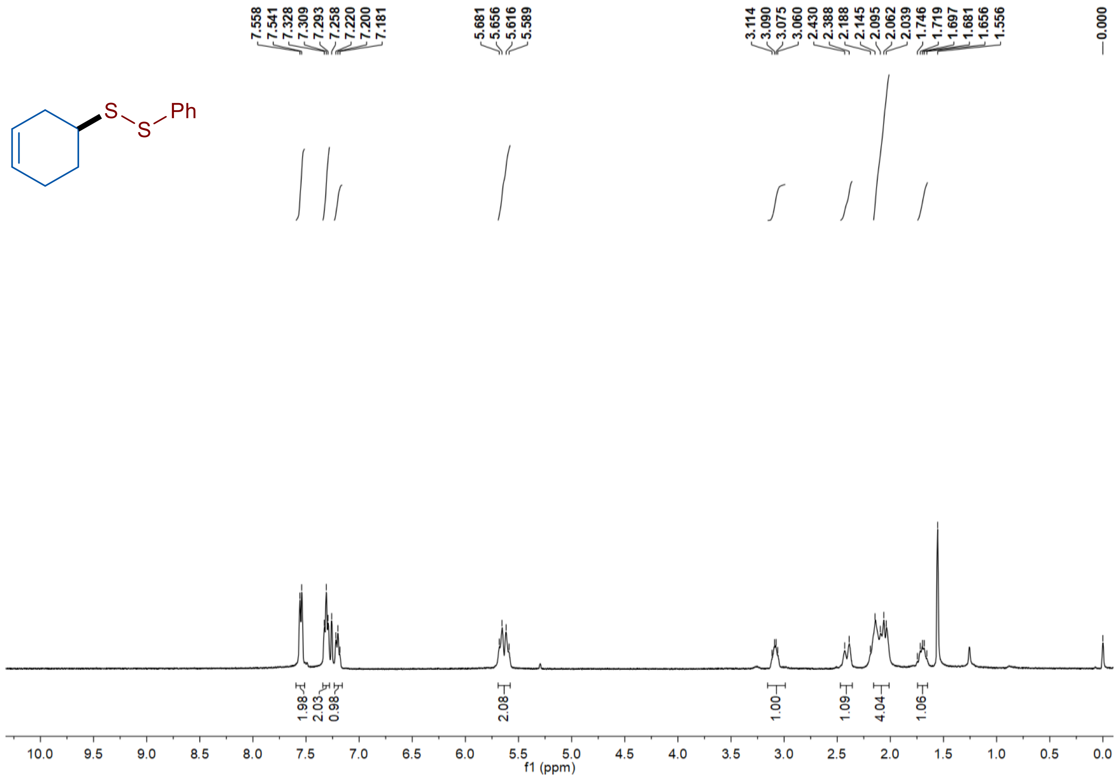

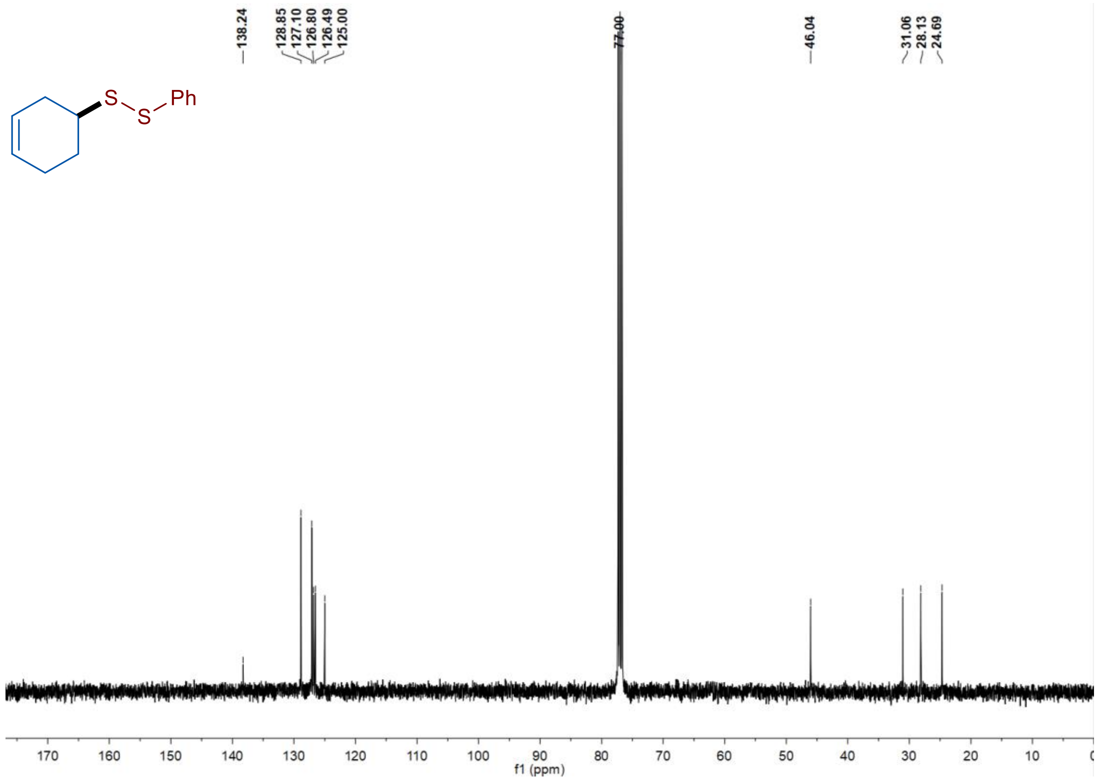

Supplementary Figure 37. ^{1}H and ^{13}C NMR spectra for compound **29**

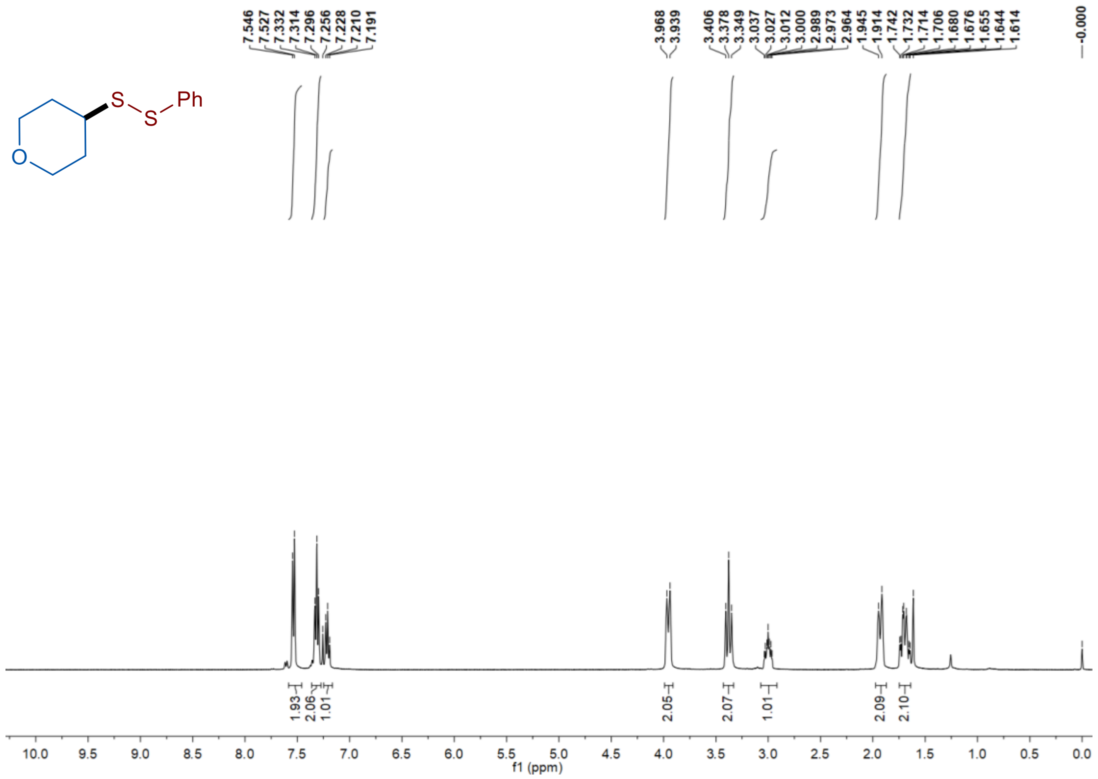

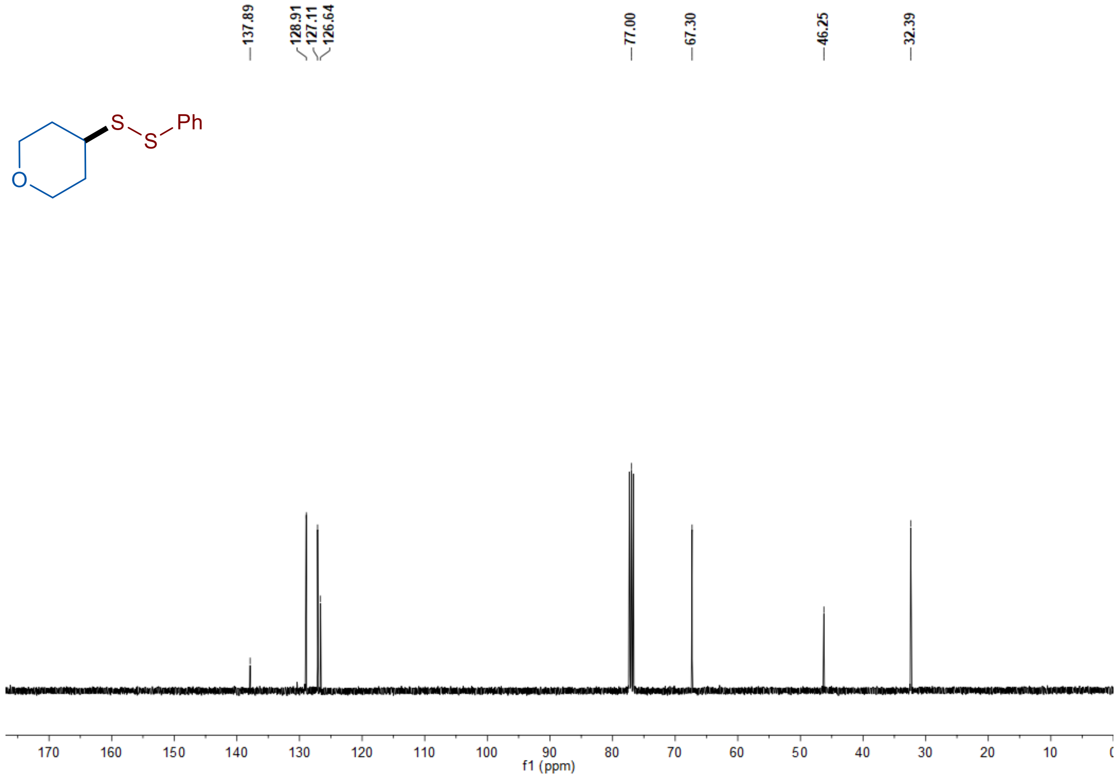

Supplementary Figure 38. ^{1}H and ^{13}C NMR spectra for compound **30**

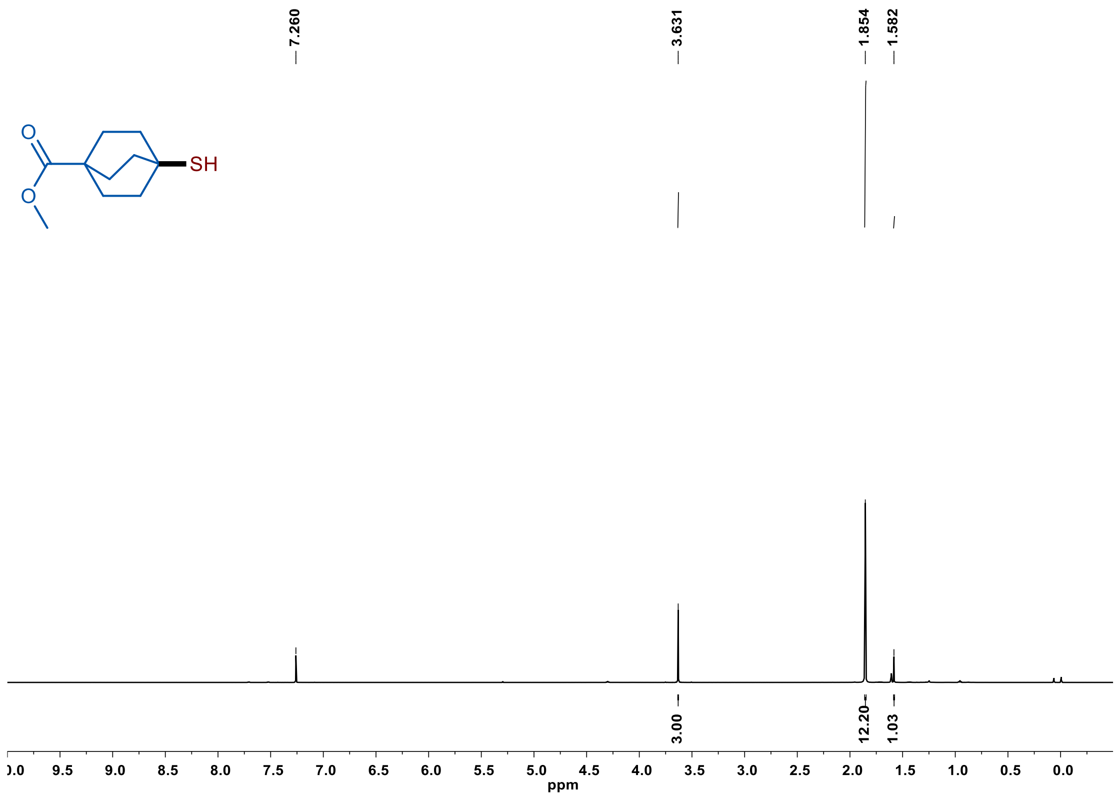

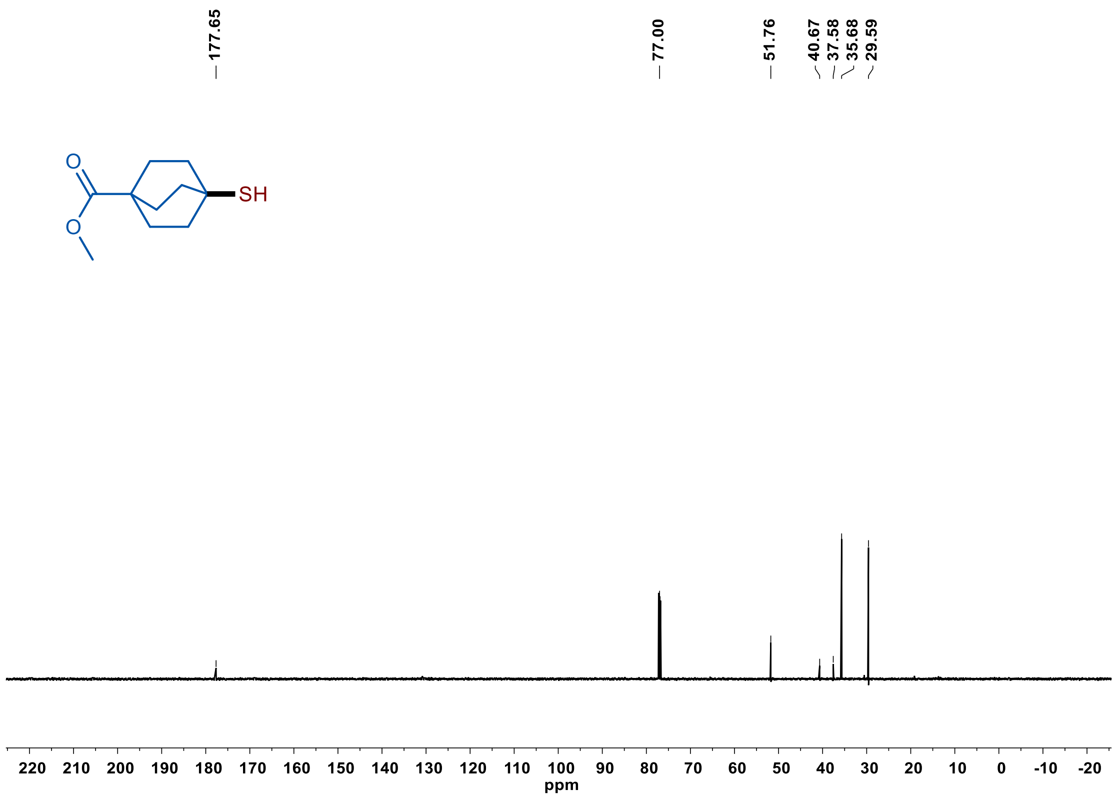

Supplementary Figure 39. ^{1}H and ^{13}C NMR spectra for compound **31**

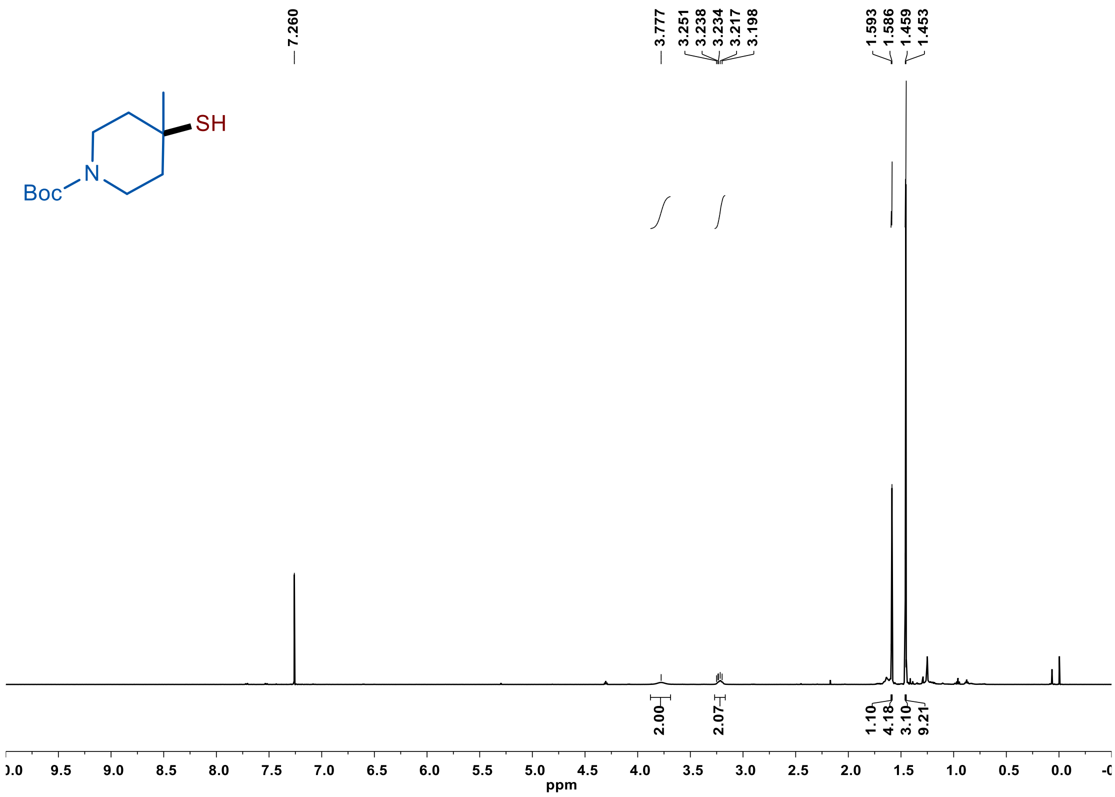

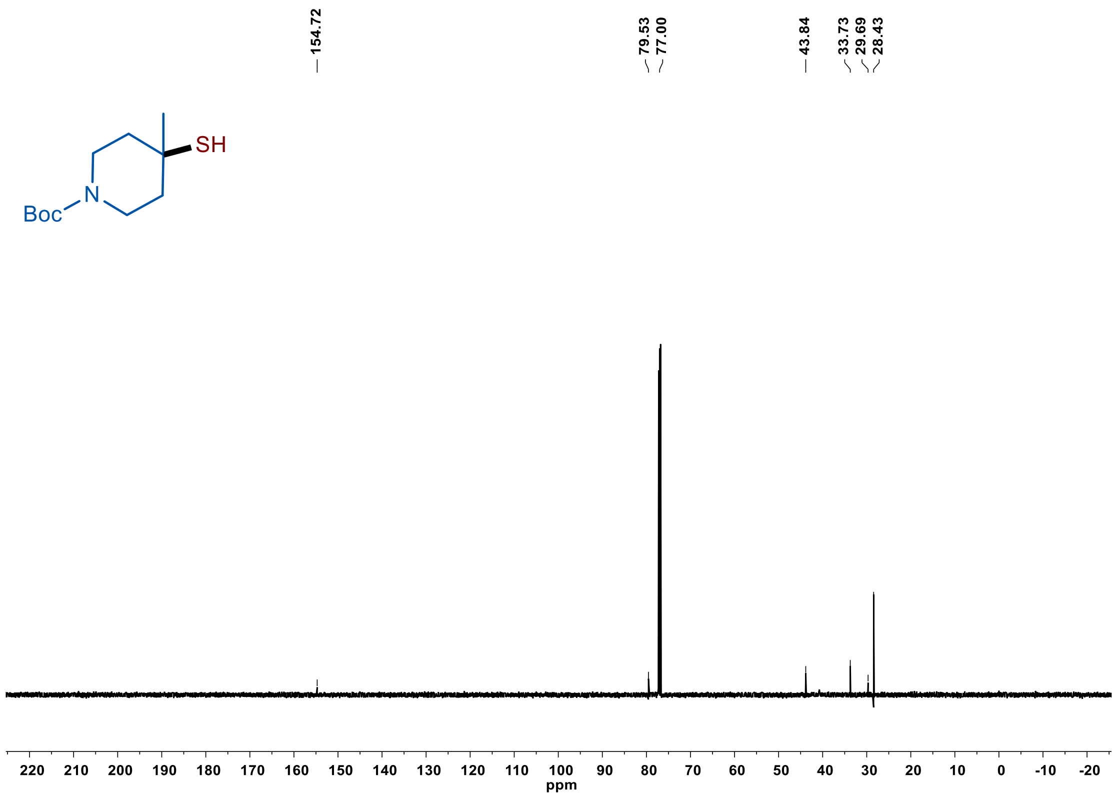

Supplementary Figure 40. ^{1}H and ^{13}C NMR spectra for compound **32**

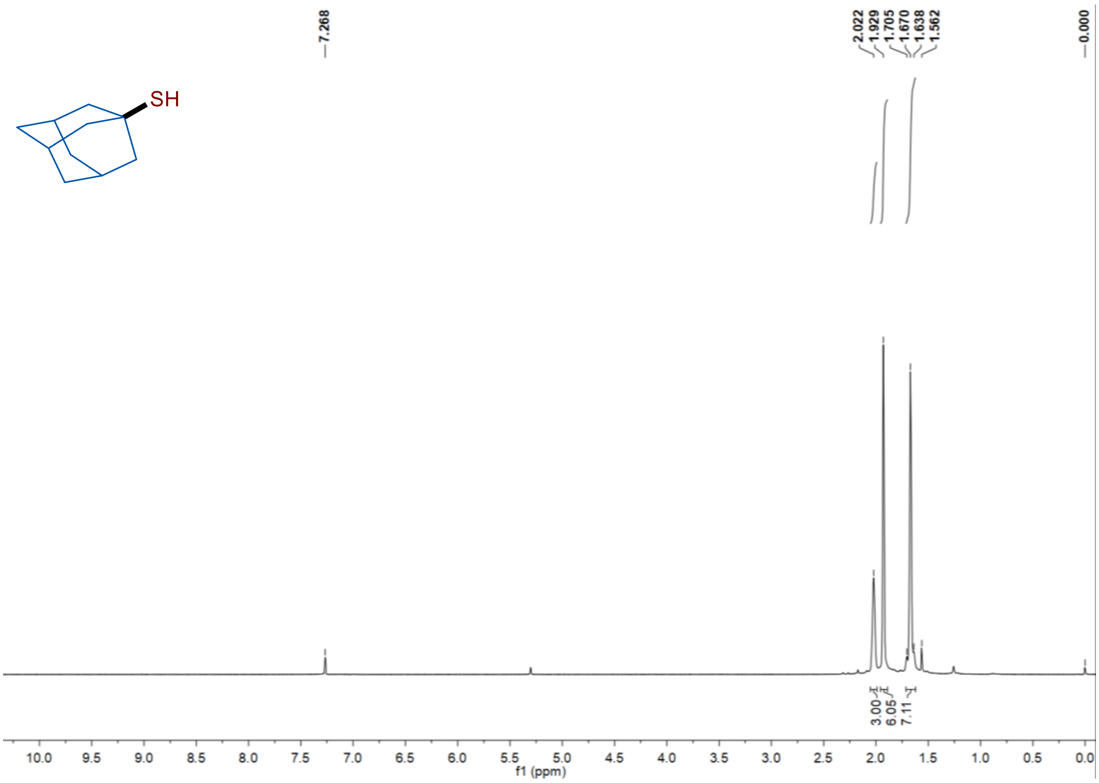

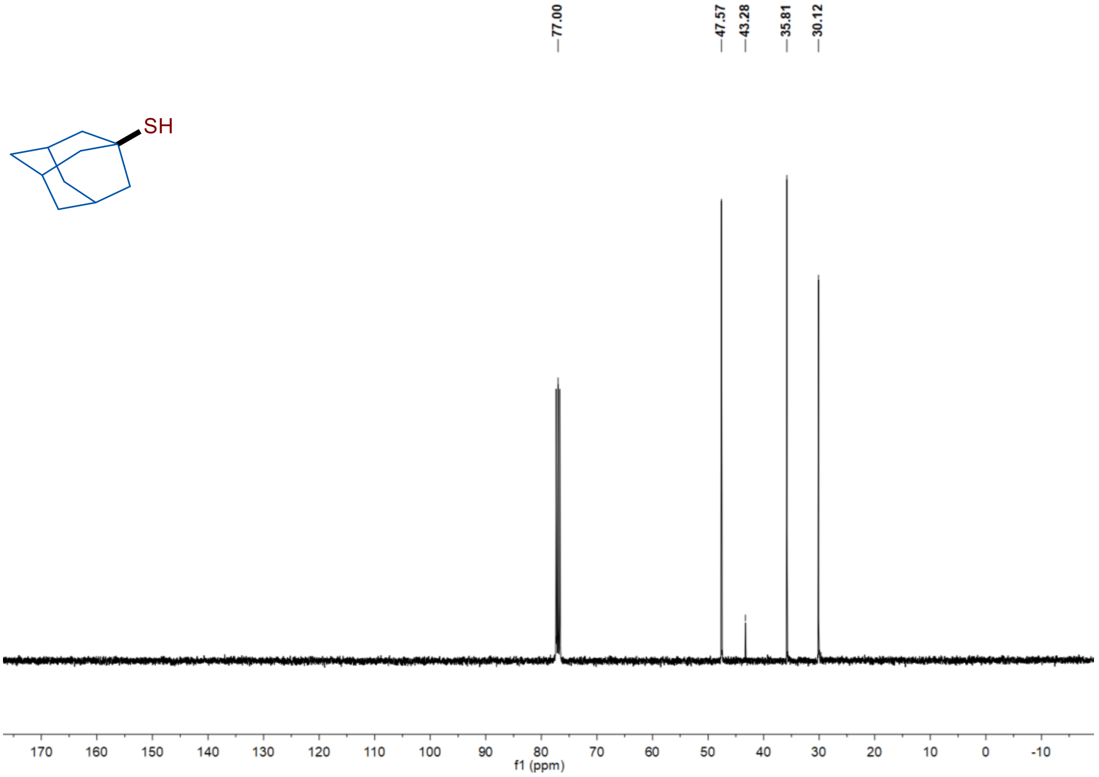

Supplementary Figure 41. ^{1}H and ^{13}C NMR spectra for compound **33**

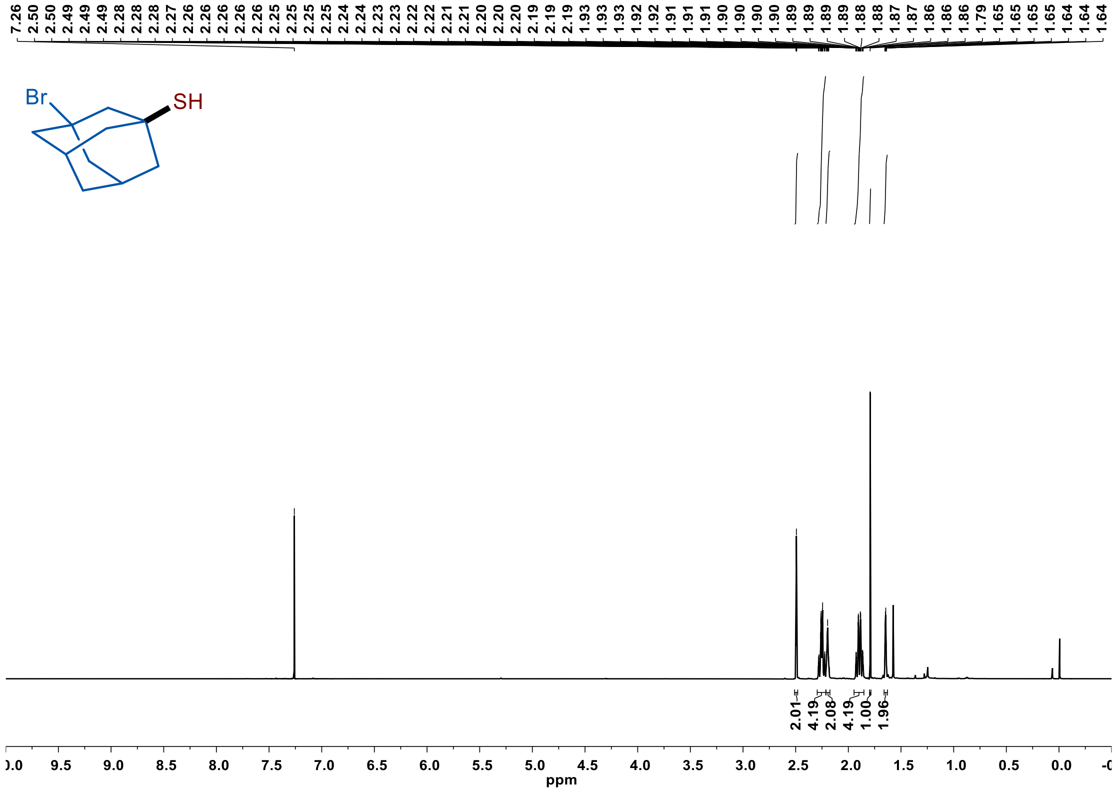

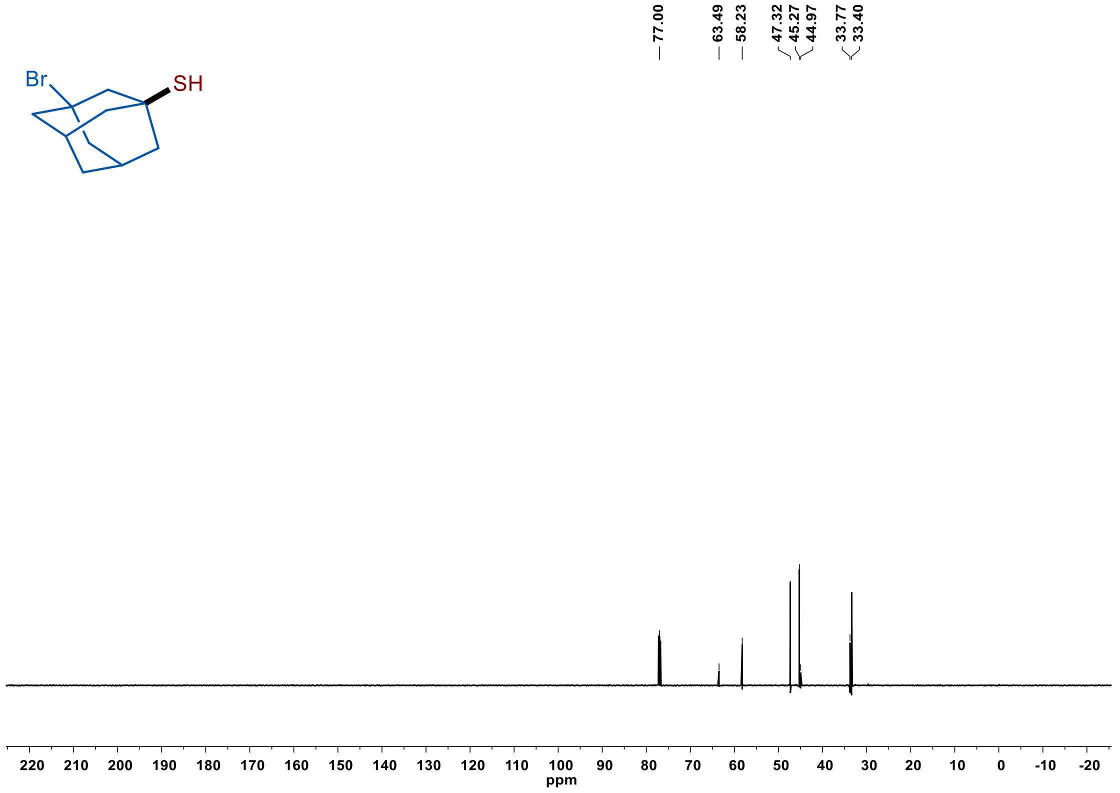

Supplementary Figure 42. ^{1}H and ^{13}C NMR spectra for compound **34**

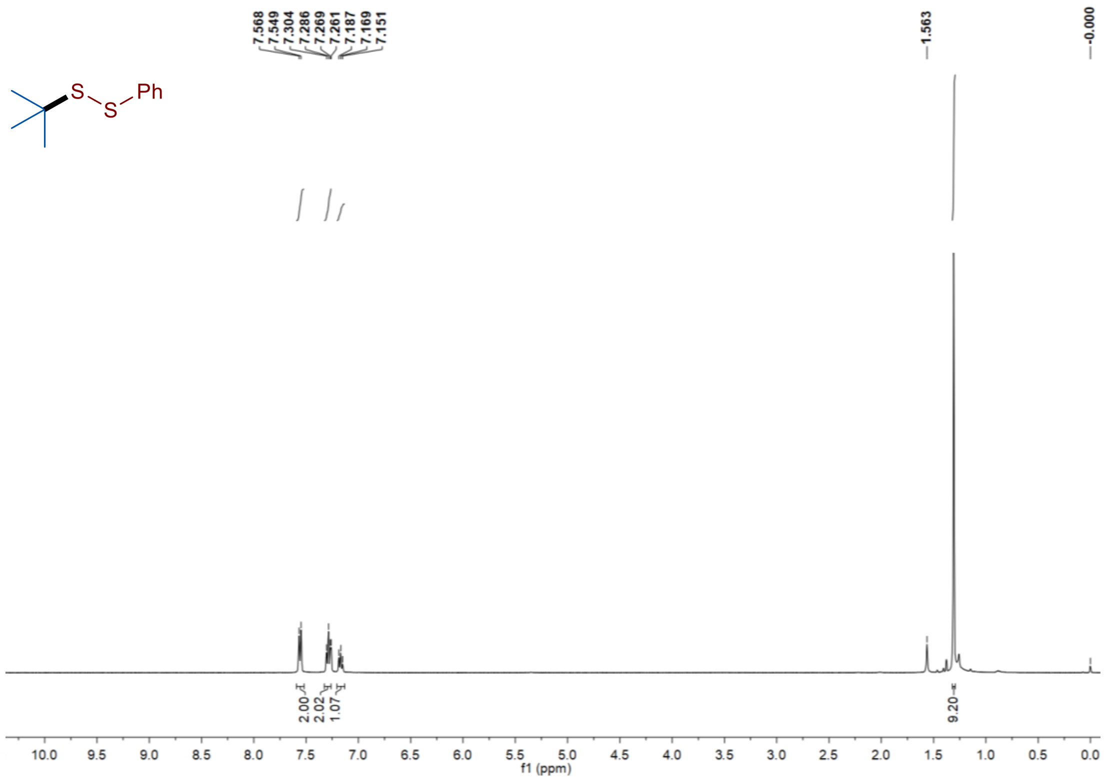

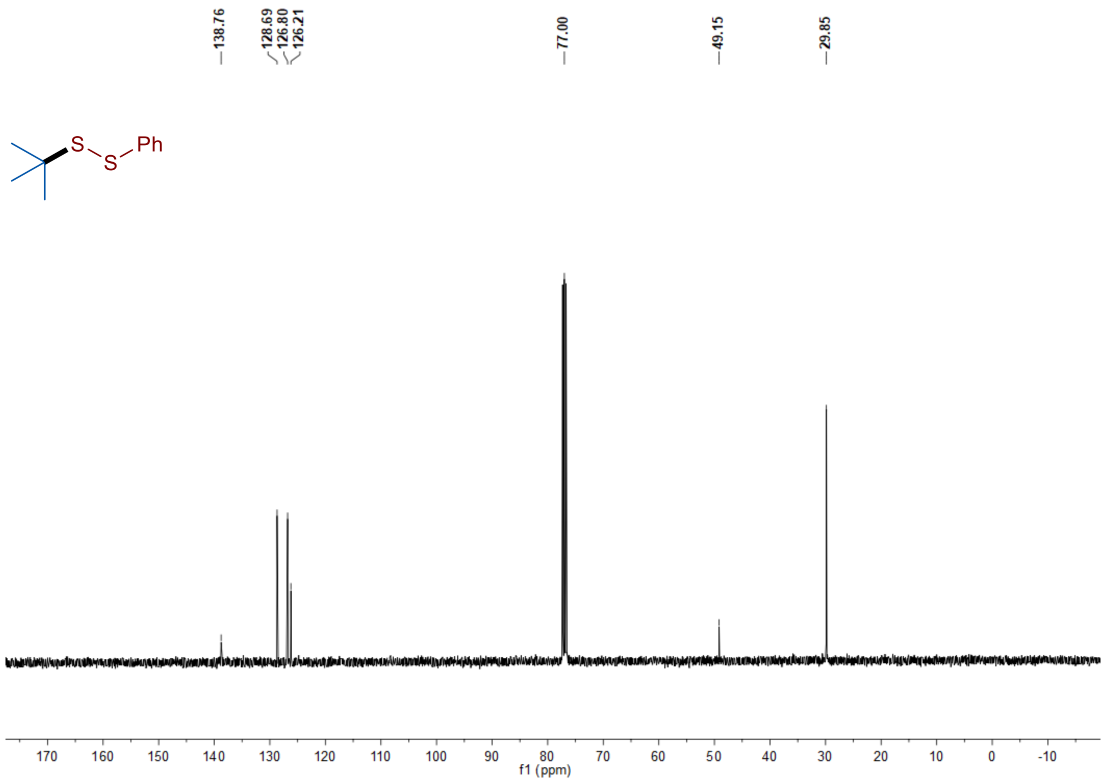

Supplementary Figure 43. ^{1}H and ^{13}C NMR spectra for compound **35**

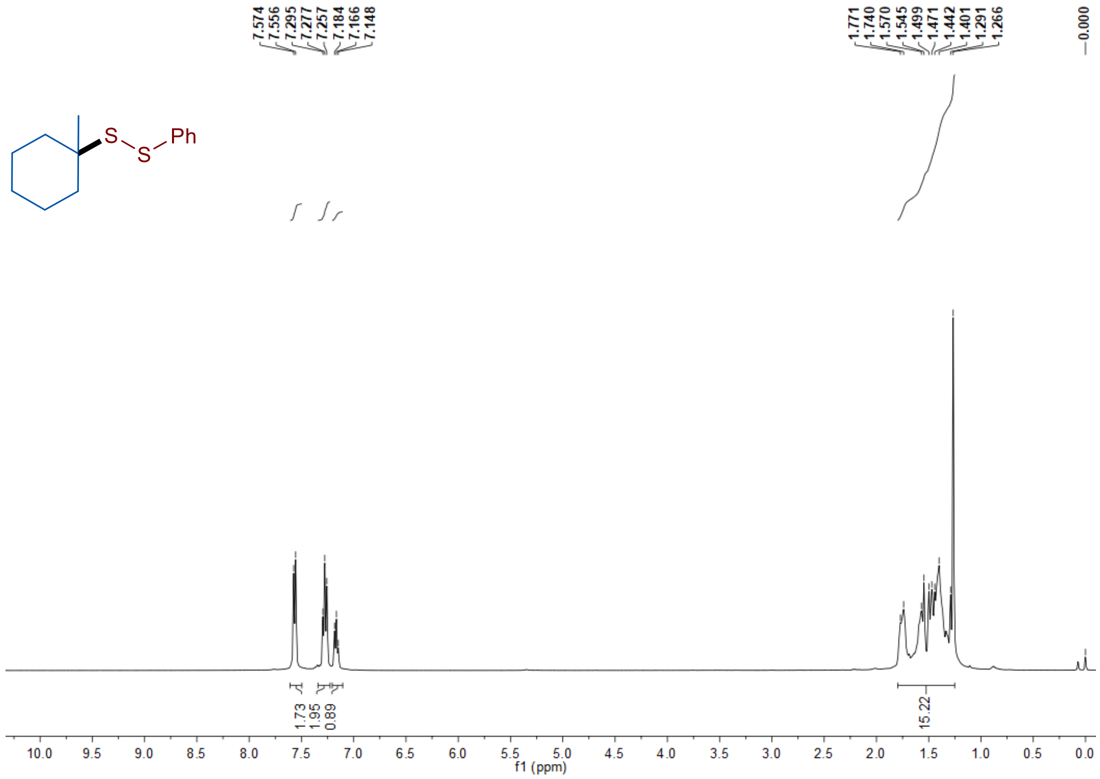

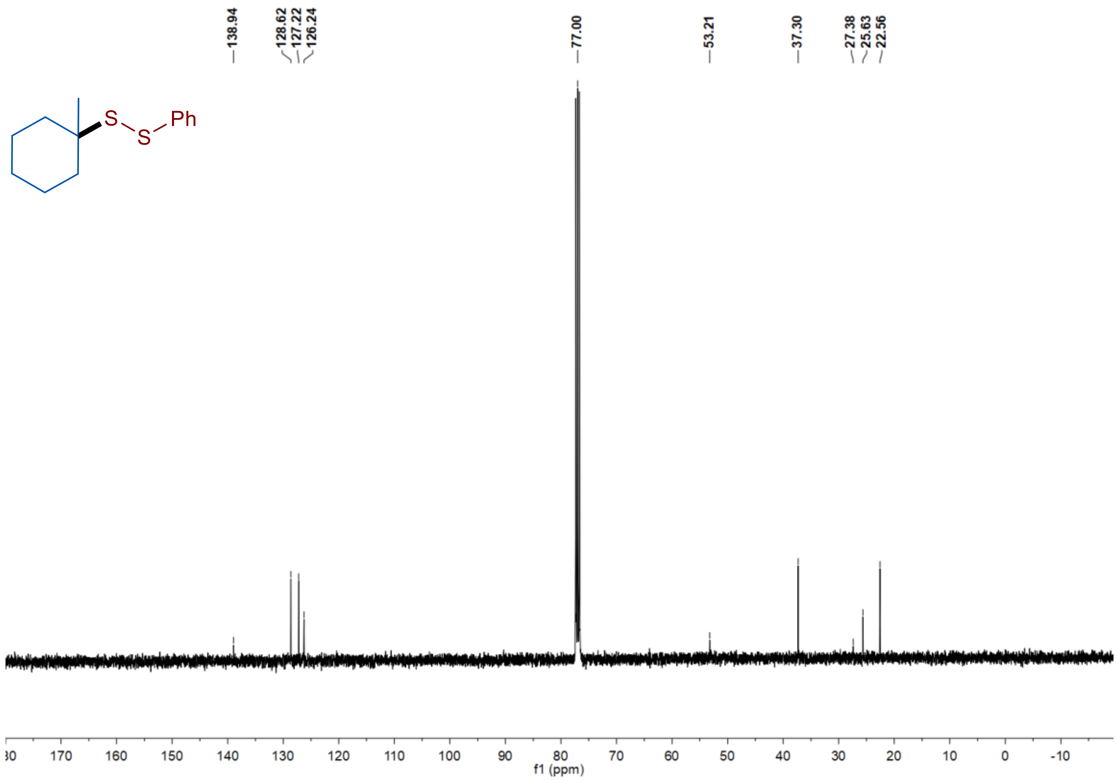

Supplementary Figure 44. ^{1}H and ^{13}C NMR spectra for compound **36**

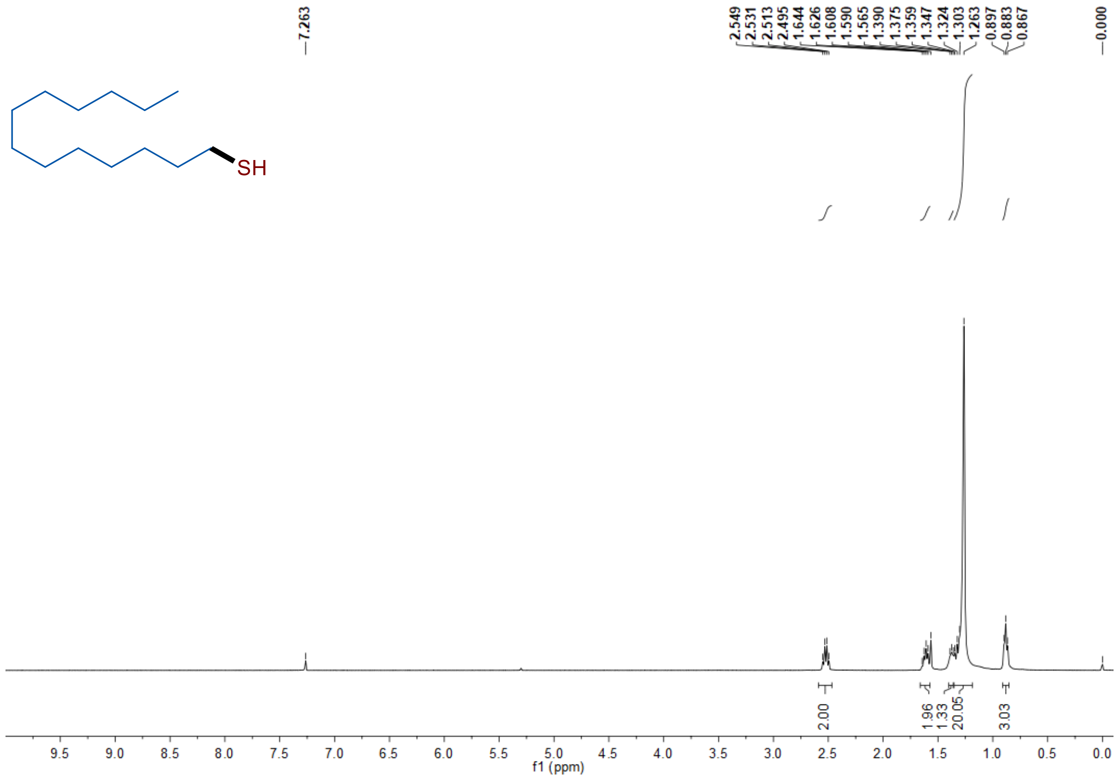

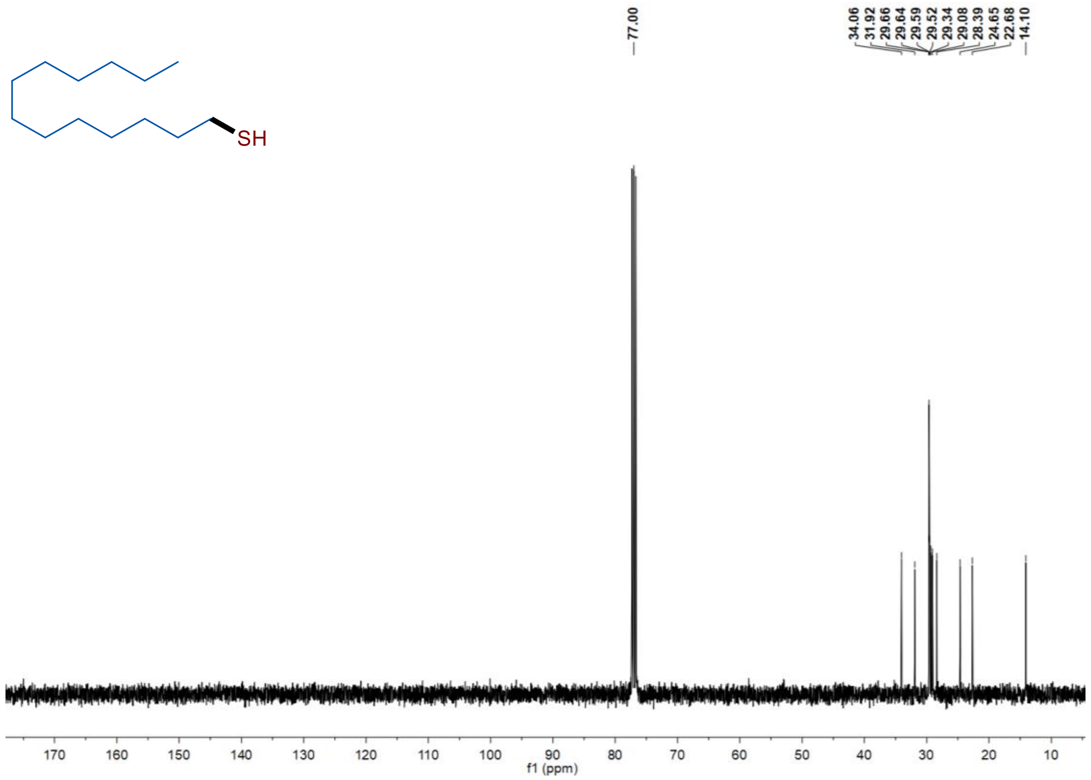

Supplementary Figure 45. ^{1}H and ^{13}C NMR spectra for compound **37**

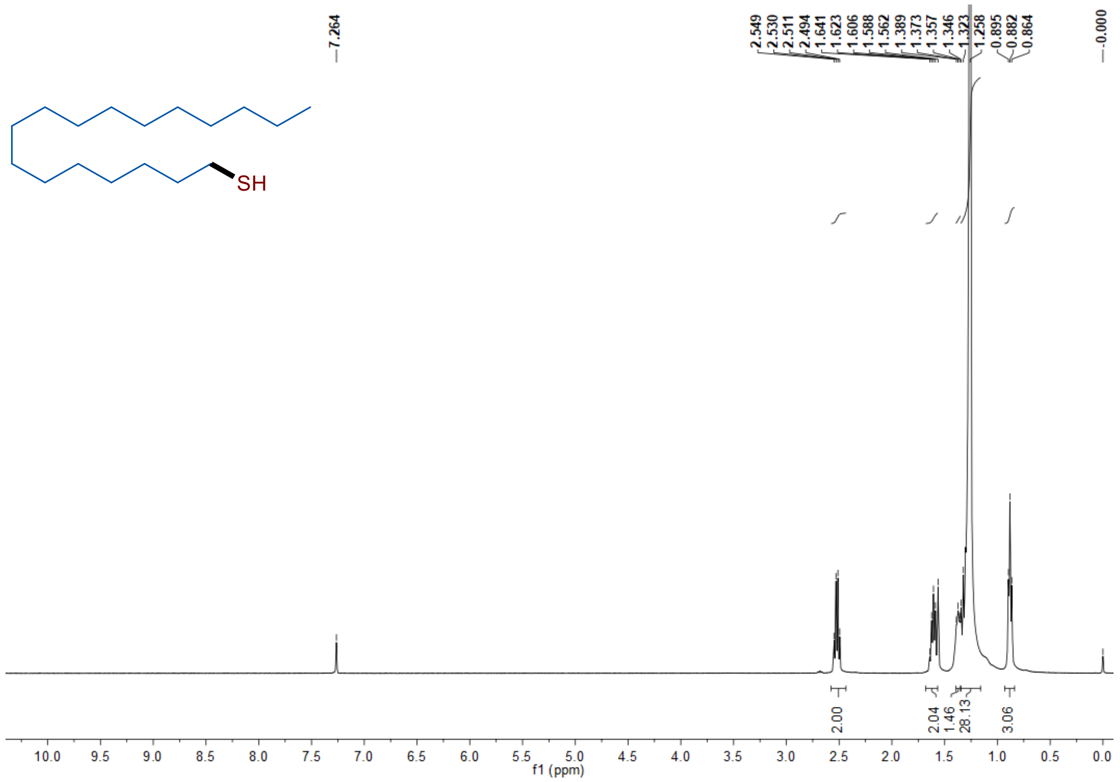

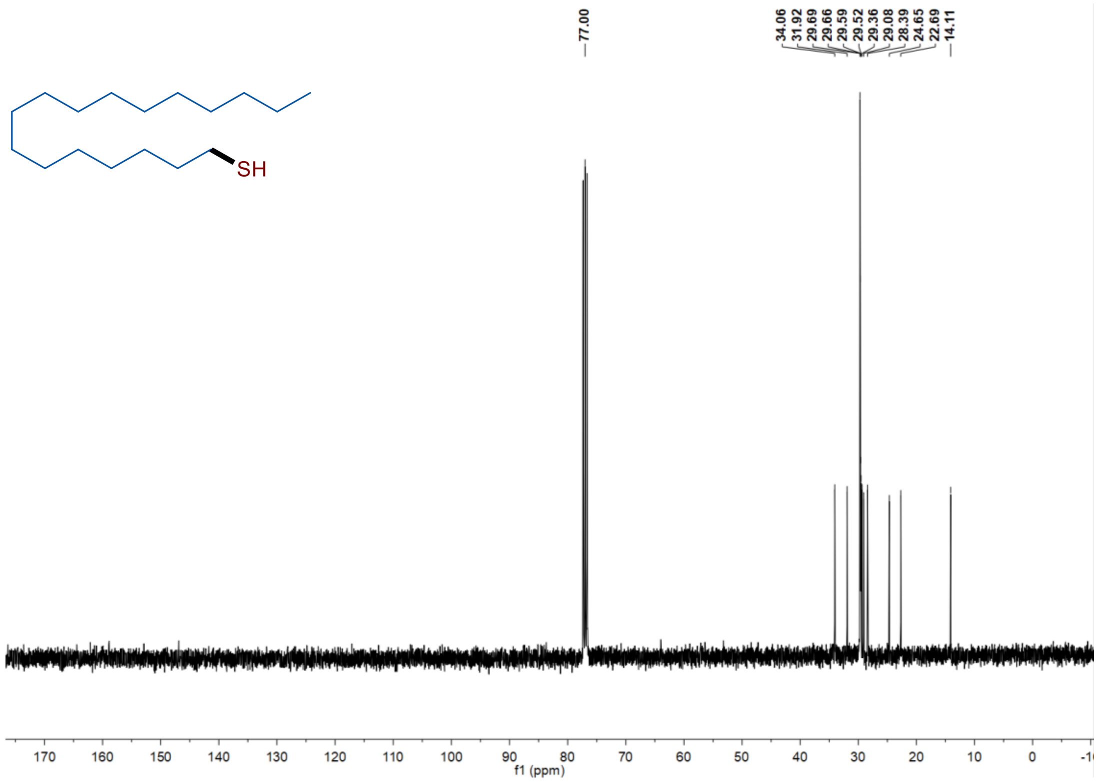

Supplementary Figure 46. ^{1}H and ^{13}C NMR spectra for compound **38**

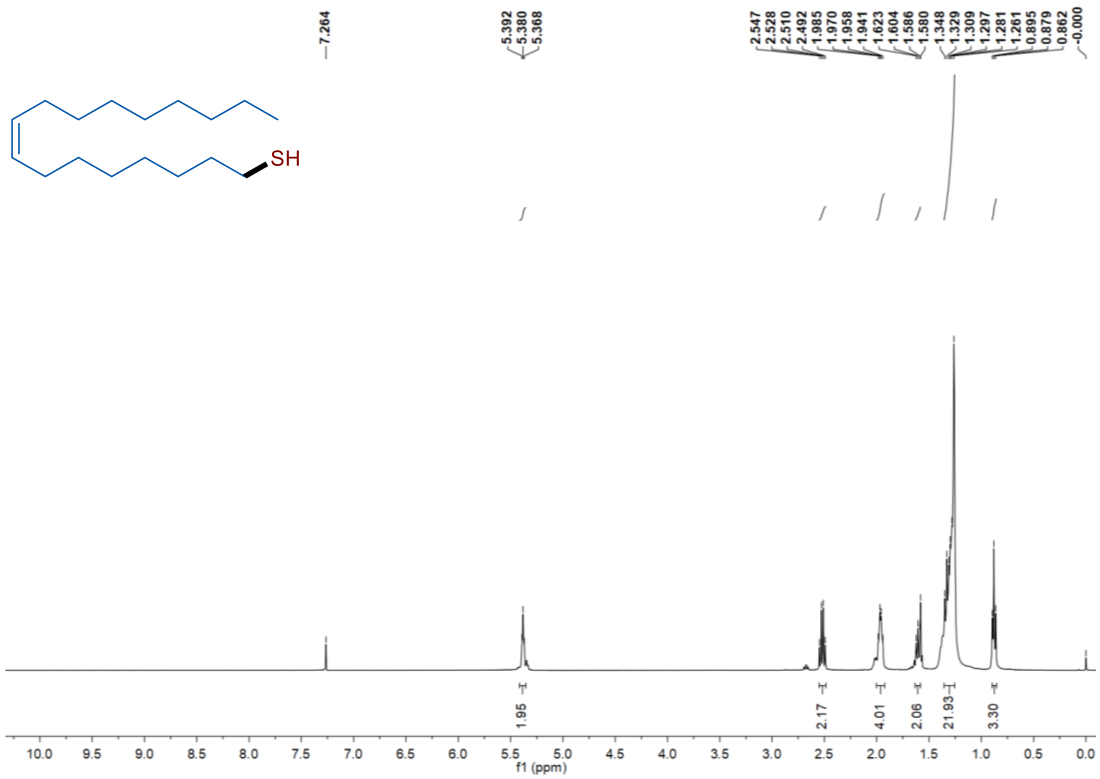

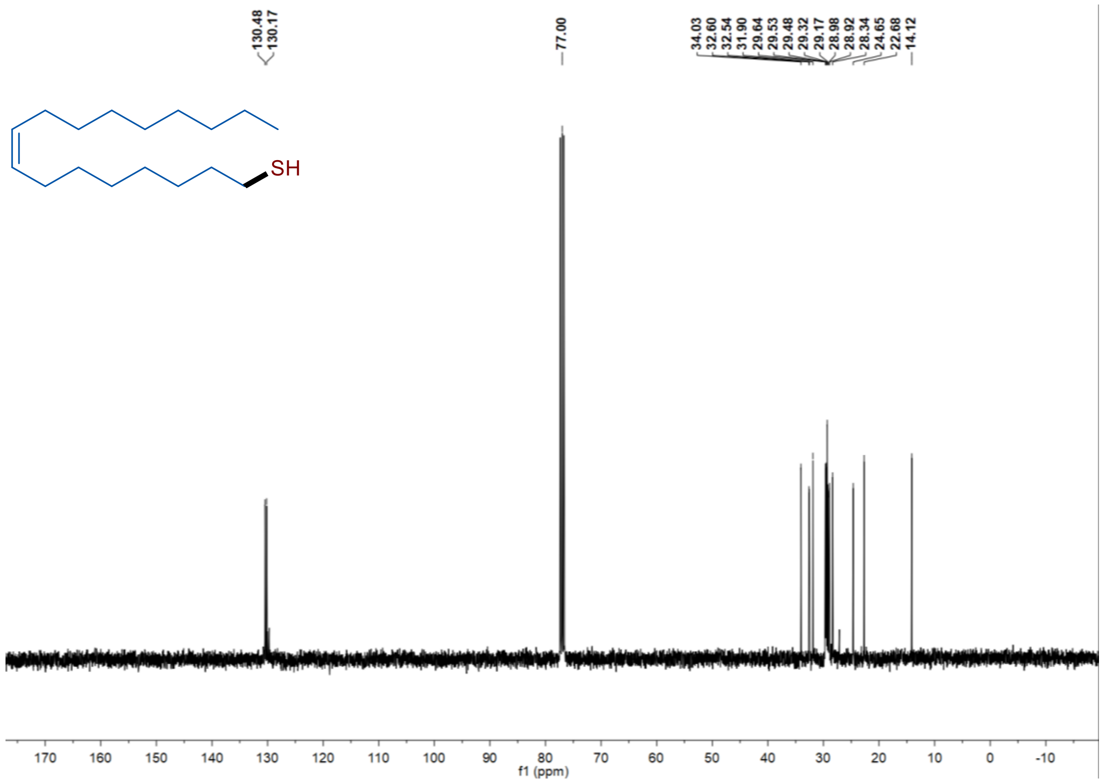

Supplementary Figure 47. ^{1}H and ^{13}C NMR spectra for compound **39**

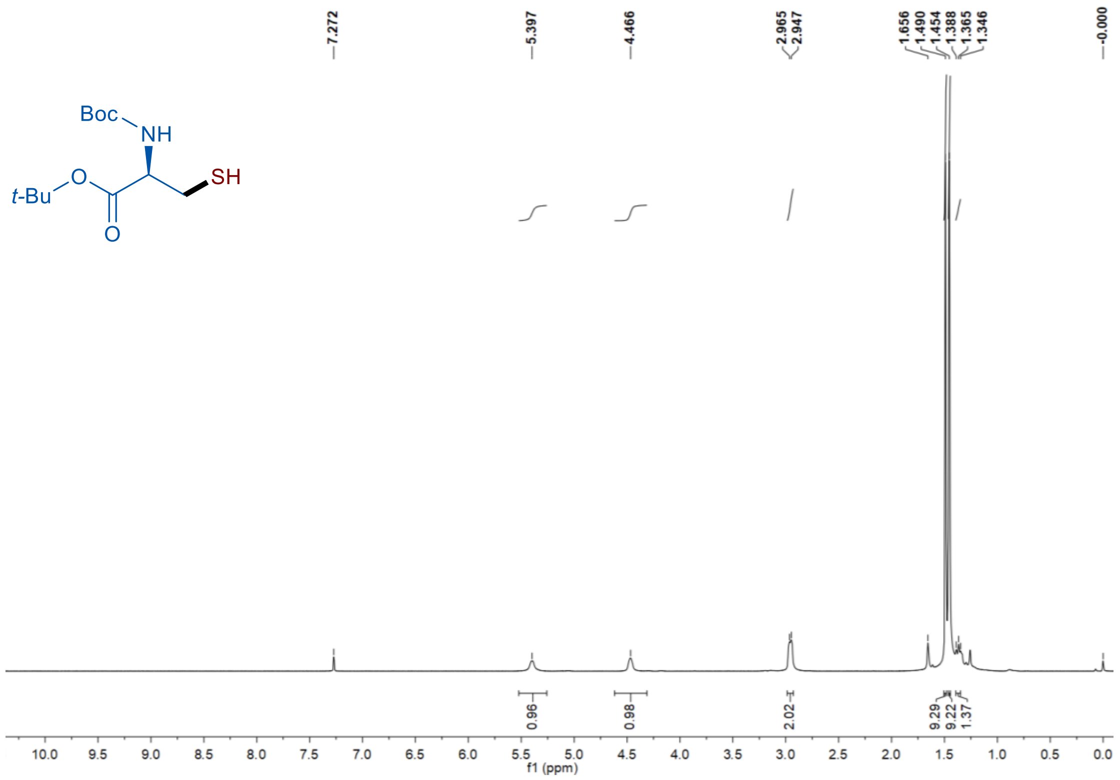

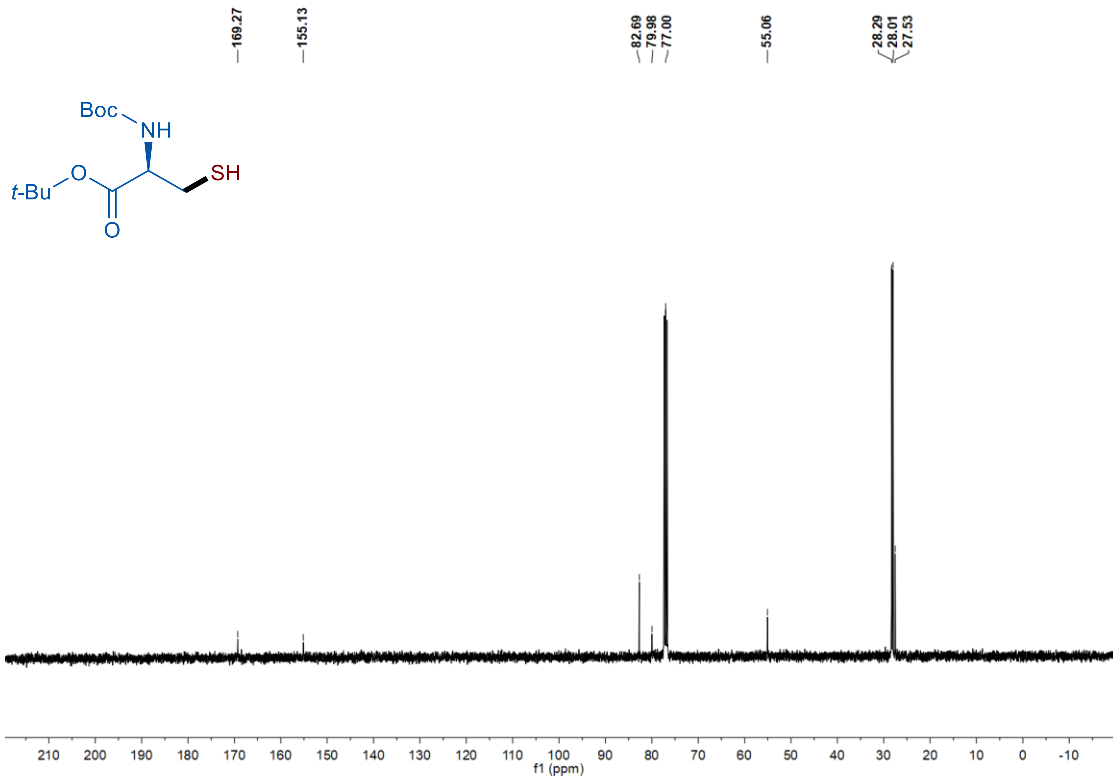

Supplementary Figure 48. ^{1}H and ^{13}C NMR spectra for compound 40

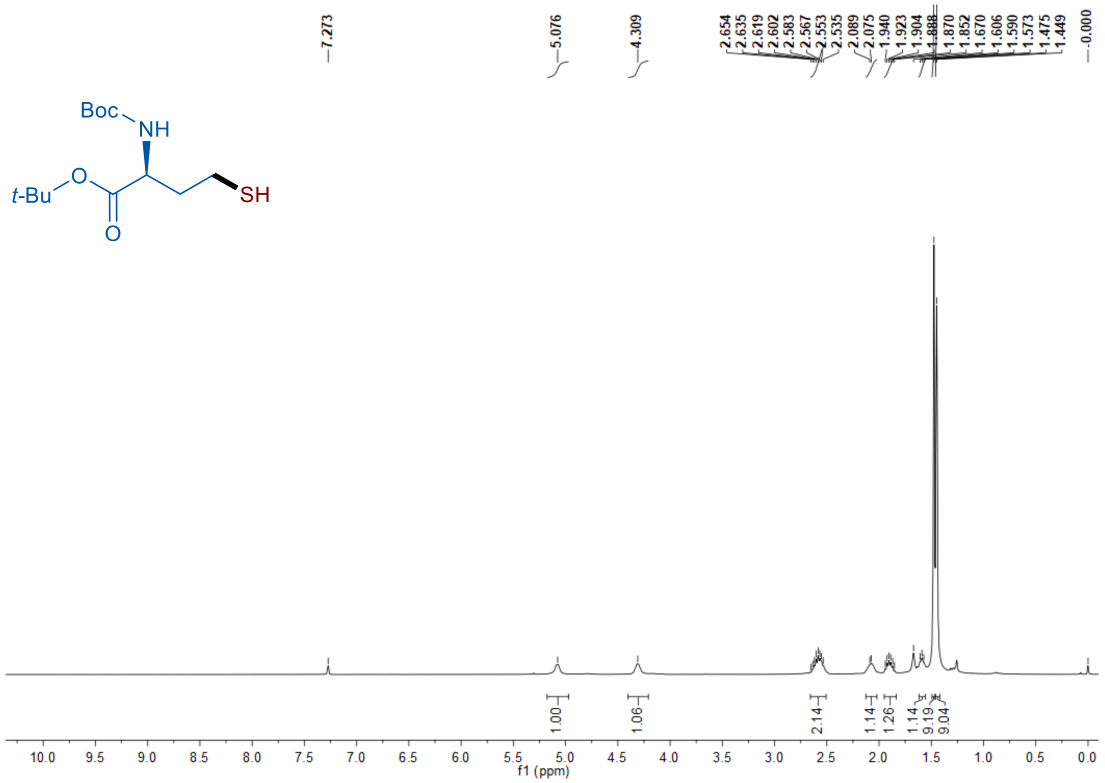

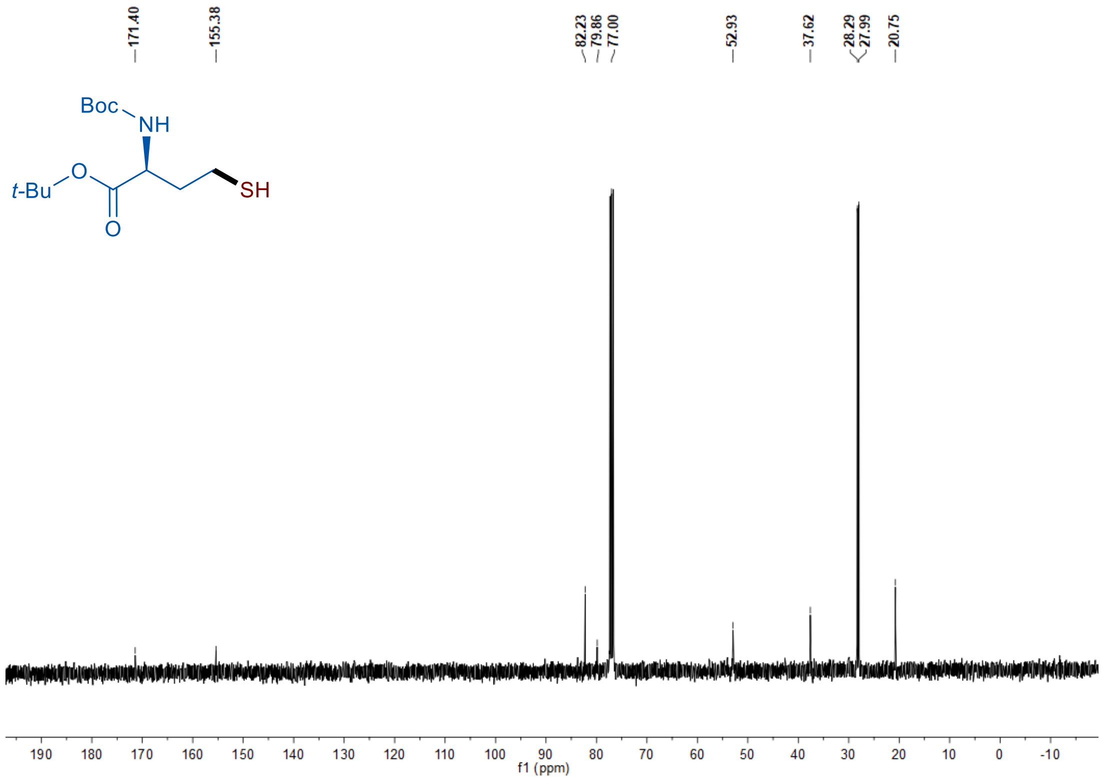

Supplementary Figure 49. ^{1}H and ^{13}C NMR spectra for compound 41

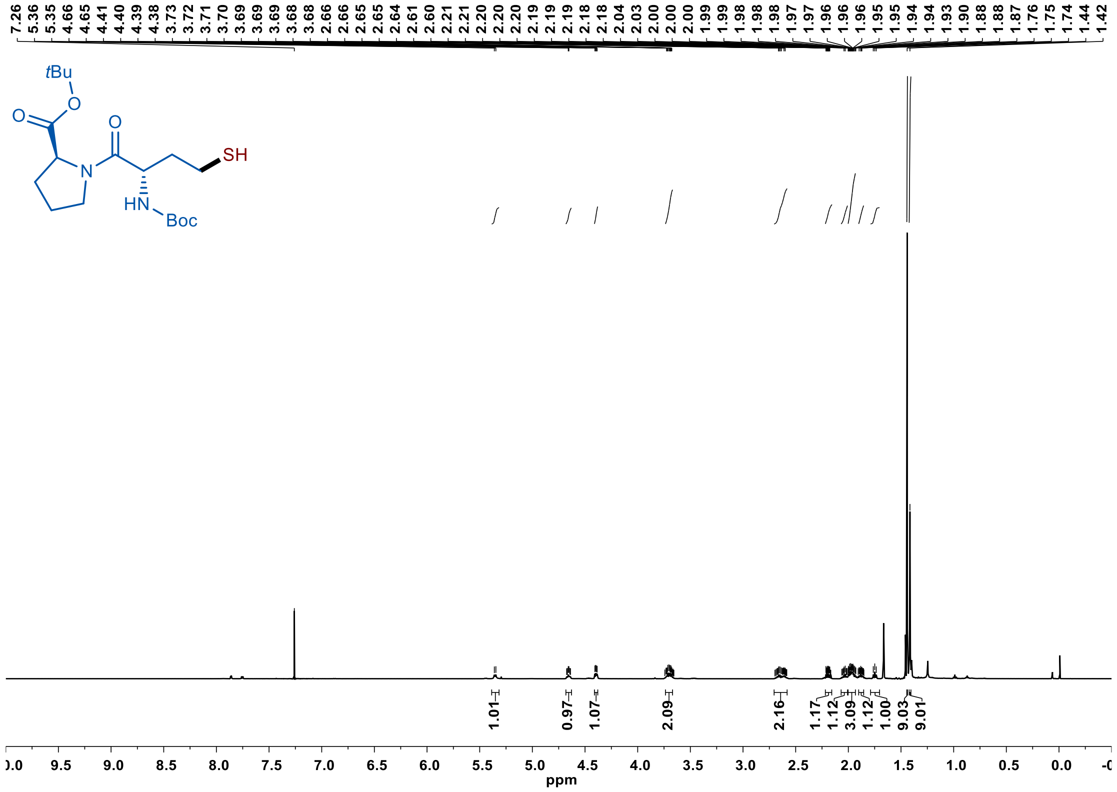

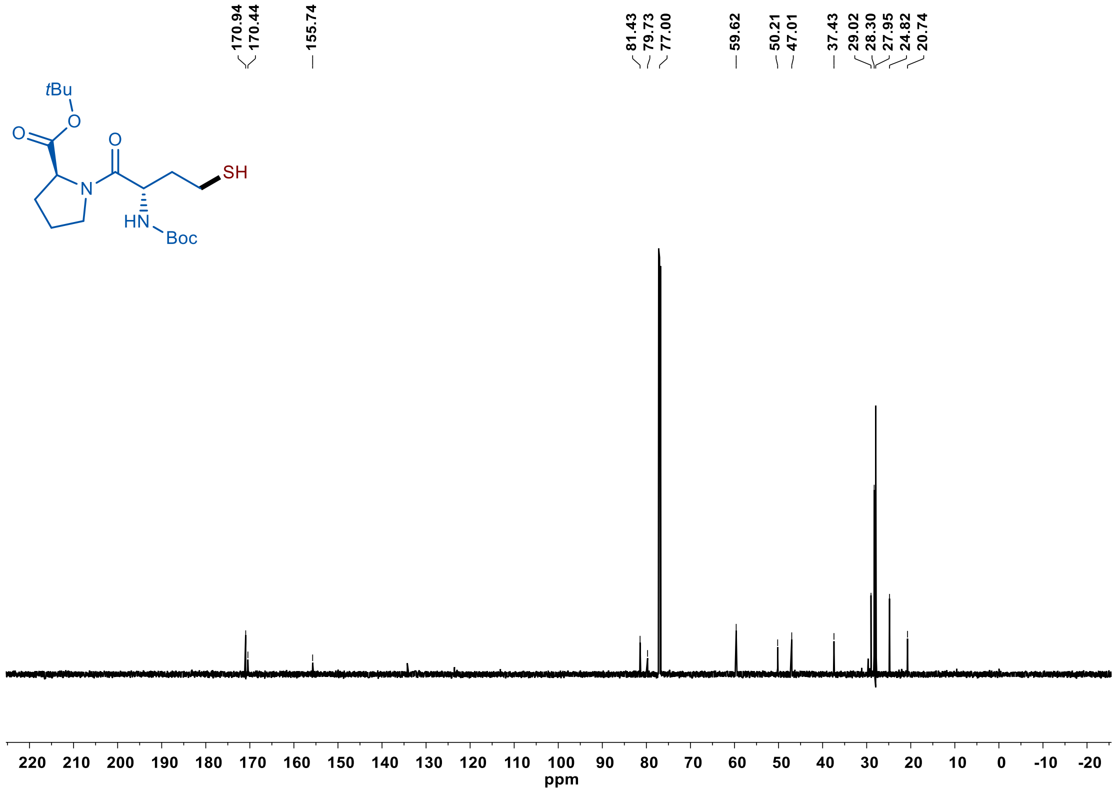

Supplementary Figure 50. ^{1}H and ^{13}C NMR spectra for compound **42**

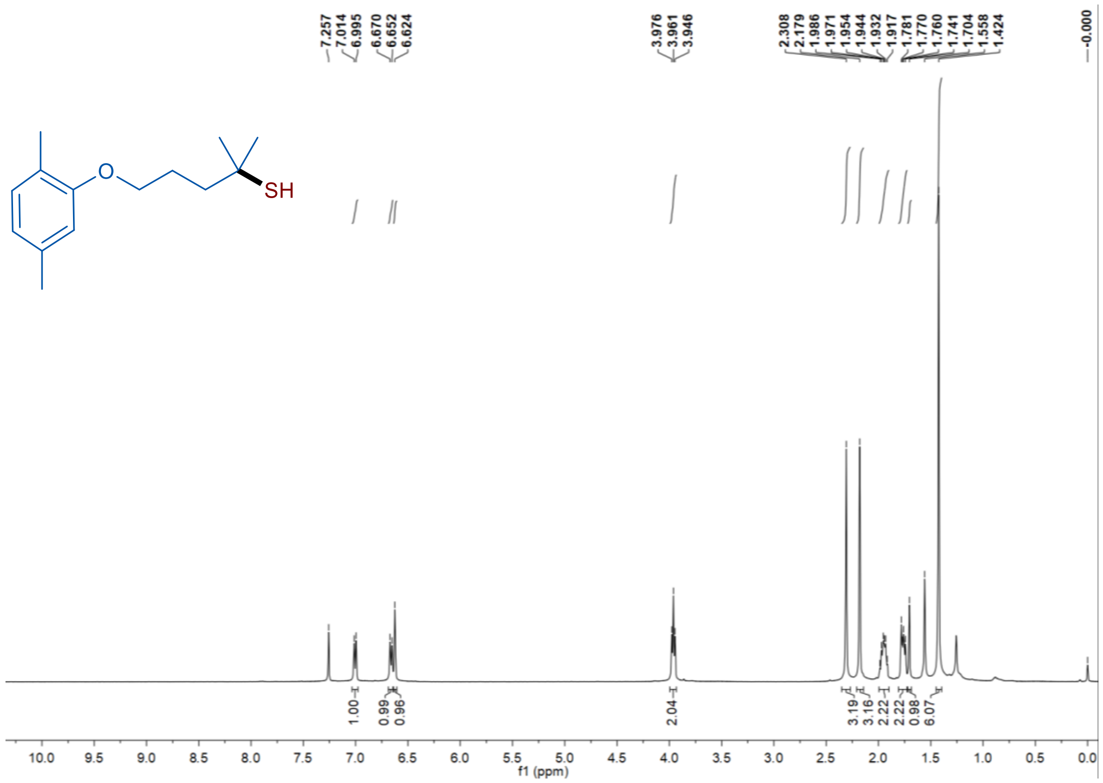

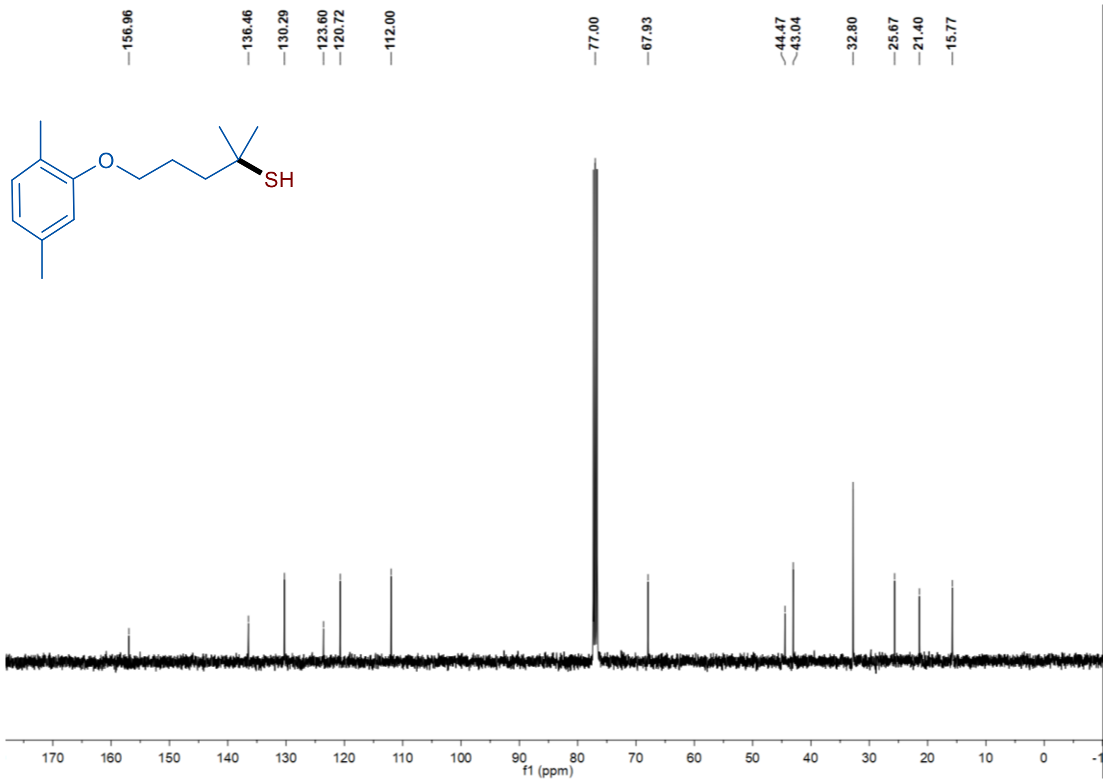

Supplementary Figure 51. ^{1}H and ^{13}C NMR spectra for compound **43**

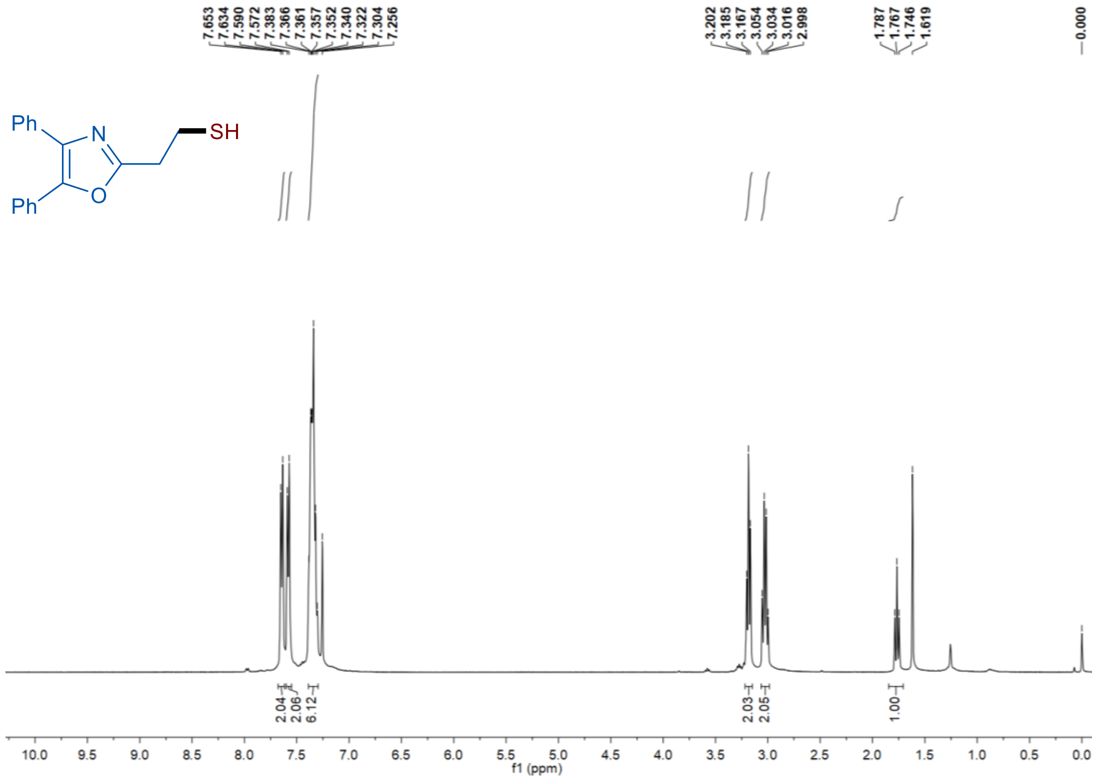

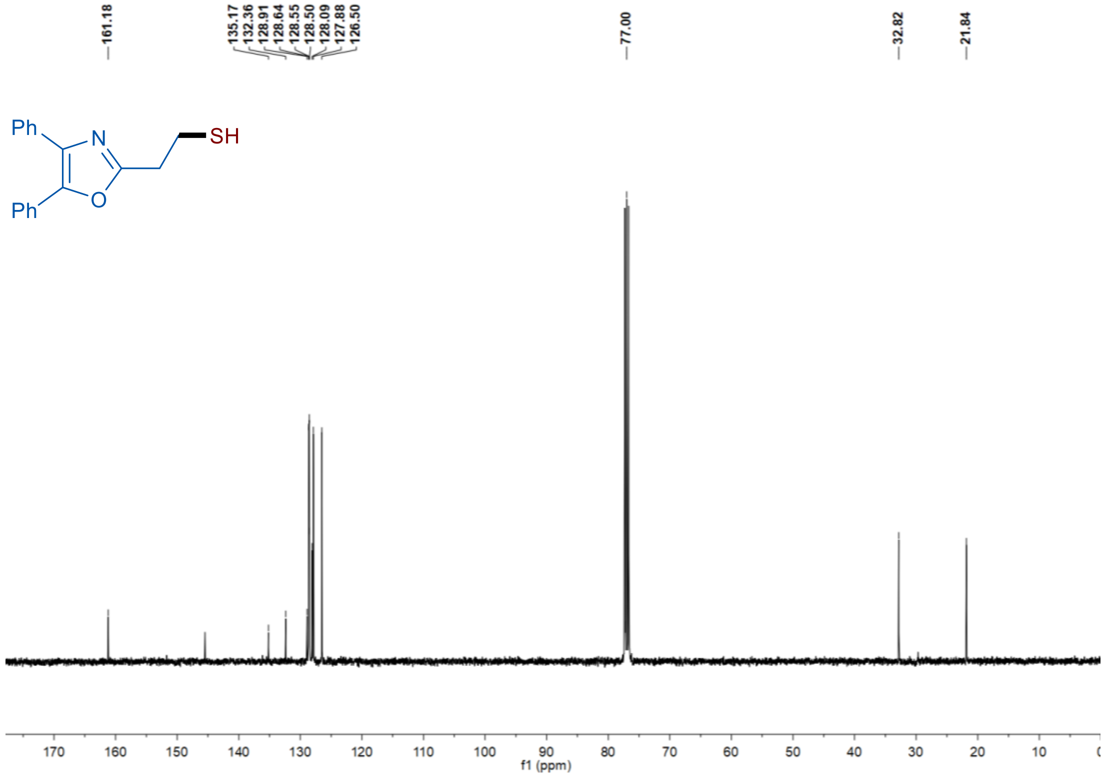

Supplementary Figure 52. ^{1}H and ^{13}C NMR spectra for compound **44**

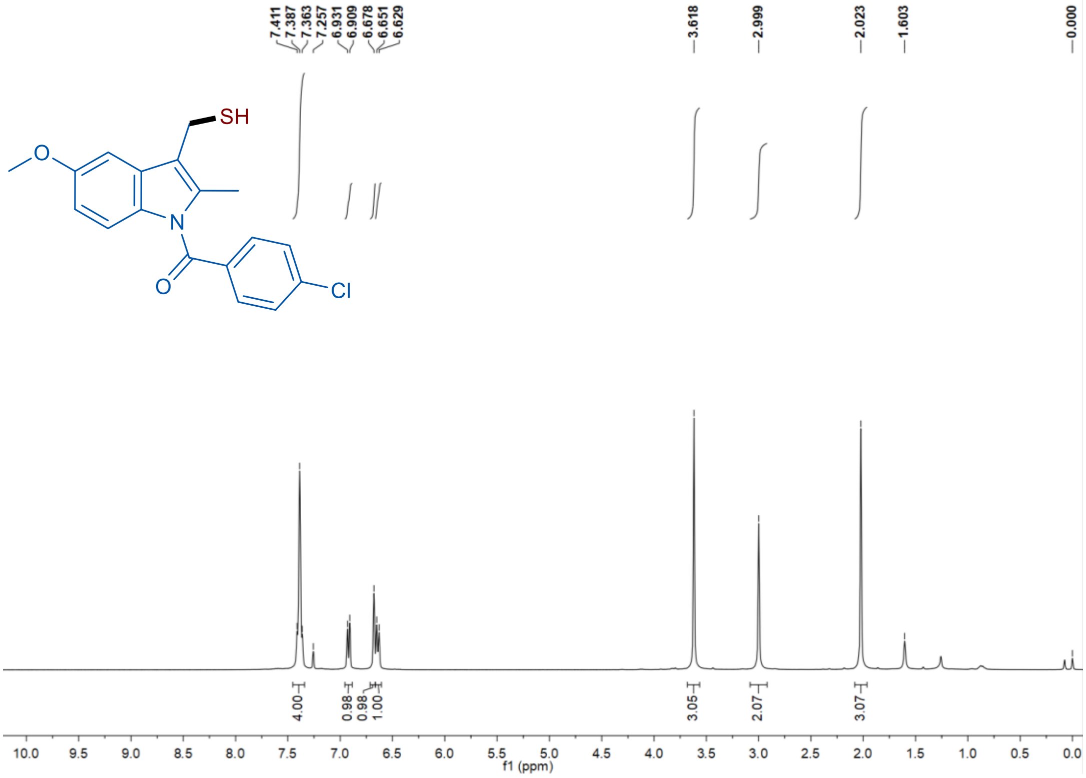

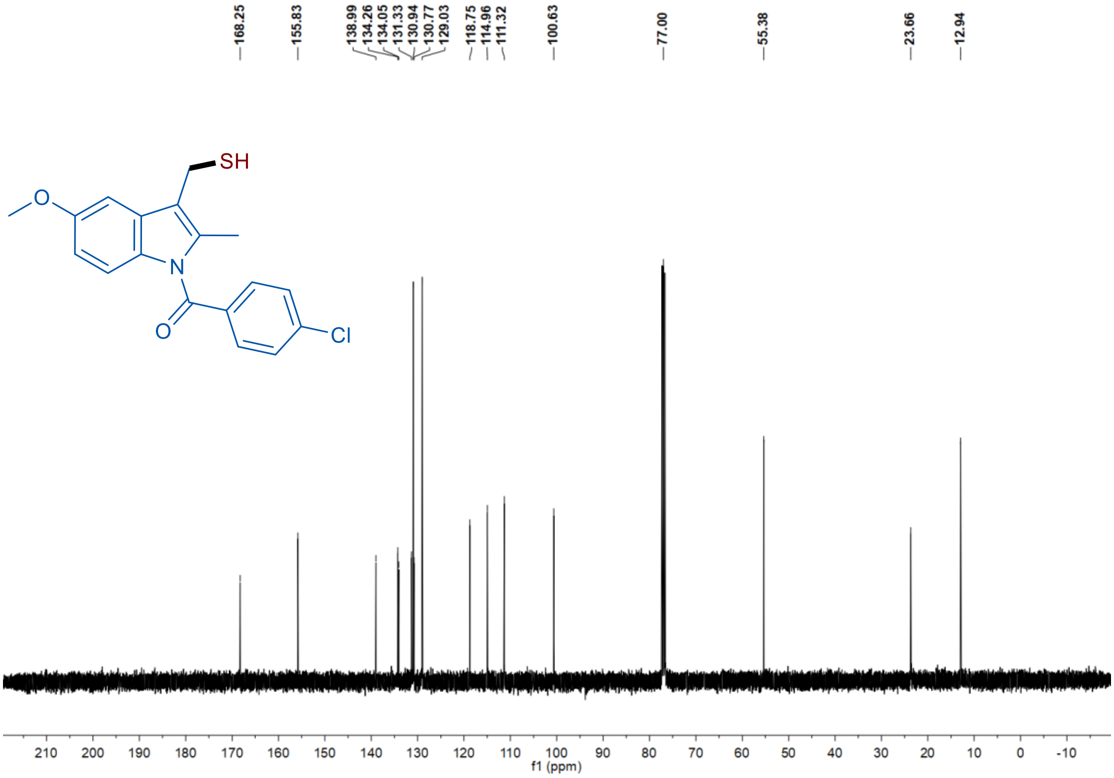

Supplementary Figure 53. ^{1}H and ^{13}C NMR spectra for compound **45**

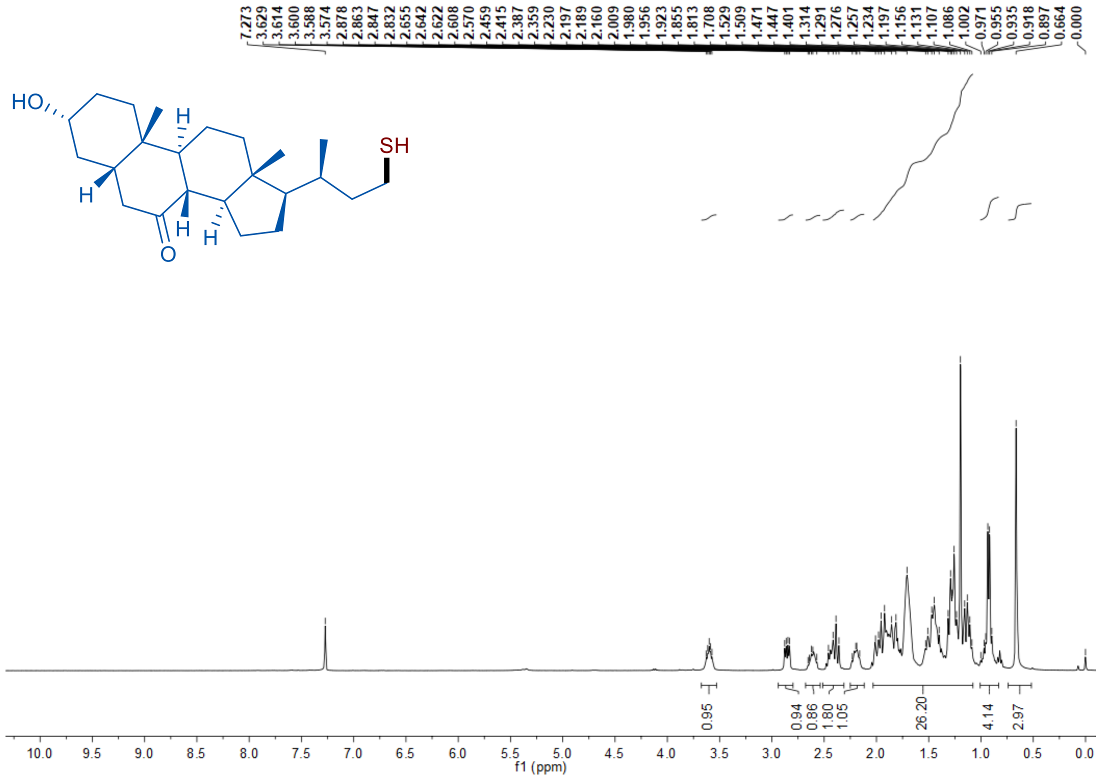

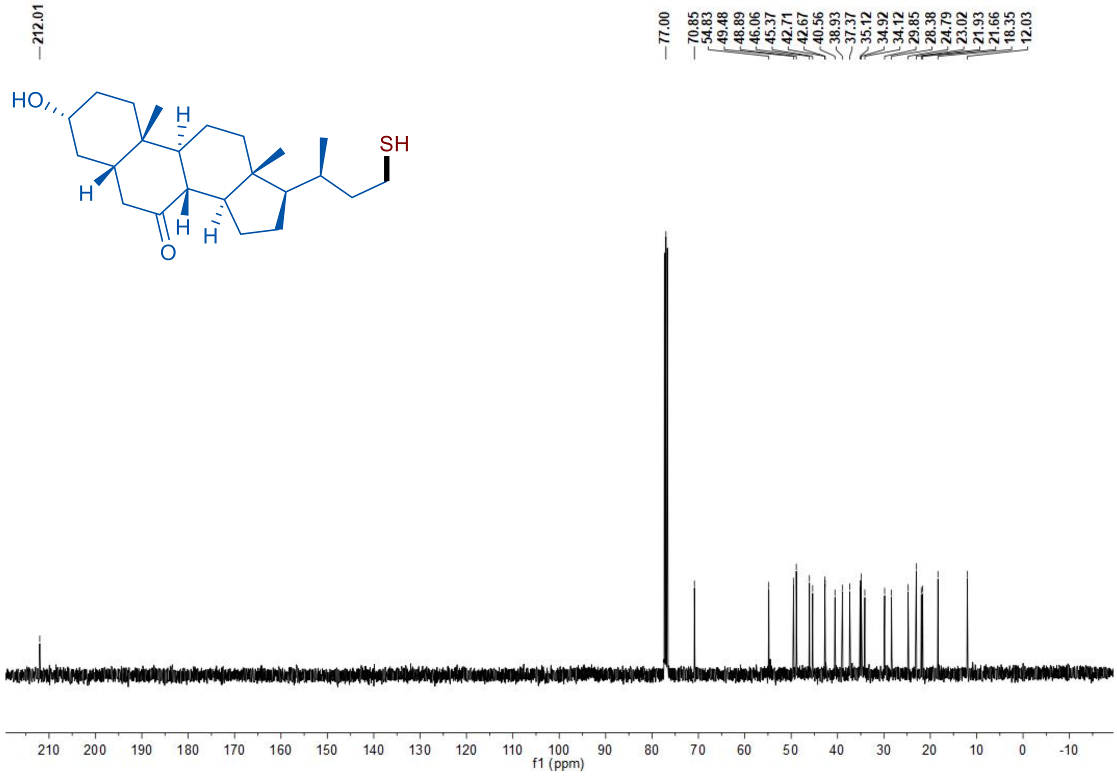

Supplementary Figure 54. ^{1}H and ^{13}C NMR spectra for compound **46**

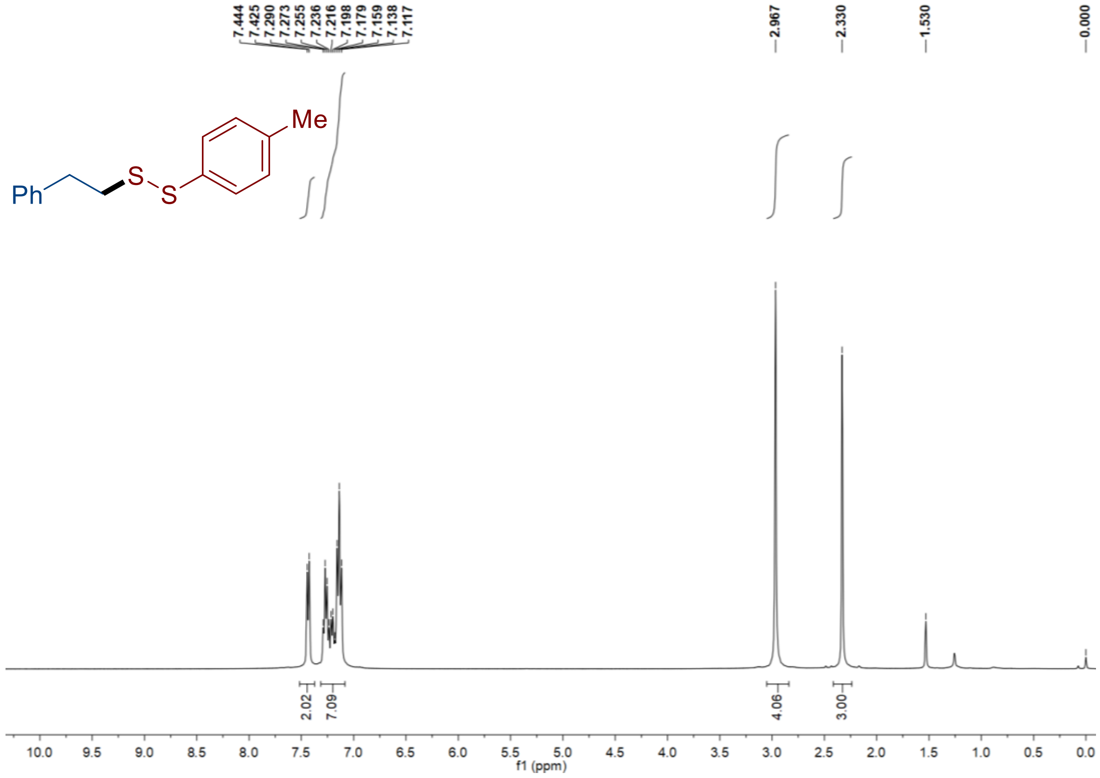

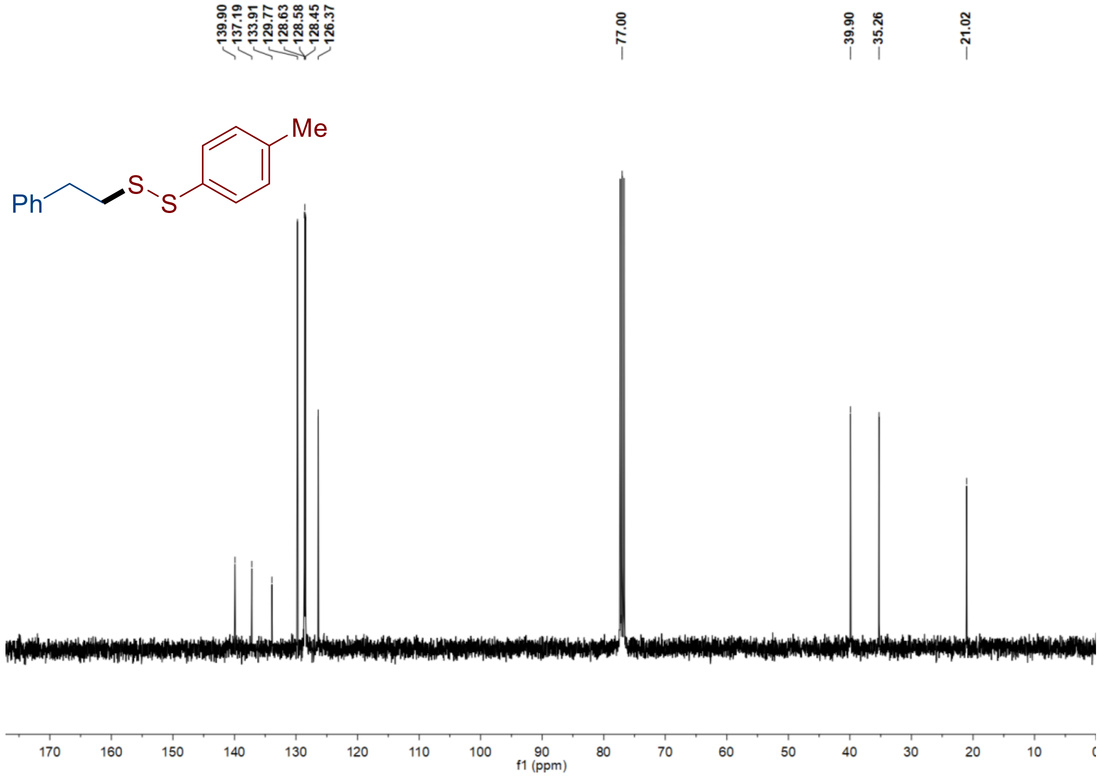

Supplementary Figure 55. ^{1}H and ^{13}C NMR spectra for compound **47**

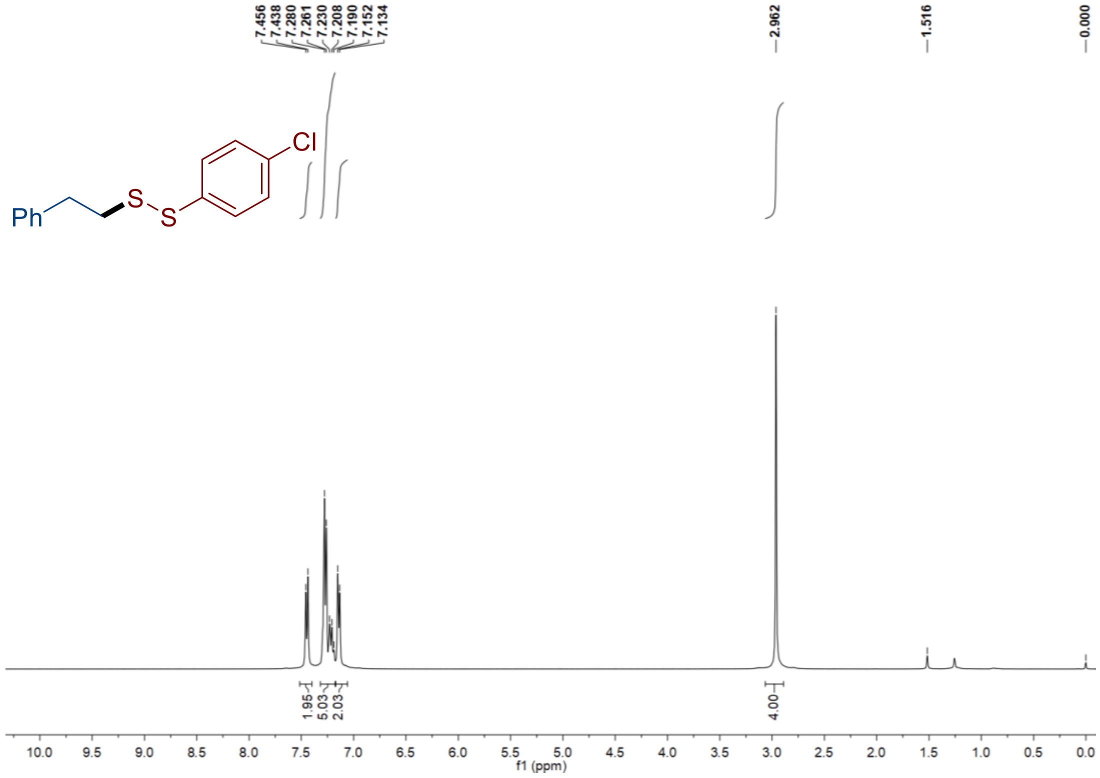

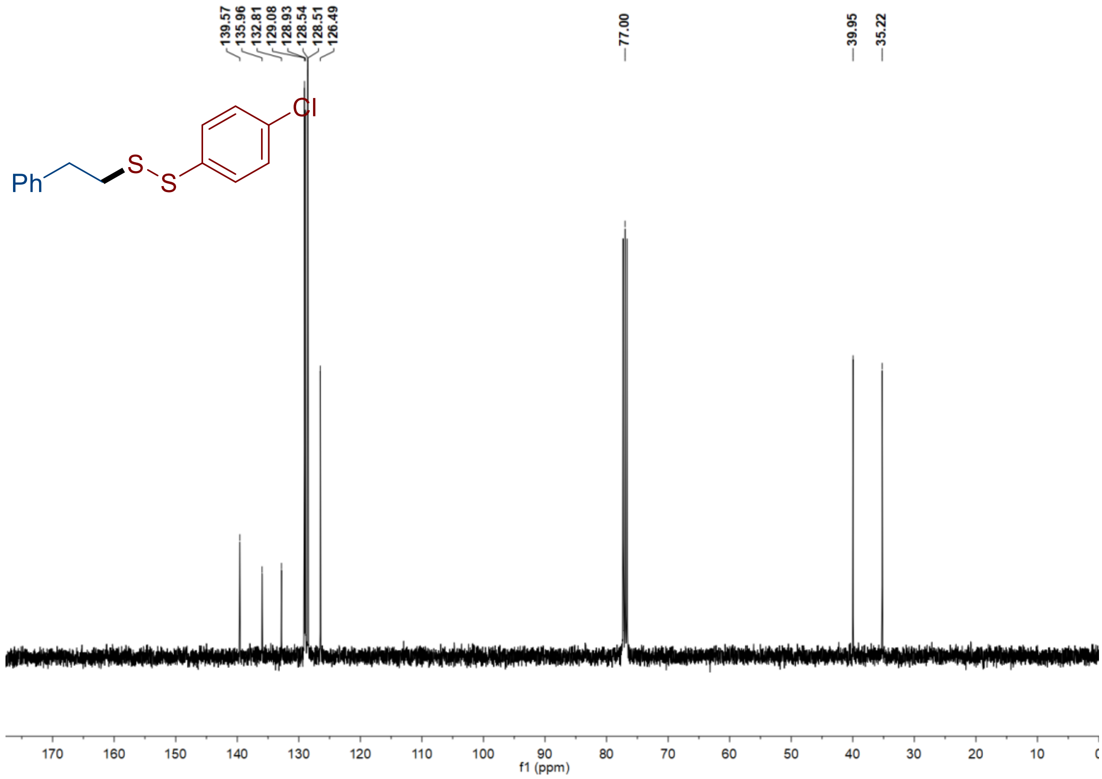

Supplementary Figure 56. ^{1}H and ^{13}C NMR spectra for compound **48**

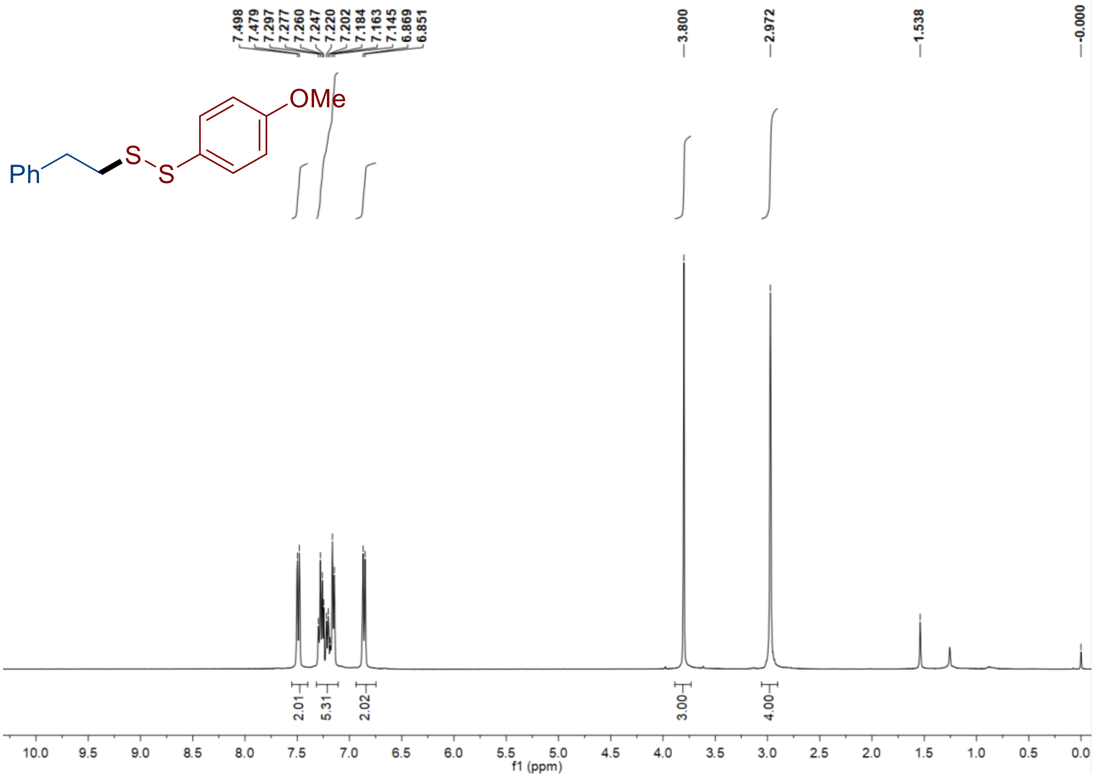

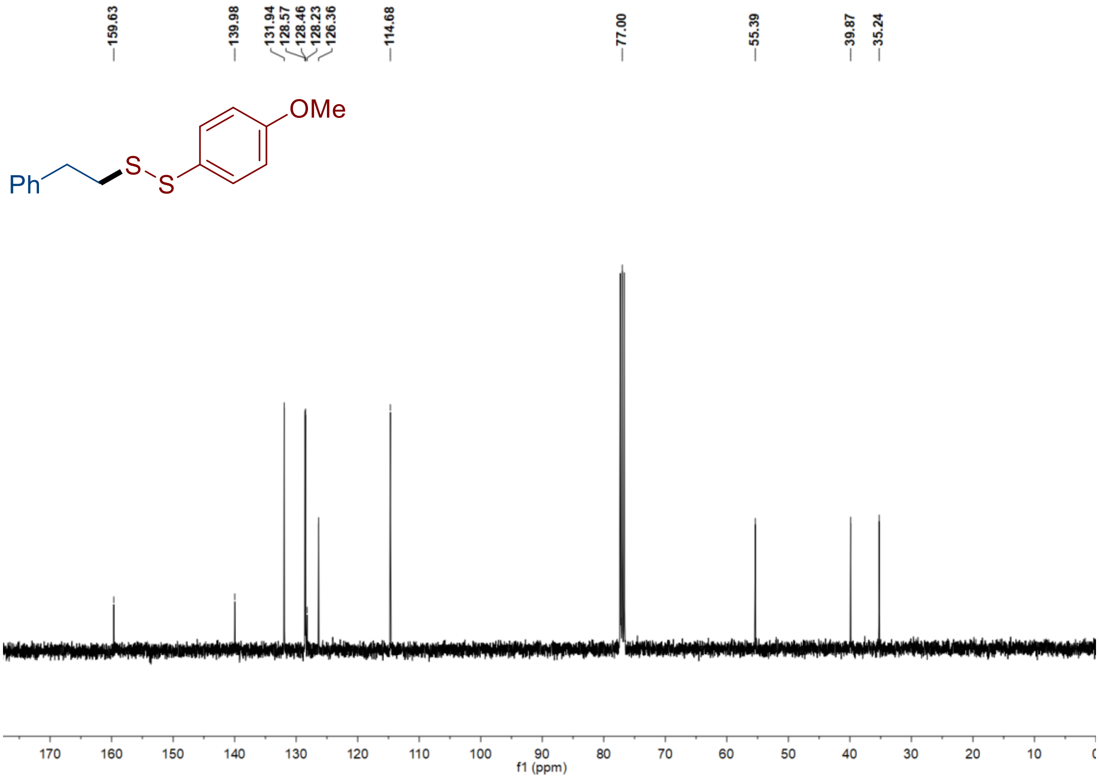

Supplementary Figure 57. ^{1}H and ^{13}C NMR spectra for compound **49**

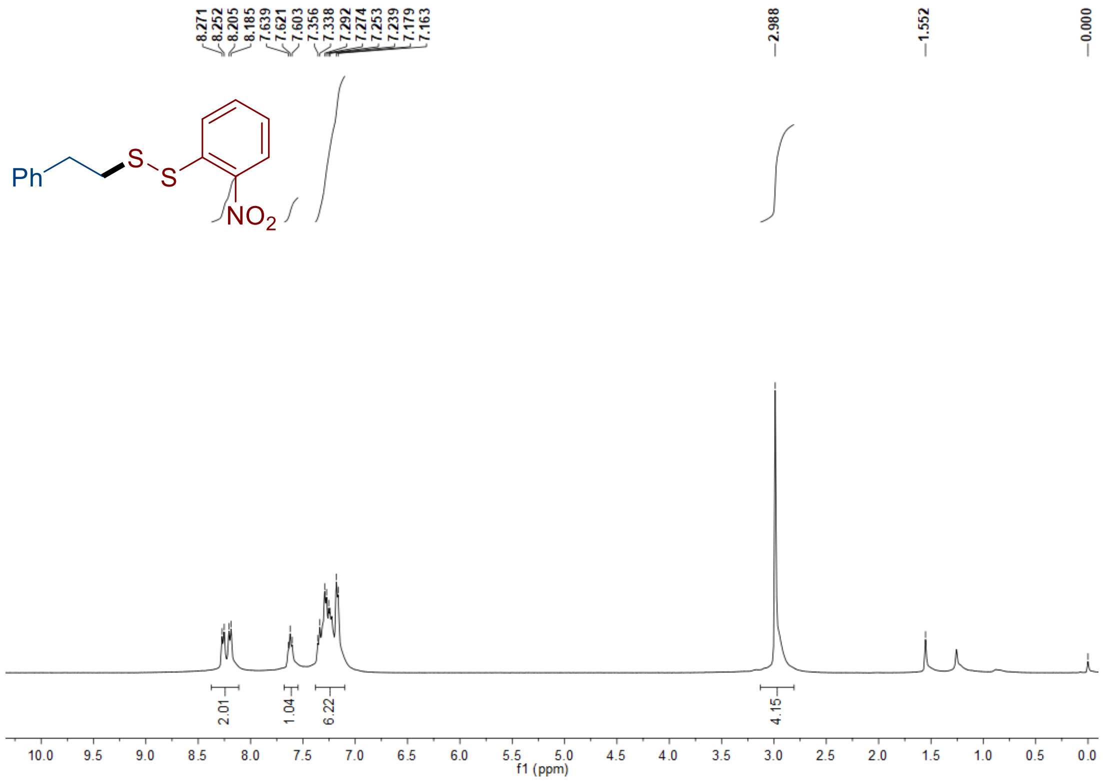

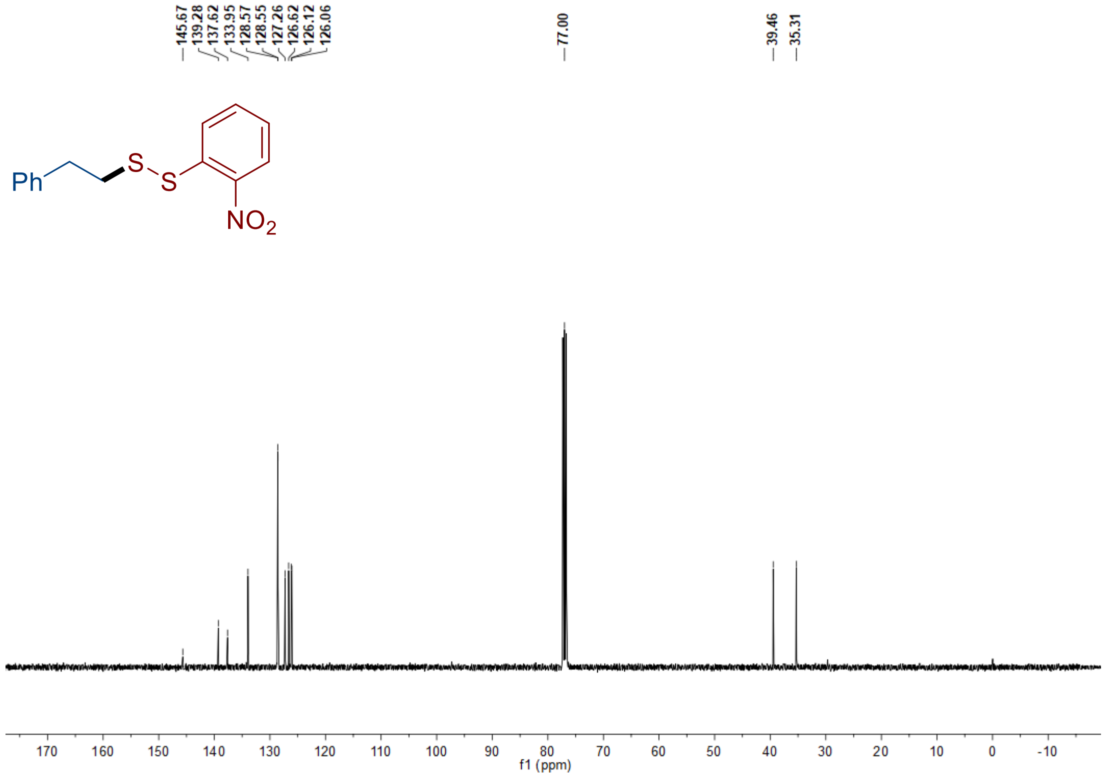

Supplementary Figure 58. ^{1}H and ^{13}C NMR spectra for compound **50**

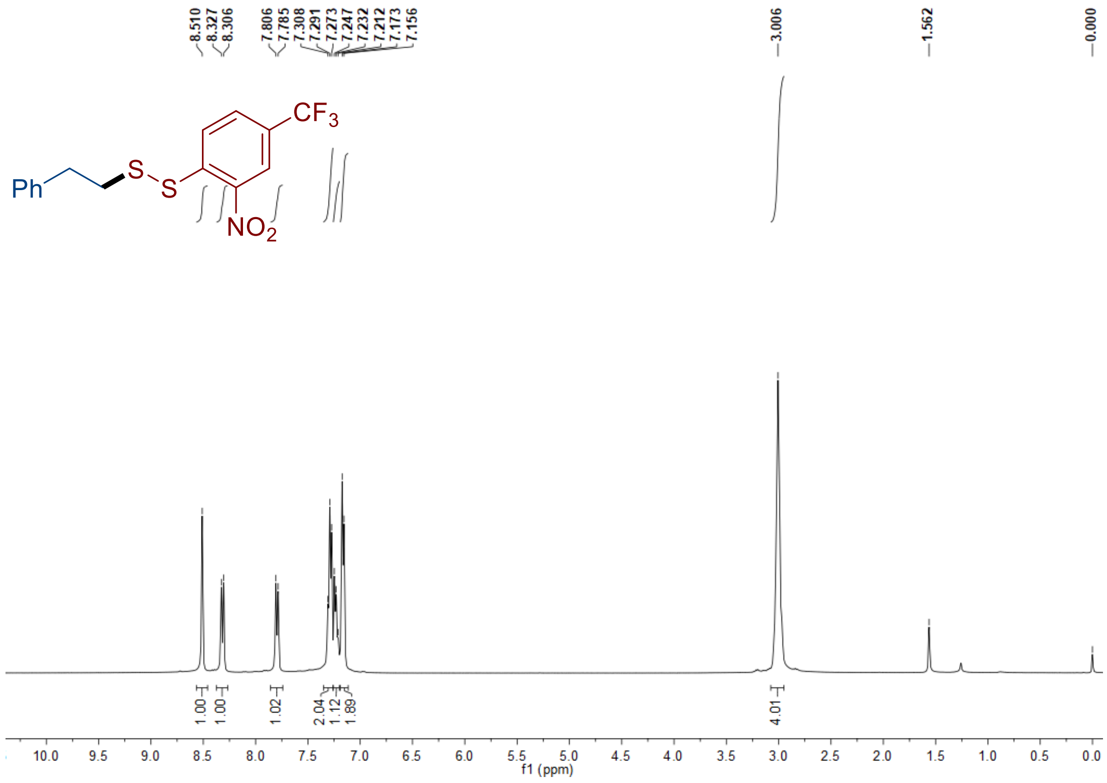

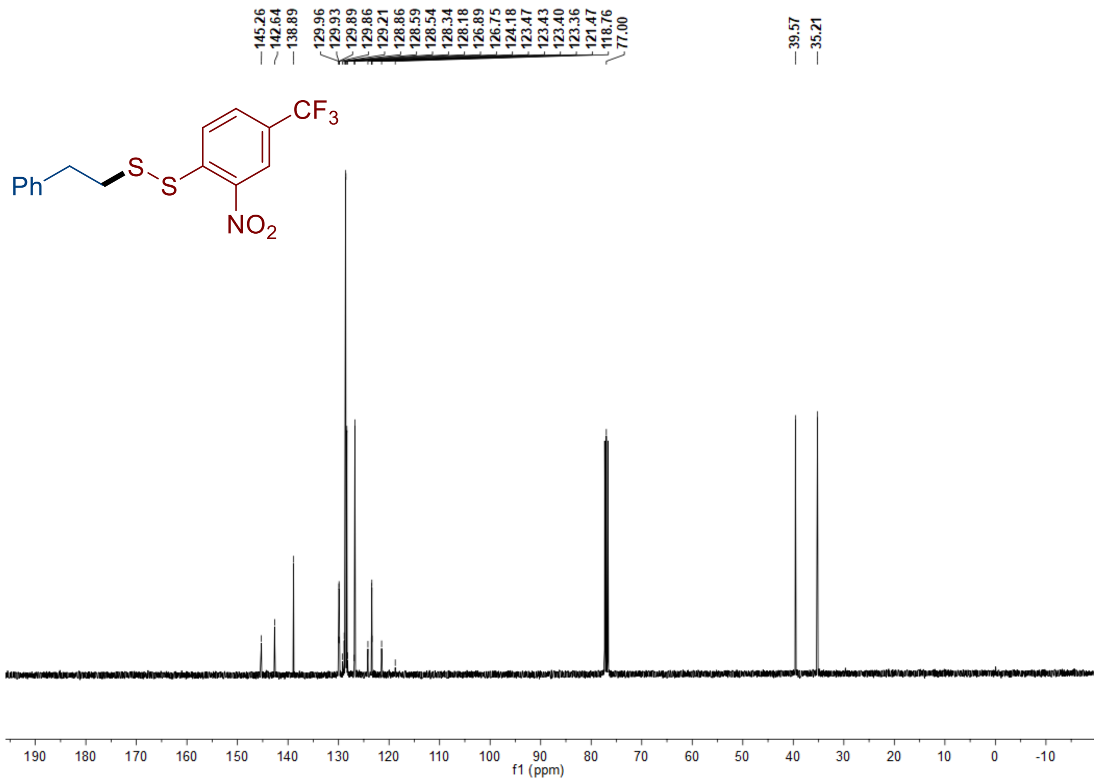

Supplementary Figure 59. ^{1}H and ^{13}C NMR spectra for compound **51**

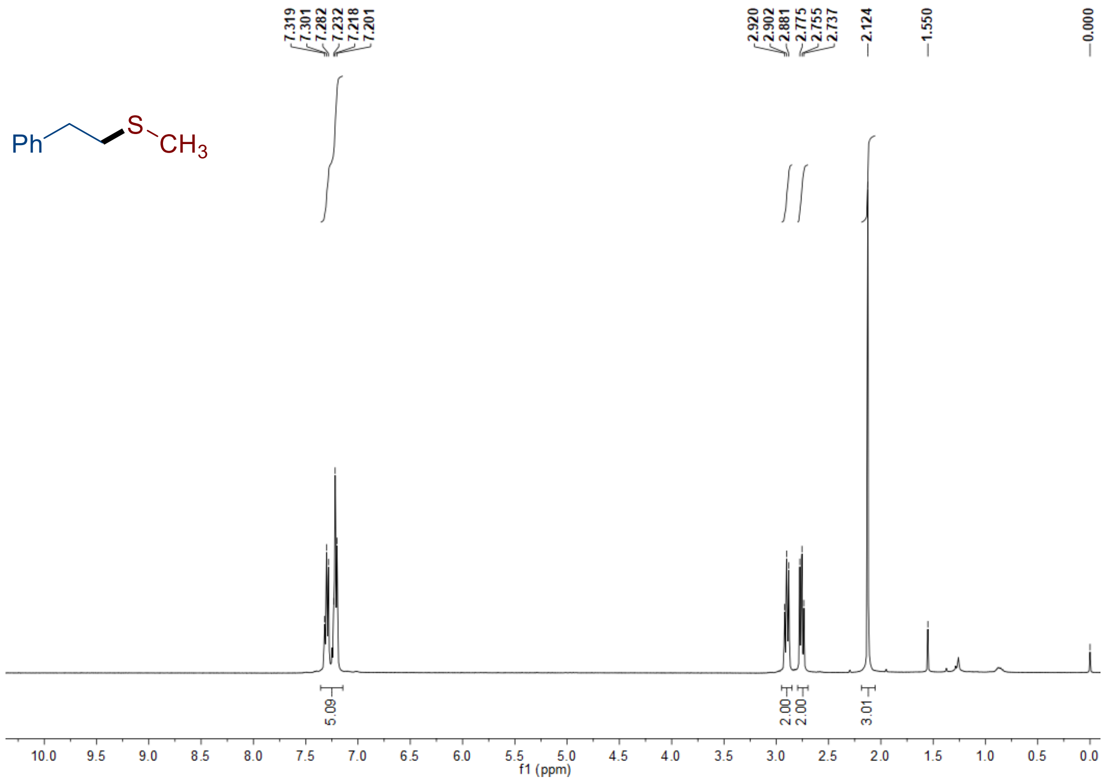

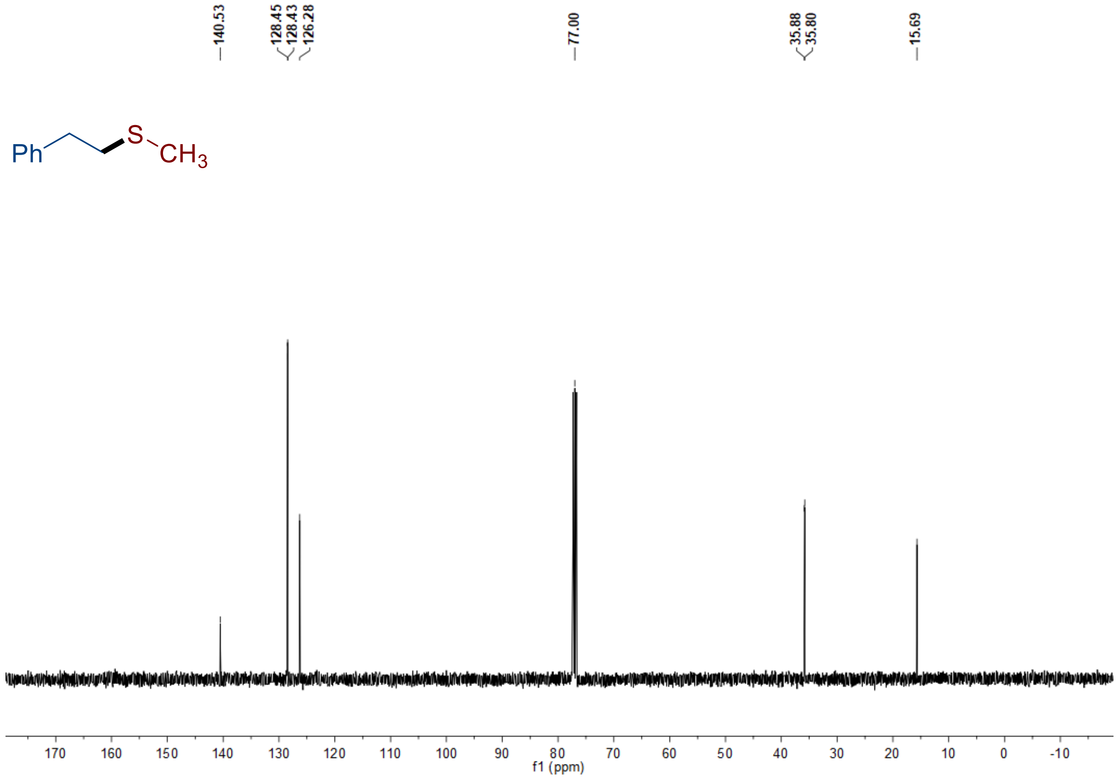

Supplementary Figure 60. ^{1}H and ^{13}C NMR spectra for compound **52**

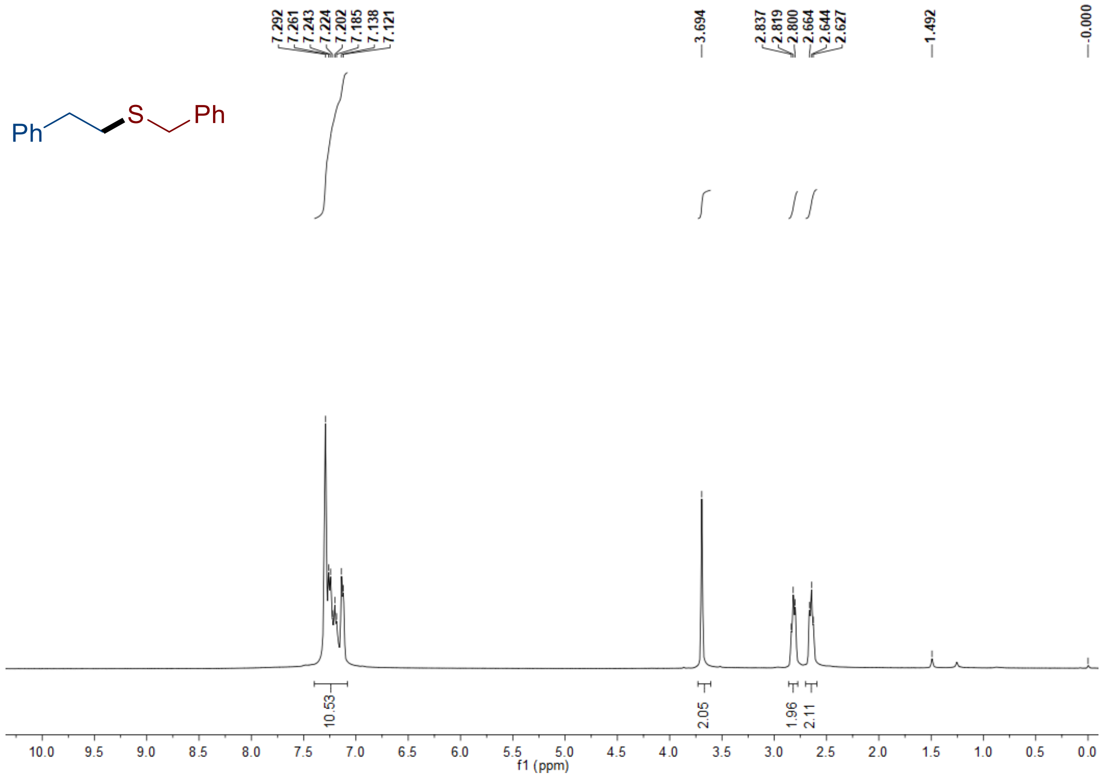

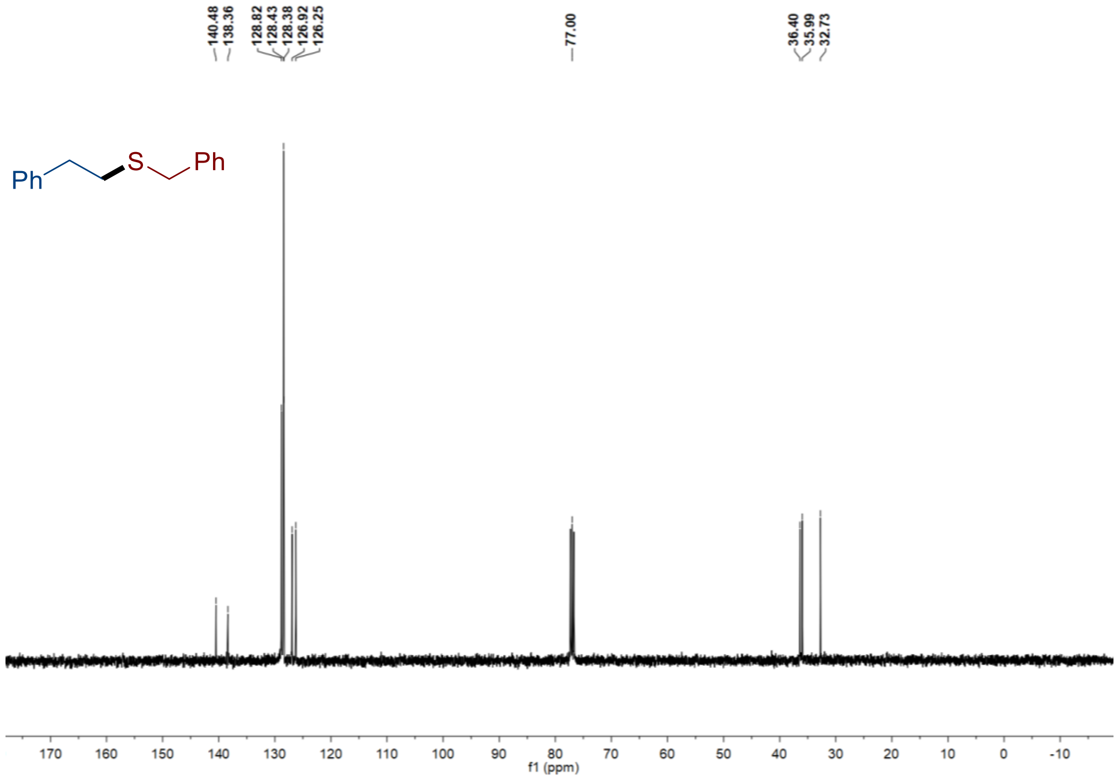

Supplementary Figure 61. ^{1}H and ^{13}C NMR spectra for compound **53**

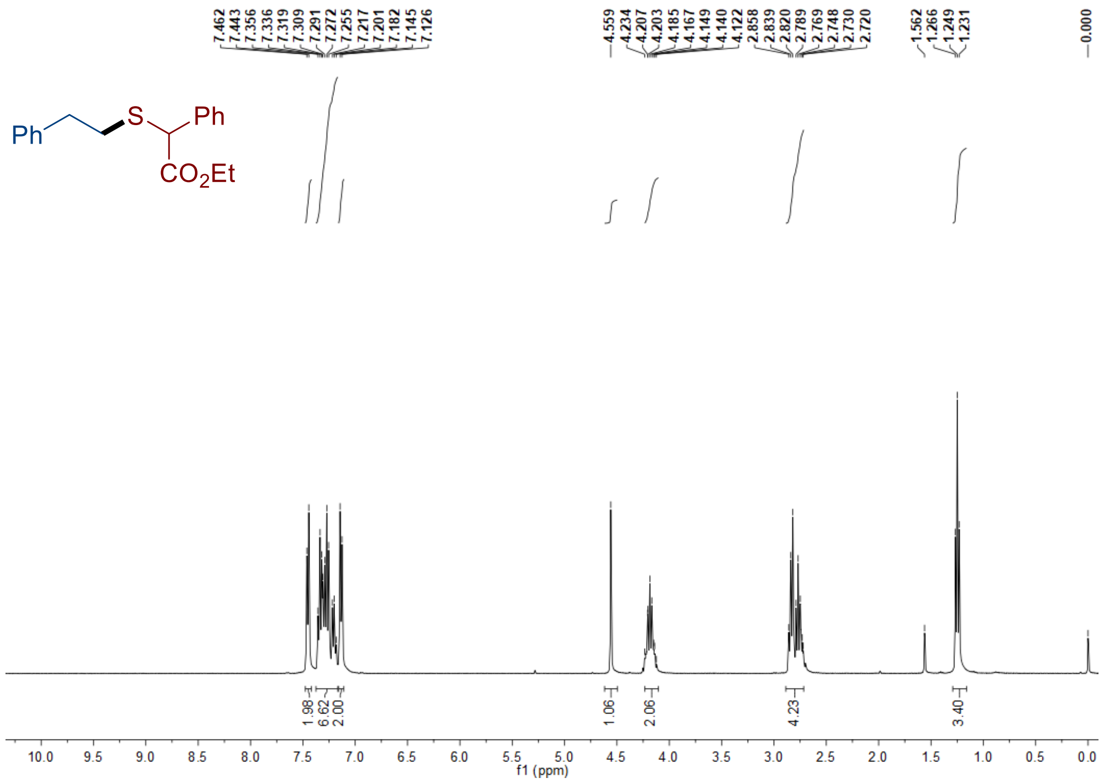

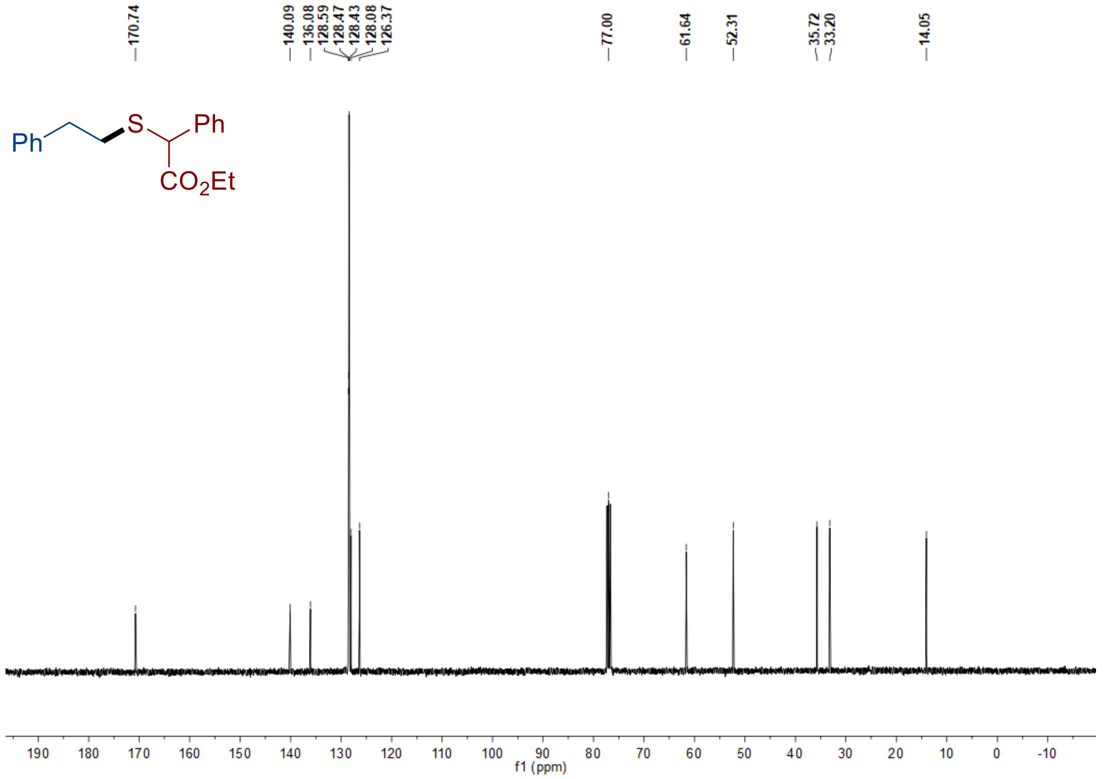

Supplementary Figure 62. ^{1}H and ^{13}C NMR spectra for compound **54**

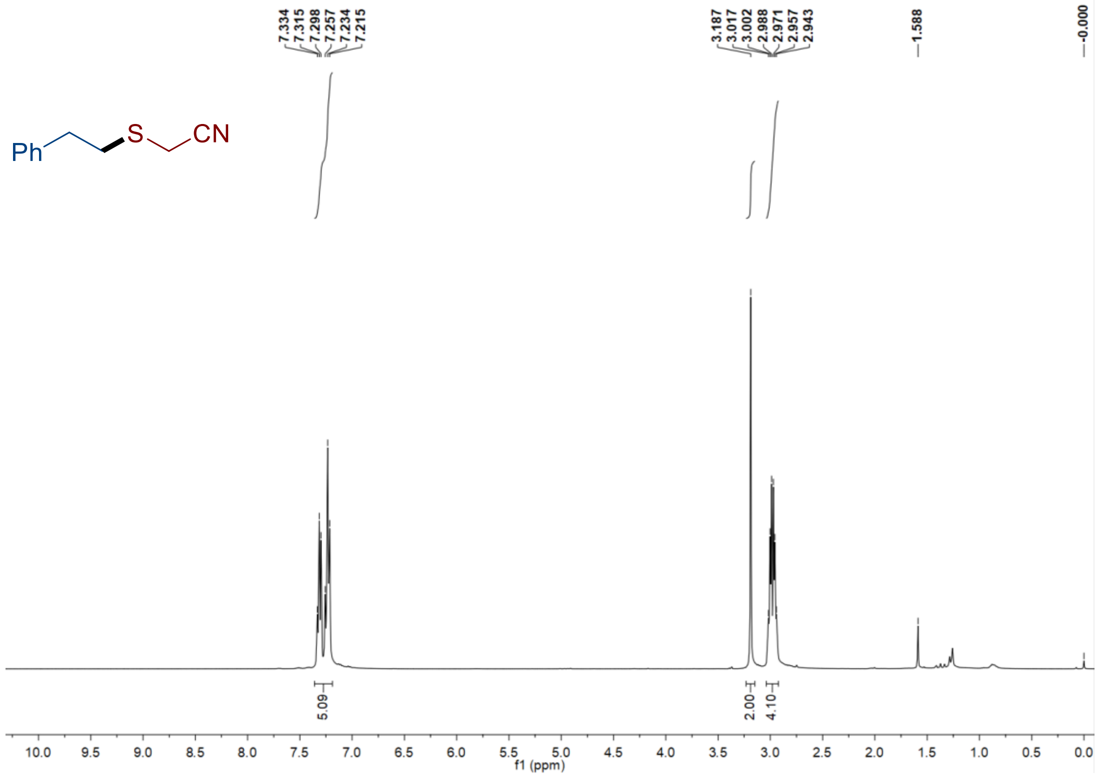

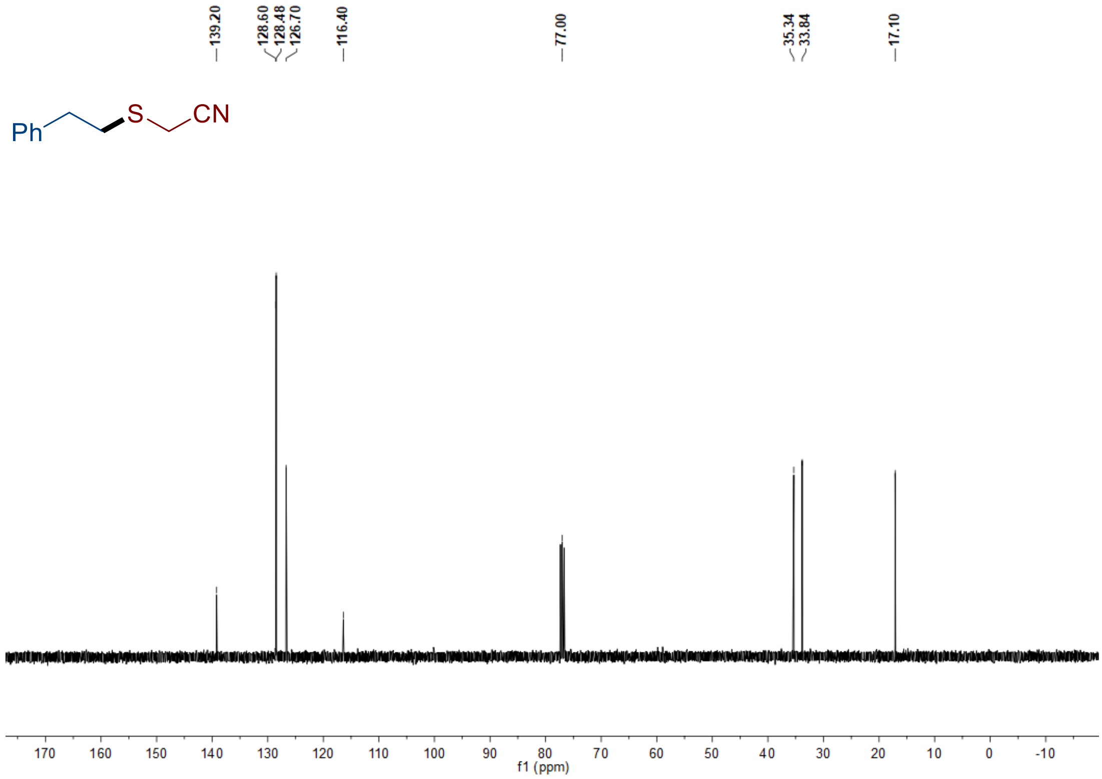

Supplementary Figure 63. ^{1}H and ^{13}C NMR spectra for compound **55**

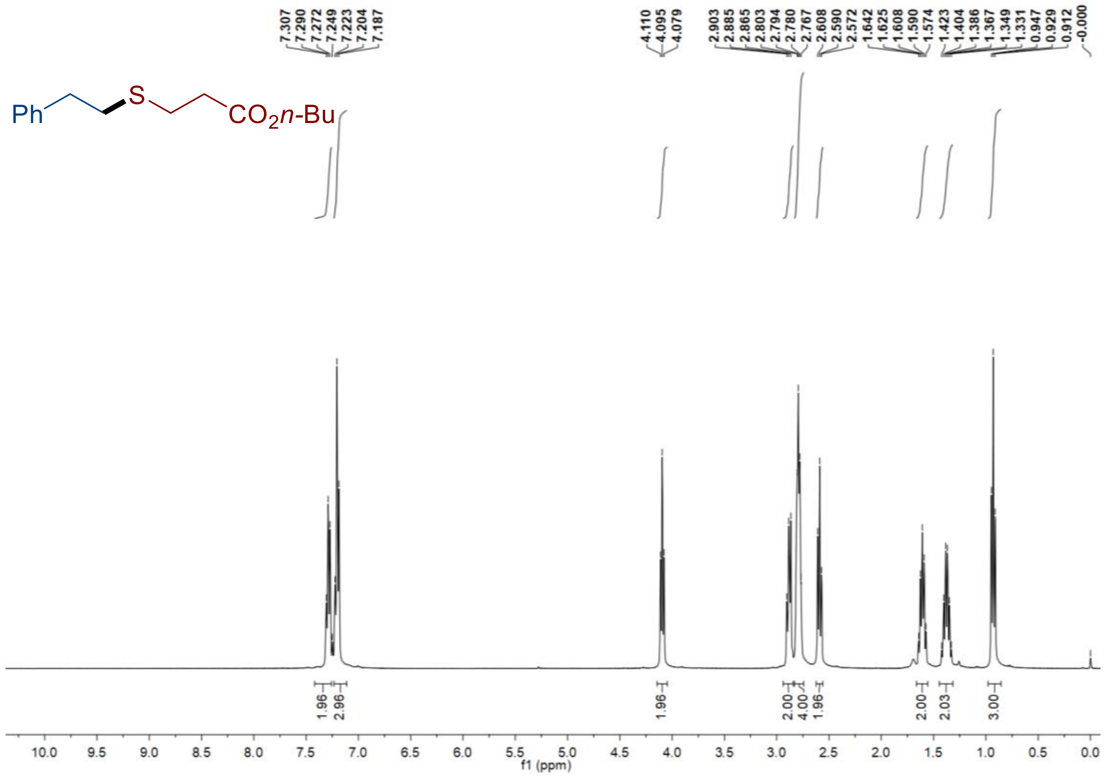

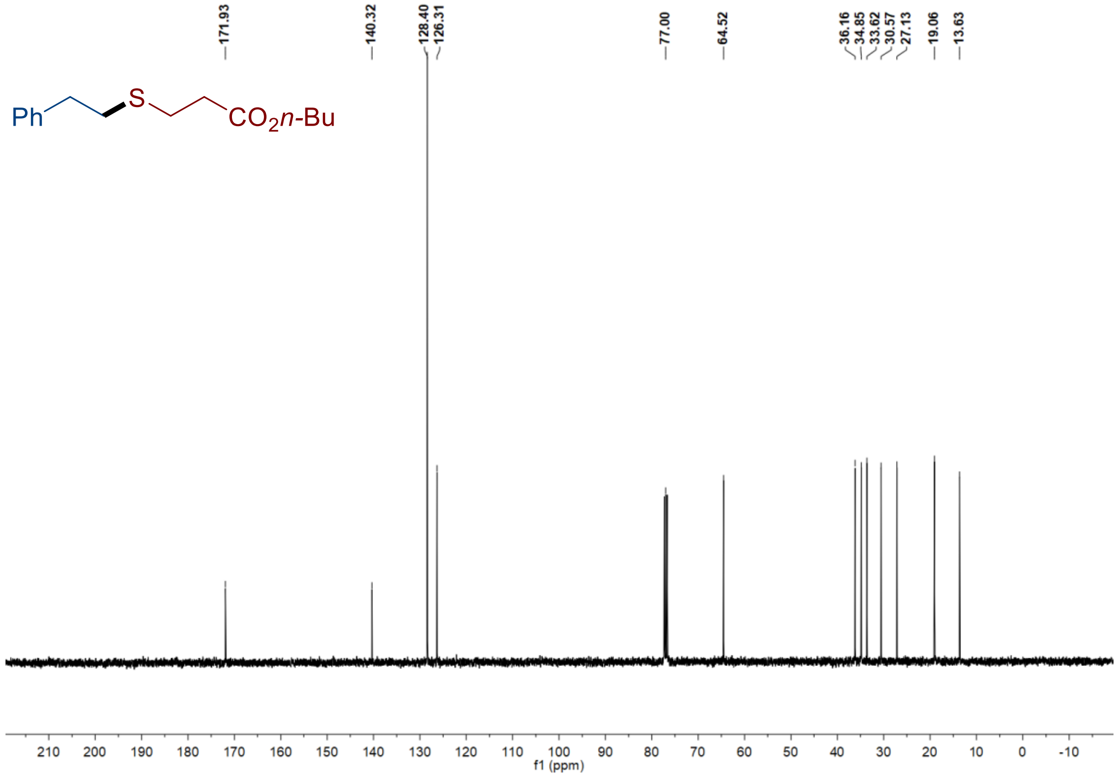

Supplementary Figure 64. ^{1}H and ^{13}C NMR spectra for compound **56**

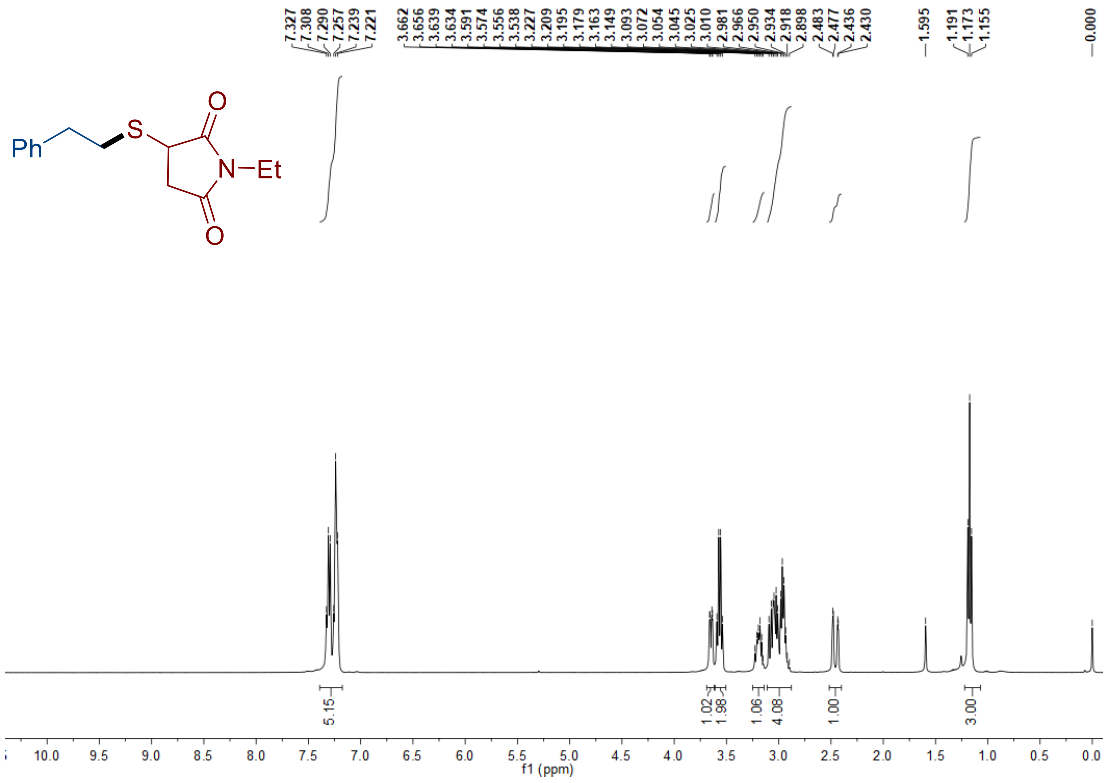

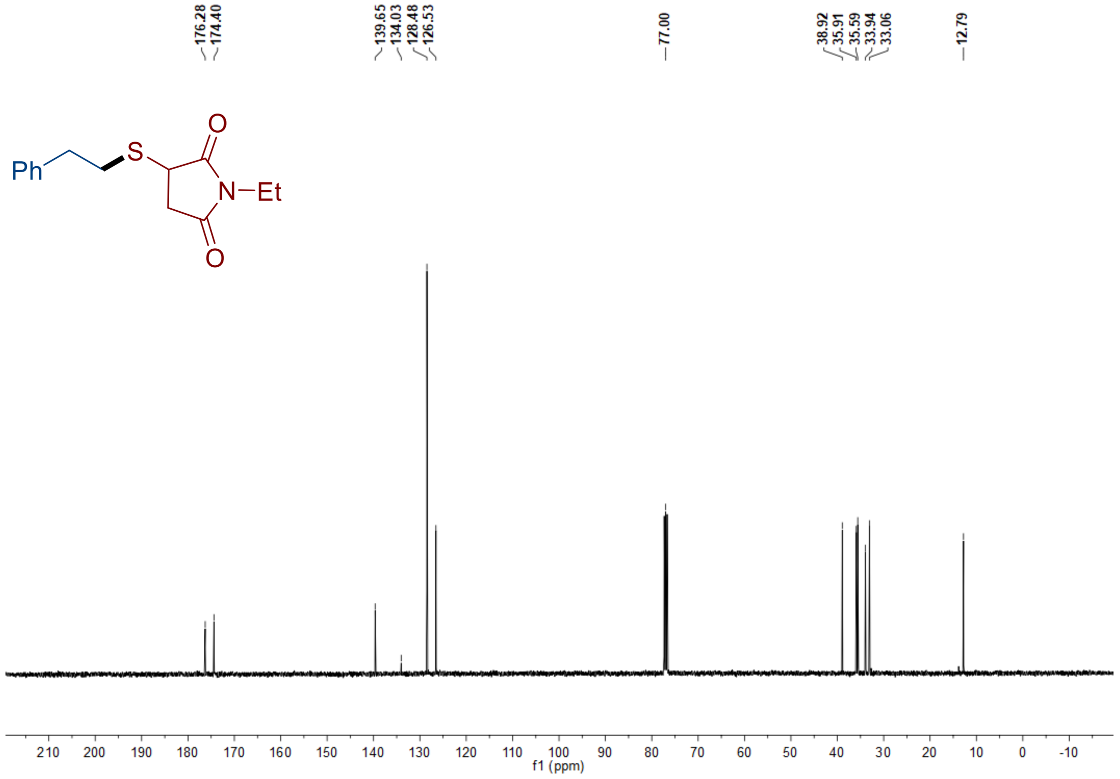

Supplementary Figure 65. ^{1}H and ^{13}C NMR spectra for compound **57**

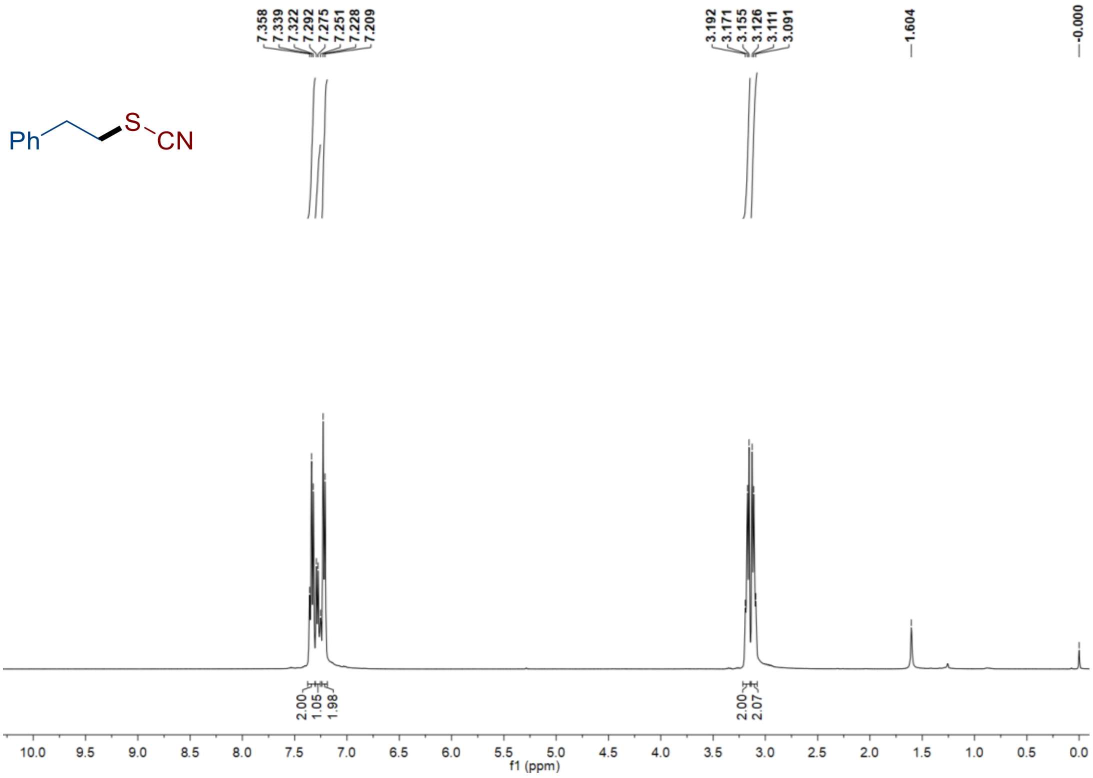

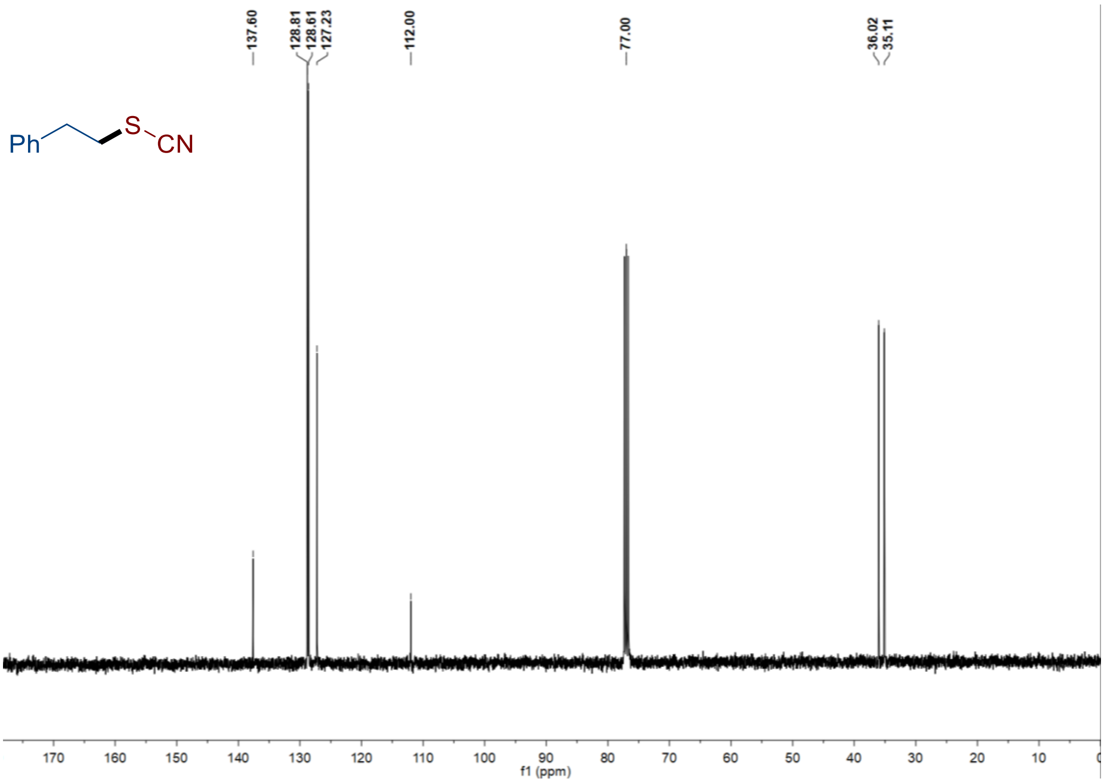

Supplementary Figure 66. ^{1}H and ^{13}C NMR spectra for compound **58**

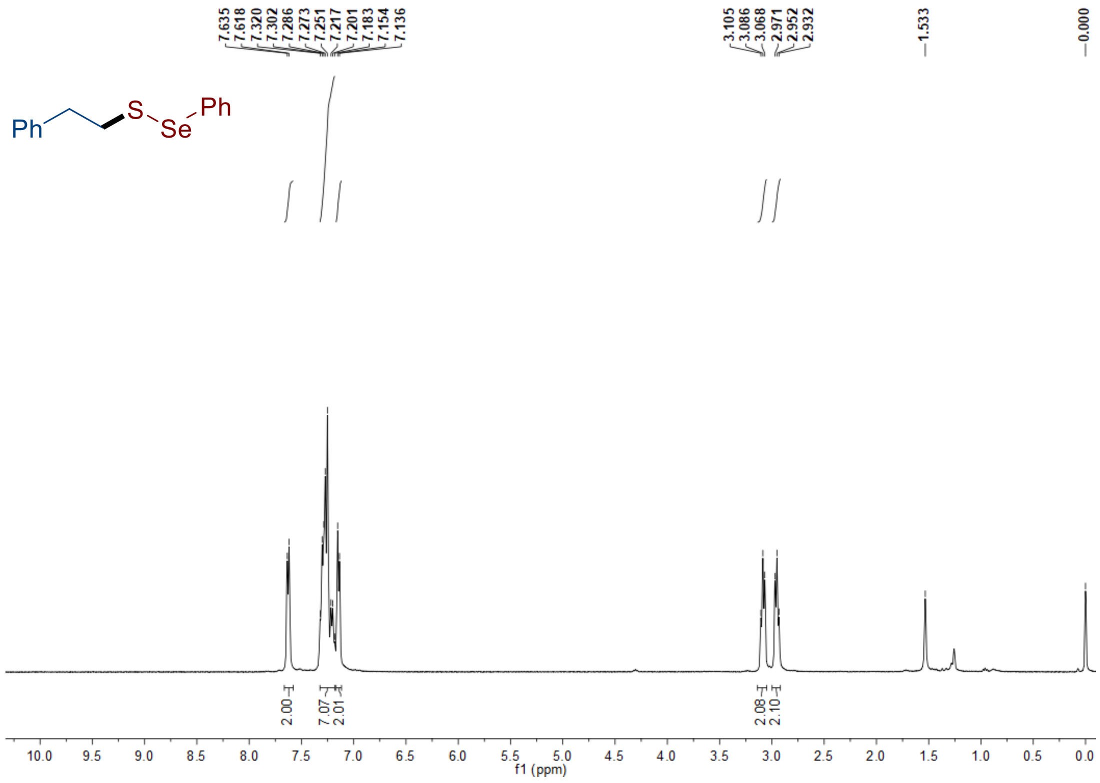

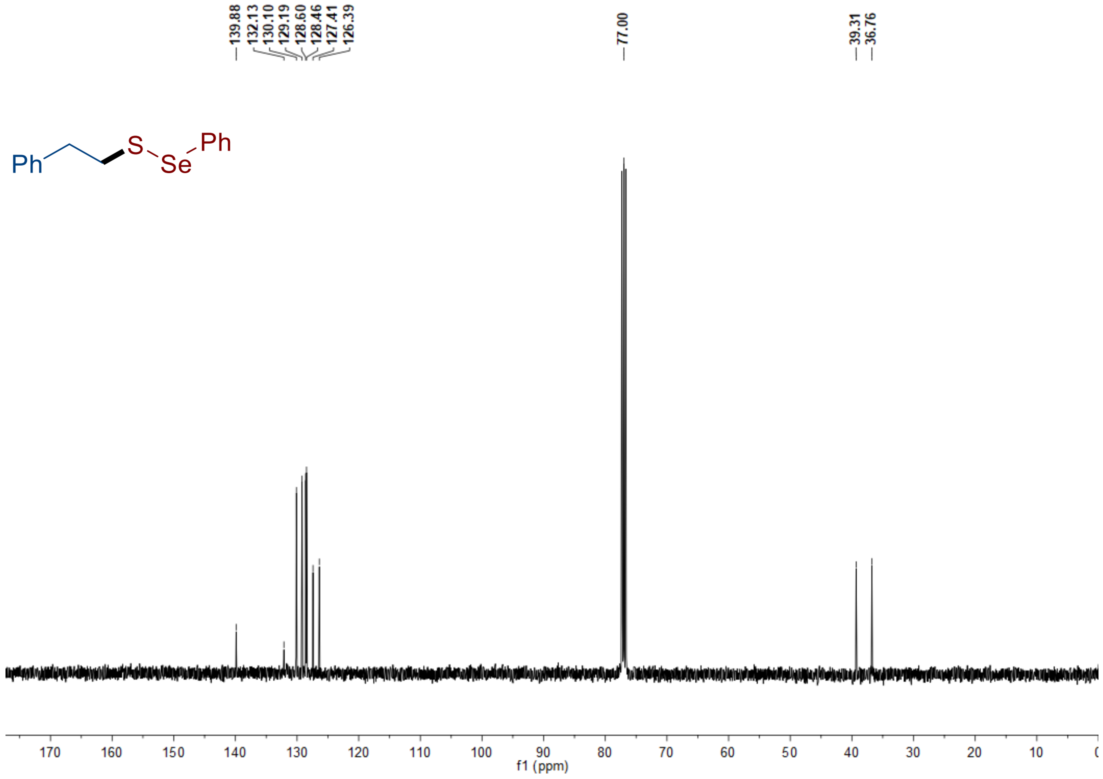

Supplementary Figure 67. ^{1}H and ^{13}C NMR spectra for compound **59**

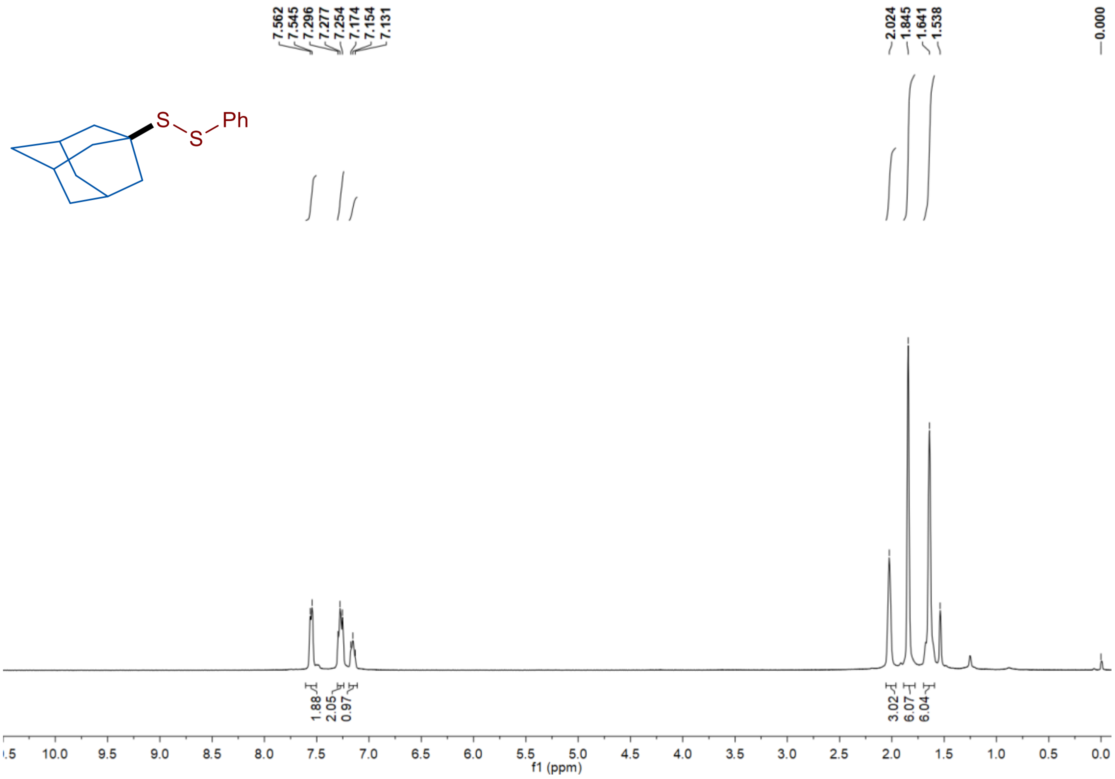

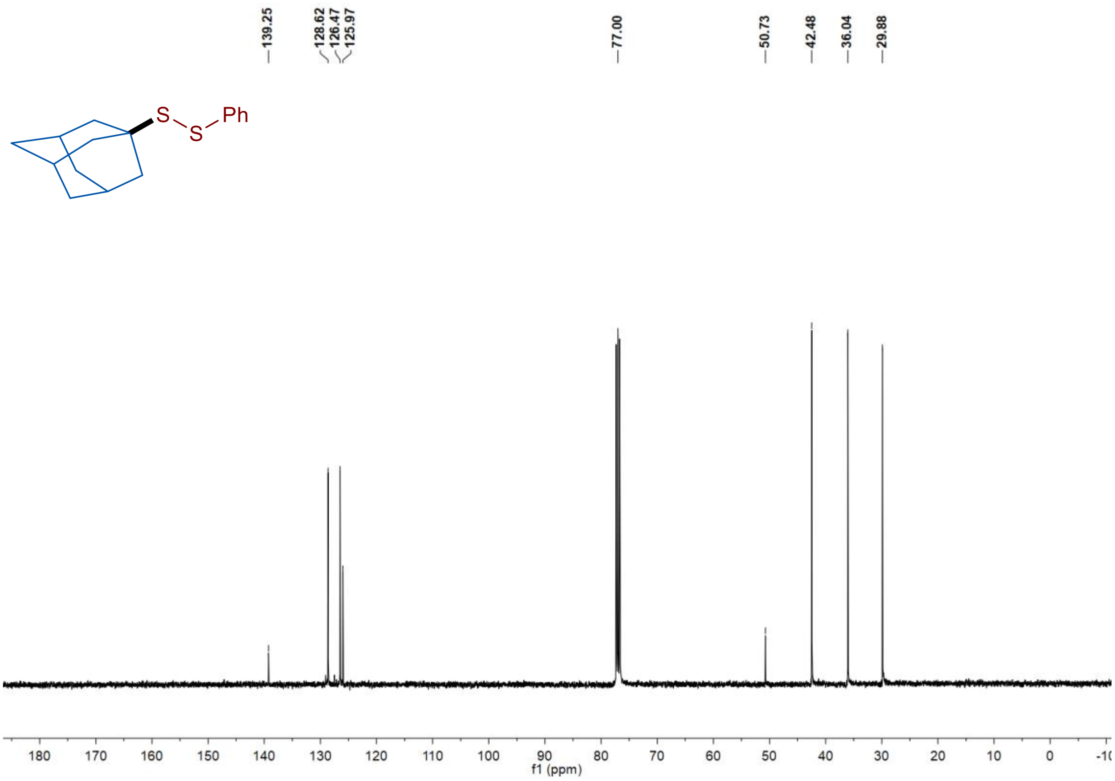

Supplementary Figure 68. ^{1}H and ^{13}C NMR spectra for compound **60**

Supplementary References

1. Xu R., Xu T., Yang M., Cao T., Liao S., A rapid access to aliphatic sulfonyl fluorides. *Nat. Commun.* **10**: 3752 (2019).
2. Mao R., Frey A., Balon J., Hu X., Decarboxylative C(sp^3)–N cross-coupling via synergetic photoredox and copper catalysis. *Nat. Catal.* **1**, 120–126 (2018).
3. Mao R., Balon J., Hu X., Decarboxylative C(sp^3)-O cross-coupling. *Angew. Chem. Int. Ed.* **57**, 13624–13628 (2018).
4. Liang Y., Zhang X., MacMillan D. W. C., Decarboxylative sp^3 C–N coupling via dual copper and photoredox catalysis. *Nature* **559**, 83–88 (2018).
5. Chen H., Hu L., Ji W., Yao L., Liao X., Nickel-catalyzed decarboxylative alkylation of aryl iodides with anhydrides. *ACS Catal.* **8**, 10479–10485 (2018).
6. Schwarz J., König B., Decarboxylative reactions with and without light-a comparison. *Green Chem.* **20**, 323–361 (2018).
7. Huang H., Jia K., Chen Y., Radical decarboxylative functionalizations enabled by dual photoredox catalysis. *ACS Catal.* **6**, 4983–4988 (2016).
8. Barresi E., Nesi G., Citi V., Piragine E., Piano I., Taliani S., Da Settimo F., Rapposelli S., Testai L., Breschi M. C., Gargini C., Calderone V., Martelli A., Iminothioethers as hydrogen sulfide donors: From the gasotransmitter release to the vascular effects. *J. Med. Chem.* **60**, 7512–7523 (2017).
9. Jevtić I. I., Došen-Mićović L., Ivanović E. R., Ivanović M. D., Hofmann rearrangement of carboxamides mediated by N-Bromo-acetamide. *Synthesis* **48**, 1550–1560 (2016).
10. Zhang W., Haskins C., Yang Y., Dai M., Synthesis of nitriles via palladium-catalyzed water shuffling from amides to acetonitrile. *Org. Biomol. Chem.* **12**, 9109–9112 (2014).
11. Koduri N. D., Scott H., Hileman B., Cox J. D., Coffin M., Glicksberg L., Hussaini S. R., Ruthenium catalyzed synthesis of enaminones. *Org. Lett.* **14**, 440–443 (2012).
